# *N*-(2-Aminobenzoyl)benzotriazole Mediated Synthesis of 3-Acyl-2-alkyl(aryl)-4-hydroxyquinolines and 3-Acylamino-4(3*H*) quinazolinones

**DOI:** 10.55730/1300-0527.3642

**Published:** 2023-10-16

**Authors:** İlbilge Merve ŞENOL, Sevtem GÖKBULUT SATIOĞLU, İlhami ÇELİK

**Affiliations:** Department of Chemistry, Faculty of Science, Eskişehir Technical University, Eskişehir, Turkiye

**Keywords:** Covid-19, *N*-(2-Aminobenzoyl)benzotriazoles, benzotriazole, quinoline, quinazolinone

## Abstract

New methods have been developed for the synthesis of the substituted quinolines and quinazolinones derivatives by utilizing *N*-(2-aminobenzoyl)benzotriazoles under mild reaction conditions. 3-Acyl-2-alkyl(aryl)-4-hydroxyquinolines were obtained in modarete yields by the reaction of *N*-(2-aminobenzoyl)benzotriazoles and diketones in the presence of *tert*-BuOK. 3-Acylamino-4(3*H*) quinazolinones were obtained in good yields via *N*-(2-aminobenzoyl)benzotriazoles, orthoester and hyrazides in one-pot.

## 1. Introduction

*N*-Heterocyclic compounds are the most common skeletons in substances such as various synthetic drugs, bioactive natural products and pharmaceuticals. Due to their widespread application, these skeletons have long attracted great interest and facilitated the development of methods that will enable the synthesis of new biological activity molecules in medical chemistry [1]. Quinolinone and quinazolinone derivatives are important classes of nitrogenous heteroaromatic compounds that exhibit broad biological and pharmacological activity.

**Figure 1:**
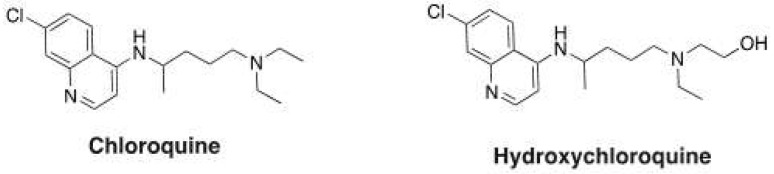
Chemical structures of chloroquine and hydroxychloroquine.

It has been reported that quinolinone derivatives [2] show several activities like antimicrobial [3], antiinflammatory, anticancer [4] and anticonvulsant [5]. Compounds with quinoline ring bearing acyl group in C3 position show important biological activity such as antiparasitic against Leishmania infantum [6], antitumor by inhibiting Hedgehog signalling pathway [7], antiviral against HIV-1 [8]. Moreover, chloroquine and hydroxychloroquine (Figure 1) which are quinoline derivatives used as an antimalarial drug; have been recently the subject of many in vitro and clinical studies for the treatment of Covid-19 [9–11].

**Scheme 1:**
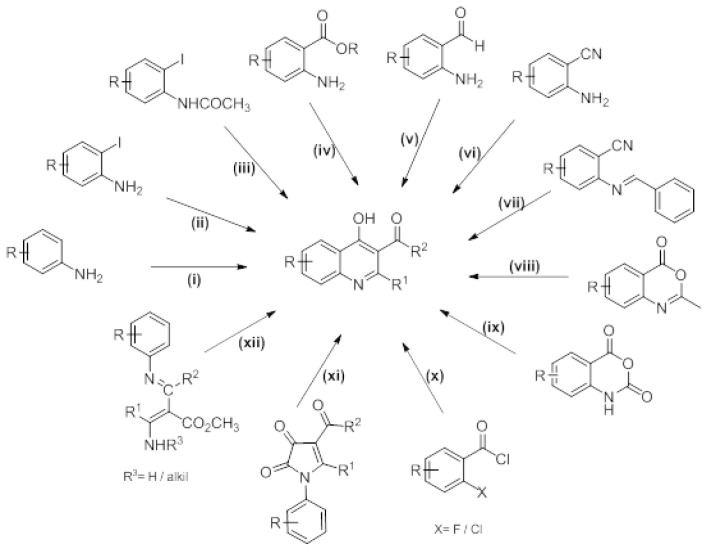
Literature methods for preparing of 3-acyl-2-alkyl(aryl)-4-hydroxyquinoline.

Acyl-2-alkyl(aryl)-4-hydroxyquinolines having ketone groups in the C3 position were synthesized by various methods like; i) the reaction of aniline with acrylates [12], ii) the reaction of iodo aniline with α,β-unsaturated ketones [7], iii) the cyclization of aromatic enaminones formed by the reaction of iodobenzene with 4-substituted isoxazole by heating in an acidic medium [13] iv) the reaction of methyl-2-aminobenzoates with α,β-unsaturated ketones [14] v) the reaction of 2-aminobenzaldehyde with various alkynes [15], vi) the hydrolysis of the intermediate product formed by the reaction of 2-aminobenzonitrile with 1,3-diketones [16], vii) reaction of 2-[(benzylidine)amino]benzonitrile with various phosphorus ylides, viii) the reaction of 2-substituted benzoxazinon with 1,3-diketones in basic medium [17], ix) the reaction of isotoic anhydride with 1,3-diketones [18], x) Reaction of *o*-halogenobenzoyl chlorides and β-ketoenamines in basic (Et_3_N or DBU) medium [19] xi) the thermolysis reaction of *N*-arylpyrrole-2,3-diones prepared by various methods at high temperatures [20], xii) the condensation reaction of *β*-amino-*α*-(*N*-arylimidoyl)crotonates in phosphoric acid [21] (Scheme 1).

There are many methods in the literature for the synthesis of 3-acyl-2-alkyl(aryl)-4-hydroxyquinoline; however these methods are associated with disadvantages such as harsh reaction conditions, long reaction steps, the use of expensive reagents, and the use of catalysts.

The quinazolinone ring is a heterocyclic compound formed by fusing benzene and pyrimidone [22]. It is the building block of about 200 alkaloids [23] isolated from different sources such as plants [24] and microorganisms [25]. Natural and synthetic quinazolinone are important for organic and medicinal chemistry owing to their effects such as anticancer [26] antimalarial [27], antifungal [28], antihyperlipidemic [29].

**Scheme 2:**
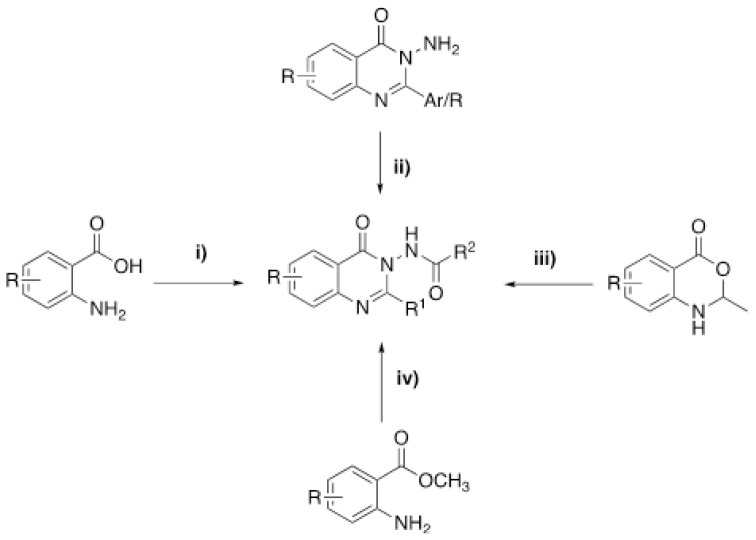
Literature methods for preparing of 3-acylamino-4(3*H*) quinazolinones.

In previous studies 3-acylamino-4(3*H*) quinazolinones were synthesized by; i) the reaction of anthranilic acid, acetic anhydride and primary amine in the presence of nano-TiO_2_ [30], ii) the reaction of 3-amino-aryl/alkyl-4(3*H*) quinazolinone and an orthoester or acylchlorides in pyridine or benzene [31], iii) the reaction of benzoxazione and dicarboxylic acid dihydrazides [32], iv) adding acyl chloride to the intermediate product formed by heating methyl-2-amino benzoate with hydrazine [33] (Scheme 2).

Although there are many methods in the literature for the synthesis of 3-acylamino-4(3*H*) quinazolinones, these methods have harsh reaction conditions, multiple reaction steps, and the use of catalysts. For this reason, mild reaction conditions are needed for the synthesis of these compounds.

*N*-(2-aminobenzoyl) benzotriazole compounds, which are derivatives of *N*-acyl benzotriazole, have many benefits such as being crystalline, dissolving in many organic solvents, not absorbing moisture, and being stable. Besides, these compounds are used as starting materials in the synthesis of anthranylesters and anthranylthioesters [34], anthranilamides [35] and some heterocyclic compounds [36–38].

## 2. Results and discussion

### 2.1. Preparation of 3-Acyl-2-alkyl(aryl)-4-hydroxyquinoline (3a–j)

*N*-(2-aminobenzoyl)benzotriazoles 1a–1j were prepared by the method in our previous study [35]. After *N*-(2-aminobenzoyl)benzotriazoles 1a–1j were synthesized, the synthesis of 3-acyl-2-alkyl(aryl)-4-hydroxyquinolines 3a–3j was started with the proposed method. First of all, the synthesis of 3-acyl-2-methyl-4-hydroxyquinoline 3a was tried under different reaction conditions to find the appropriate reaction conditions in the presence of *tert*-BuOK. This model reaction was tried in different solvents, at room temperature and under reflux conditions (Table 1). The highest yield for 3-acyl-2-methyl-4-hydroxyquinoline 3a was obtained when the reaction was performed in dioxane and heated. After the reaction conditions were optimised, the synthesis of the other compounds in the series was carried out with 14%–59% yield (Table 2).

The structures of synthesized compounds were elucidated by ^1^H, ^13^C-NMR, HRMS and FTIR spectroscopy techniques. The characteristic singlet signal observed in downfield ^1^H NMR spectrum is thought to belong to the -OH proton. Since these compounds contain a free acidic proton in their structure, they have two possible tautomeric structures having 4-hydroxyquinoline 3a and 4-oxoquinoline 3a′. The characteristic singlet peak observed in the range of 10.90–12.24 ppm is considered to belong to the OH or NH proton. In our previous study, ^1^H-^15^N HSQC experiment was performed to determine the OH and NH protons in quinolines with similar structures that we synthesized [32]. According to the results of the experiment, it was observed that the OH proton appeared at around 12 ppm, and the NH proton at around 9 ppm. From the results of this NMR experiment, the characteristic singlet peak observed in the range of 10.90–12.24 ppm is thought to belong to the OH proton. When the ^13^C-NMR spectra of the compounds were examined, the signals of the carbonyl carbons in the acyl group at position three were observed at 195.8–202.2 ppm.

**Scheme 3:**
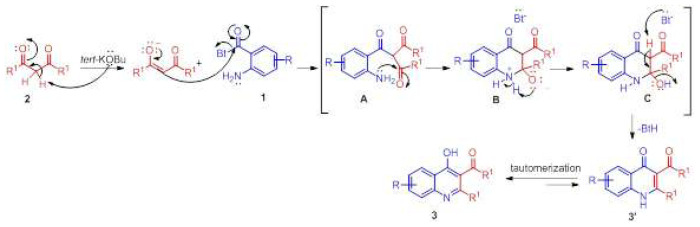
Possible reaction mechanism for 3-acyl-2-alkyl(aryl)-4-hydroxyquinolines.

A possible reaction mechanism according to the formation of annulation product is proposed in Scheme 3. The reaction would be initiated by addition of the enol structure formed by the removal of the acidic hydrogen atom of the 1,3-dicarbonyl compound by *tert*-BuOK to *N*-(2-aminobenzoyl)benzotriazole. Then, the subsequent cyclization of formed intermediate A affords intermediate B and benzotriazolyl anion. The intermediate C is formed by proton exchange within the intermediate B itself. With the removal of the hydrogen atom in the C intermediate by the benzotriazolyl anion, the product 3′ is formed and the tautomerization of desired product produces the product 3.

**Scheme 4:**
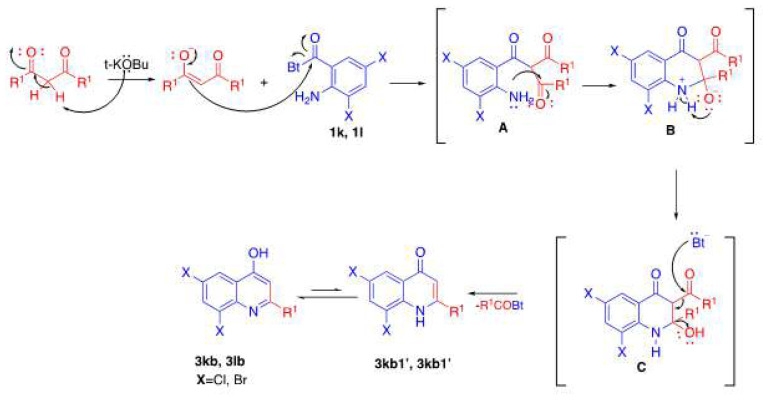
Possible reaction mechanism for by-products.

The desired compounds in Table 3 were synthesized with moderate yields. The desired compounds 3k and 3l could not be obtained at the end of the reaction. Instead of these compounds, by-products 3kb and 3lb shown in the following the reaction mechanism (Scheme 4) were obtained. The structures of the by-products were elucidated with both ^1^H-NMR and ^13^C-NMR spectra. When the ^1^H-NMR spectra of the by-products are examined, the absence of signals belonging to the acyl group and the absence of the signal belonging to the carbonyl group, which is observed around 200 ppm in the ^13^C-NMR spectrum, supports the formation of by-products.

When the possible reaction mechanism for the obtained by-products 3kb and 3lb is examined, intermediate C is thought to be formed by the addition of the benzotriazolyl anion to the carbonyl group and then leaving as the *N*-acylbenzotriazolyl group as shown in Scheme 4.

### 2.2. Preparation of 3-acylamino-4(3H) quinazolinones (6a–h)

**Scheme 5:**
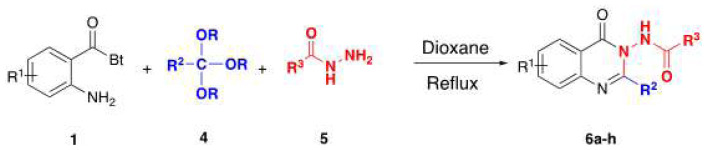
Method for the preparation of 3-acylamino-4(3*H*) quinazolinones.

3-Acylamino-4(3*H*) quinazolinones 6 were obtained in 34%–84% (Table 3) yield by refluxing *N*-(2-aminobenzoyl) benzotriazoles 1, orthoesters 4 and hydrazides 5 in dioxane for 18–20 h (Scheme 5).

**Figure 2:**
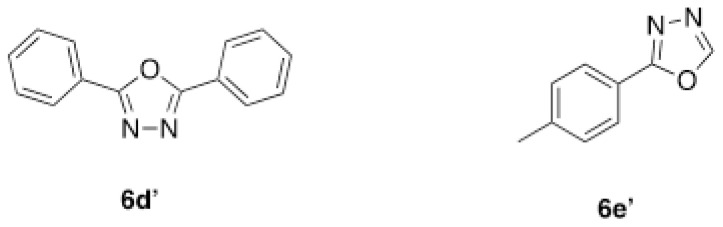
Chemical structures of by-products.

Structures of obtained products were idendified by ^1^H NMR, ^13^C NMR, HRMS and FTIR spectroscopy techniques. A characteristic singlet observed around 10 ppm in the ^1^H NMR spectra was assigned to the hydrogen atom bound to nitrogen adjacent to N3. The hydrogen atom on C2 was observed between 8.23 ppm and 8.51 ppm (Supplemental information) For compounds 6d and 6h, no signal was observed around 8 ppm because of the substituent at the position 2. The ^13^C NMR spectra of 6a–h showed new signals at 165.8–159.2 ppm as well as at 163.0–156.4 ppm, corresponding to carbonyl carbon at the position 4 and amidoyl (carbamoyl) carbon attached to the nitrogen at position 3 in the quinazoline ring, respectively. Moreover, HRMS and FTIR spectral data were also appropriately with the proposed structures. During the preparation of 3-acylamino-4(3*H*) quinazolinones 6a–6h, it was noticed that a by-products 6d′ and 6e′ (Figure 2) formed with the expected products. The by-products 6d′ and 6e′ were isolated by column chromatography in 36% and 78% yields respectively. The structures of the by-products shown in Figure 2 were elucidated by ^1^H, ^13^C NMR and HRMS.

**Scheme 6:**
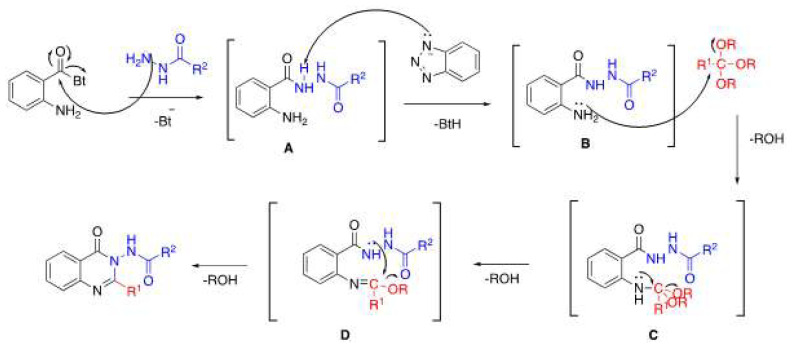
Possible reaction mechanism for 3-acylamino-4(3*H*) quinazolinones.

A possible mechanism for the formation of 3-acylamino-4(3*H*) quinazolinones is proposed in Scheme 6. The reaction will be initiated by the addition of hydrazides to the carbonyl of the *N*-(2-aminobenzoyl)benzotriazoles. Intermediate B will be formed by removing the hydrogen atom of the benzotriazolyl group from the intermediate A formed. The product is formed as a result of the subsequent cyclization of intermediate D, which is formed as a result of the addition of intermediate B to the orthoester.

## 3. Experimental section

### 3.1. General information

NMR spectra of the synthesized products were recorded in DMSO-*d6* or CDCl_3_, at 400 MHz for ^1^H NMR, 100 MHz for ^13^C NMR (Agilent 4 DD2 400 MHz spectrometer). Melting points were determined with Mettler Toledo MP90 apparatus and were uncorrected. HRMS spectra were recorded with Shimadzuhybrid LC-MS-IT-TOF spectrometer. IR spectra were recorded with Perkin Elmer 100 FTIR. Necessary drying processes were applied to the solvents used during the synthesis and purification of the compounds.

### 3.2. General method for the synthesis of 3-Acyl-2-alkyl(aryl)-4-hydroxyquinoline (3a–3j)

Substituted *N*-(2-aminobenzoyl) benzotriazoles 1 (0.25 mmol) and 1,3-diketones 2 (0.25 mmol) were mixed in 5 mL of dioxane for 15 min. *tert*-BuOK 0.25 mmol) was added to the mixture and refluxed for 24 h. Reactions were monitored by thin layer chromatography (TLC) under UV light. After the reaction is complete, the solvent was removed under reduced pressure. The reaction mixture was purified by column chromatography with EtOAc/Hexane (1/1).

#### 1-(4-Hydroxy-2-methylquinolin-3-yl)ethanone (3a)

Brown solid (30 mg, 59%), mp.: > 230 ^o^C (decomposed). ^1^H NMR (400 MHz, DMSO-*d_6_* ): δ 2.40 (s, 3H), 3.32 (s, 3H), 7.33 (t, *J* = 7.4 Hz, 1H), 7.51 (d, *J* = 8.0 Hz, 1H), 7.65 (t, *J* = 7.6 Hz, 1H), 8.08 (d, *J* = 8.0 Hz, 1H), 11.90 (s, 1H). ^13^C NMR (100 MHz, DMSO-*d_6_* ): δ 19.4, 32.4, 118.4, 120.7, 124.5, 125.6, 125.8, 132.8, 139.2, 152.0, 175.8, 202.1. δ FTIR u_max_ (KBr): 757, 1348, 1511, 1550, 1673, 3020 cm^−1^. HRMS (ESI): *m/z* [M+H]^+^ calcd for C_12_H_11_NO_2_ 202.0863; found *m/z* 202.0858.

#### (4-Hydroxy-6-methyl-2-phenylquinolin-3-yl)(phenyl)methanone (3b)

White solid (22 mg, 26%), mp.: > 292 ^o^C (decomposed). ^1^H NMR (400 MHz, DMSO-*d_6_* ): δ 2.41 (s, 3H), 7.43–7.37 (m, 7H), 7.57–7.50 (m, 2H), 7.63 (d, *J* = 8.4 Hz, 1H), 7.74 (d, *J* = 6.8 Hz, 2H), 7.86 (s, 1H), 12.05 (s, 1H). ^13^C NMR (100 MHz, DMSO-*d_6_* ): δ 21.2, 119.2, 120.4, 124.5, 125.1, 128.9, 129.0, 129.1, 129.4, 130.4, 133.4, 133.8, 134.1, 134.3, 138.3, 138.4, 149.5, 175.3, 196.3. FTIR u_max_ (KBr): 694, 899, 1361, 1499, 1570, 1672, 2864 cm^−1^. HRMS (ESI): *m/z* [M+H]^+^ calcd for C_23_H_17_NO_2_ 340.1332; found *m/z* 340.1332.

#### (4-Hydroxy-8-methyl-2-phenylquinolin-3-yl)(phenyl)methanone (3c)

Pale yellow solid (19 mg, 22%), mp.: > 290 ^o^C (decomposed). ^1^H NMR (400 MHz, DMSO-*d_6_*): 2.58 (s, 3H), 7.29 (t, *J* = 7.6 Hz, 1H), 7.40–7.34 (m, 5H), 7.44 (dd, *J* = 8.0, 1.6 Hz, 2H), 7.50 (d, *J* = 7.2 Hz, 1H), 7.56 (d, *J* = 6.8 Hz, 1H), 7.71 (d, *J* = 8.4 Hz, 2H), 7.95 (d, *J* = 8.4 Hz, 1H), 10.90 (br s, 1H). ^13^C NMR (100 MHz, DMSO-*d_6_* ): δ 18.2, 121.1, 123.2, 124.1, 125.5, 128.0, 128.6, 129.0, 129.3, 129.5, 130.2, 133.4, 133.9, 134.2, 138.2, 139.1, 150.2, 175.6, 196.1. FTIR u_max_ (KBr): 694, 899, 1361, 1499, 1570, 1672, 2864 cm^−1^. HRMS (ESI): *m/z* [M+H]^+^ calcd for C_23_H_17_NO_2_ 340.1332; found *m/z* 340.1326.

#### 1-(4-Hydroxy-6,7-dimethoxy-2-methylquinolin-3-yl)ethanone (3d)

Brown Solid (26 mg, 40%), mp. > 260 ^o^C (decomposed). ^1^H NMR (400 MHz, DMSO-*d_6_* ): 2.37 (s, 3H), 2.47 (s, 3H), 3.80 (s, 3H), 3.83 (s, 3H), 7.16 (s, 1H), 7.43 (s, 1H). ^13^C NMR (100 MHz, DMSO-*d_6_* ): δ 19.5, 32.5, 55.9, 56.2, 100.2, 104.8, 119.6, 119.8, 135.2, 147.3, 150.7, 153.4, 174.7, 202.2. FTIR u_max_ (KBr): 1202, 1235, 1427, 1586, 1658, 2964 cm^-1^. HRMS (ESI): *m/z* [M+H]^+^ calcd for C_14_H_15_NO_4_ 262.1074; found *m/z* 262.1070.

#### 1-(7-Fluoro-4-hydroxy-2-methylquinolin-3-yl)ethanone (3e)

Pale Brown solid (30 mg, 55%), mp.: > 240 ^o^C (decomposed). ^1^H NMR (400 MHz, DMSO-*d_6_* ): δ 2.38 (s, 3H), 2.47 (s, 3H), 7.24–7.18 (m, 2H), 8.13 (d, *J* = 2.0 Hz, 1H), 11.97 (br s, 1H). ^13^C NMR (100 MHz, DMSO-*d_6_*): δ 19.5, 32.4, 103.8, 113.2, 121.9, 129.0, 140.7, 152.5, 163.3, 165.8, 175.1, 201.9. FTIR u_max_ (KBr): 1161, 1353, 1515, 1636, 1674, 2874 cm^−1^. HRMS (ESI): *m/z* [M+H]^+^ calcd for C_12_H_10_FNO_2_ 220.0768; found *m/z* 220.0762.

#### 1-(6-Chloro-4-hydroxy-2-methylquinolin-3-yl)ethanone (3f)

Brown solid (23 mg, 39%), mp: > 250 ^o^C (decomposed). ^1^H NMR (400 MHz, DMSO-*d_6_* ): δ 2.38 (s, 3H), 2.47 (s, 3H), 7.62-7.53 (m, 2H), 7.99 (d, *J* = 2.4 Hz, 1H). ^13^C NMR (100 MHz, DMSO-*d_6_* ): δ 20.9, 32.5, 120.4, 122.7, 124.4, 127.6, 128.3, 132.0, 140.1, 154.0, 174.4, 202.0. FTIR u_max_ (KBr): 1259, 1509, 1685, 2905 cm^−1^. HRMS (ESI): *m/z* [M+H]^+^ calcd for C_12_H_10_ClNO_2_ 236.0473; found *m/z* 236.0475.

#### 1-(7-Chloro-4-hydroxy-2-methylquinolin-3-yl)ethanone (3g)

Pale yellow solid (30 mg, 51%), mp > 287 ^o^C (decomposed). ^1^H NMR (400 MHz, DMSO-*d_6_* ): δ 2.39 (s, 3H), 2.47 (s, 3H), 7.37 (dt, *J* = 8.5, 1.5 Hz, 1H), 7.53 (s, 1H), 8.08 (d, *J* = 8.8, 0.8 Hz, 1H), 12.04 (br s, 1H). ^13^C NMR (100 MHz, DMSO-*d_6_* ): δ 19.5, 32.4, 117.7, 121.2, 124.5, 124.8, 127.9, 137.3, 140.1, 152.5, 175.1, 201.8. FTIR u_max_ (KBr): 1350, 1505, 1686, 2911 cm^−1^. HRMS (ESI): *m/z* [M+H]^+^ calcd for C_12_H_10_ClNO_2_ 236.0473; found *m/z* 236.0463.

#### 1-(6-Bromo-4-hydroxy-2-methylquinolin-3-yl)ethanone (3h)

White solid (10 mg, 14%), mp > 299 ^o^C (decomposed). ^1^H NMR (400 MHz, DMSO-*d_6_* ): δ 2.39 (s, 3H), 2.47 (s, 3H), 7.49 (d, *J* = 9.2 Hz, 1H), 7.81 (dd, *J* = 8.6, 2.2 Hz, 1H), 8.16 (d, *J* = 2.4 Hz, 1H), 12.07 (br s, 1H). ^13^C NMR (100 MHz, DMSO-*d_6_* ): δ 19.5, 32.4, 117.1, 120.9, 121.1, 127.3, 127.7, 135.5, 138.2, 152.4, 174.4, 201.8. FTIR u_max_ (KBr): 1347, 1545, 2899 cm^−1^. HRMS (ESI): *m/z* [M+H]^+^ calcd for C_12_H_10_BrNO_2_ 279.9968; found *m/z* 279.9962.

#### (4-Hydroxy-6-iodo-2-phenylquinolin-3-yl)(phenyl)methanone (3i)

Brown solid (30 mg, 27%), mp.: > 301 ^o^C (decomposed). ^1^H NMR (400 MHz, DMSO-*d_6_* ): δ 7.42–7.40 (m, 7H), 7.54 (d, *J* = 8.0 Hz, 2H), 7.75 (d, *J* = 7.2 Hz, 2H), 8.01 (d, *J* = 8.4 Hz, 1H), 8.34 (s, 1H), 12.24 (s, 1H). ^13^C NMR (100 MHz, DMSO-*d_6_*): δ 89.2, 121.1, 121.7, 126.9, 129.0, 129.1, 129.4, 130.6, 133.6, 133.7, 133.8, 138.1, 139.6, 140.9, 150.2, 174.1, 195.8. FTIR u_max_ (KBr): 581, 1345, 1667, 2798 cm^−1^. HRMS (ESI): *m/z* [M+H]^+^ calcd for C_22_H_14_INO_2_ 452.0142; found *m/z* 452.0123.

#### 1-(4-Hydroxy-6-iodo-2-methylquinolin-3-yl)ethanone (3j)

Brown solid (36 mg, 44%), mp.: > 301 ^o^C (decomposed). ^1^H NMR (400 MHz, DMSO-*d_6_* ): δ 2.38 (s, 3H), 2.46 (s, 3H), 7.33 (d, *J* = 8.4 Hz 1H), 7.93 (dd, *J* = 8.8, 2.0 Hz, 1H), 8.35 (d, *J* = 2.0 Hz, 1H), 12.07 (brs, 1H). ^13^C NMR (100 MHz, DMSO-*d_6_* ): δ 19.6, 32.4, 89.2, 120.9, 121.0, 127.6, 134.0, 138.6, 140.8, 152.4, 174.3, 201.8. FTIR u_max_ (KBr): 1345, 1503, 1573, 1630, 2902 cm^−1^. HRMS (ESI): *m/z* [M+H]^+^ calcd for C_12_H_10_INO_2_ 327.9829; found *m/z* 327.9826.

#### 6,8-Dichloro-2-methylquinolin-4-ol (3kb)

^1^H NMR (400 MHz, DMSO-*d*_6_): δ 2.39 (s, 3H), 6.01 (s, 1H), 7.94 (s, 2H), 10.95 (s, 1H). ^13^C NMR (100 MHz, DMSO-*d*_6_): δ 120.3, 110.1, 123.1, 123.8, 127.1, 127.6, 131.8, 136.2, 152.1, 176.3. FTIR υmax (KBr): 529, 839, 1141, 1498, 1570, 1595, 1631, 2995 cm^−1^. HRMS (ESI): *m/z* [M+H]^+^ calcd for C_10_H_7_Cl_2_NO 227.9977 [M+H]^+^, found, *m/z* 227.9972.

#### 6,8-Dibromo-2-methylquinolin-4-ol (3lb)

^1^H NMR (400 MHz, DMSO-*d*_6_): δ 2.27 (s, 3H), 5.96 (s, 1H), 7.85 (s, 1H), 8.09 (s, 1H). ^13^C NMR (100 MHz, DMSO-*d*_6_): δ 67.8, 111.1, 126.9, 129.1, 132.0, 132.1, 133.0, 145.9, 159.9, 167.4. FTIR υmax (KBr): 838, 1122, 1439, 1564, 1626, 3198 cm^−1^. HRMS (ESI): *m/z* [M+H]^+^ calcd for C_10_H_7_Br_2_NO 315.8967 [M+H]^+^; found, *m/z* 315.8965.

#### 1.1. General method for the synthesis of 3-acylamino-4(3H) quinazolinones (6a–h)

*N*-(2-aminobenzoyl) benzotriazole compounds 1 (0.25 mmol) were refluxed with orthoesters 4 (0.5 mmol) and hydrazides 5 (0.5 mmol) in 2 mL of dioxane for 18–20 h. The reactions were controlled by thin layer chromatography (TLC). At the end of the reaction, the solvent was vaporised under reduced pressure. The obtained residue was purified using column chromatography in EtOAc/Hexane mixtures (1:2 or 1:3).

##### *N*-(4-Oxoquinazolin-3(4*H*)-yl)acetamide (6a)

Orange solid (32.3 mg, 64%); mp.: 199–201 ^o^C. ^1^H NMR (400 MHz, DMSO-*d_6_* ): δ 2.07 (s, 3H), 7.60–7.56 (m, 1H), 7.71 (d, *J* = 8 Hz, 1H), 7.89–7.85 (m, 1H), 8.16 (dd, *J* = 8.2 Hz, 1.4 Hz, 1H), 8.23 (s, 1H), 11.26 (s, 1H). ^13^C NMR (100 MHz, DMSO-*d_6_* ): δ 20.9, 122.4, 126.8, 127.9, 128.0, 135.4, 147.6, 149.4, 158.9, 169.9. FTIR u_max_ (KBr): 1473, 1502, 1667, 3270 cm^−1^. HRMS (ESI): *m/z* [M+H]^+^ calcd for C_10_H_9_N_3_O_2_ 204.0768; found *m/z* 204.0768.

##### *N*-(4-Oxoquinazolin-3(4*H*)-yl)benzamide (6b)

White solid (38.8mg, 59%); mp.: 188–189 °C. ^1^H NMR (400 MHz, DMSO-*d_6_* ): δ 7.68–7.55 (m, 4H), 7.76 (d, *J* = 8.4 Hz, 1H), 7.92–7.88 (m, 1H), 7.98–7.96 (m, 2H), 8.19 (dd, *J* = 7.6 Hz, 1.6 Hz, 1H), 8.43 (s, 1H), 11.86 (s, 1H). ^13^C NMR (100 MHz, DMSO-*d_6_* ): δ 122.4, 126.8, 128.0 128.1, 128.2, 129.2, 131.5, 133.3, 135.5, 147.7, 149.5, 159.0, 166.7. FTIR u_max_ (KBr): 1473, 1516, 1667, 3266 cm^−1^. HRMS (ESI): *m/z* [M+H]^+^ calcd for C_15_H_11_N_3_O_2_ 266.0924; found *m/z* 266.0914.

##### 4-Methoxy-*N*-(4-oxoquinazolin-3(4*H*)-yl)benzamide (6c)

Orange solid (25 mg, 35%); mp: 179–181 °C. ^1^H NMR (400 MHz, CDCl_3_) : δ 3.85 (s, 3H), 6.87 (t, *J* = 4.2 Hz, 2H), 7.54–7.50 (m, 1H), 7.80–7.75 (m, 2H), 7.87 (t, *J* = 4.2 Hz, 2H), 8.12 (d, *J* =1.2 Hz, 1H), 8.28 (d, *J* = 8 Hz, 1H), 9.61 (s, 1H). ^13^C NMR (100 MHz, CDCl_3_): δ 55.5, 114.0, 121.9, 122.5, 127.0, 127.6, 127.8, 129.4, 129.8, 135.0, 147.1, 160.0, 163.4, 167.0. FTIR u_max_ (KBr): 1175, 1475, 1606, 1666, 3254 cm^−1^. HRMS (ESI): *m/z* [M+H]^+^ calcd for C_16_H_13_N_3_O_3_ 296.1030; found *m/z* 296.1024.

##### *N*-(4-Oxo-2-phenylquinazolin-3(4H)-yl)benzamide (6d)

White solid (45 mg, 53%); mp.: 202–204 ^o^C. ^1^H NMR: (400 MHz, CDCl_3_): δ 7.30-7.25 (m, 2H), 7.43 (t, *J* = 6.2 Hz, 4H), 7.55–7.50 (m, 1H), 7.62 (d, *J* = 7.6 Hz, 2H), 7.76 (d, *J* = 3.2 Hz, 2H), 7.81 (d, *J* = 4 Hz, 2H), 8.29 (d, *J* = 8 Hz, 1H), 9.42 (s, 1H). ^13^C NMR (100 MHz, CDCl_3_): δ 121.2, 127.1, 127.8, 128.0, 128.2, 128.3, 128.9, 129.1, 130.1, 131.6, 133.1, 133.8, 135.8, 147.1, 156.7, 160.1, 165.8. FTIR u_max_ (KBr): 1567, 1602, 1719, 3158 cm^−1^. HRMS (ESI): *m/z* [M+H]^+^ calcd for C_21_H_15_N_3_O_2_ 342.1237; found *m/z* 342.1231.

##### 2,5-diphenyl-1,3,4-oxadiazole (6d′)

White solid (20 mg, 36%) mp.: 139–140 ^o^C. ^1^H NMR: (400 MHz, CDCl_3_): δ 7.55 (d, *J* = 5.6 Hz, 6H), 8.15 (t, *J* = 3.8 Hz, 4H). ^13^C NMR (100 MHz, CDCl_3_): δ 123.9, 126.9, 129.1, 131.7, 164.6. FTIR u_max_ (KBr): 1069, 1268, 1446, 1485, 1547, 1605 cm^−1^ HRMS (ESI): *m/z* [M+H]^+^ calcd for C_14_H_10_N_2_O 223.0866; found *m/z* 223.0864.

##### 4-Methyl-*N*-(4-oxoquinazolin-3(4H)-yl)benzamide (6e)

White solid (24 mg, 34%); mp.: 202–204 ^o^C. ^1^H NMR: (400 MHz, DMSO-*d*_6_): δ 2.39 (s, 3H), 7.38 (d, *J* = 8 Hz, 2H), 7.61 (t, *J* = 7.4 Hz, 1H), 7.76 (d, *J* = 8.4 Hz, 1H), 7.92-7.87 (m, 3H), 8.19 (s, *J* = 6.8 Hz, 1H), 8.40 (s, 1H), 11.77 (s, 1H). ^13^C NMR (100 MHz, DMSO-*d*_6_): δ21.5, 122.4, 126.8, 128.0, 128.1, 128.3, 128.7, 129.7, 135.5, 143.4, 147.7, 149.6, 159.1, 166.6. FTIR u_max_ (KBr): 1478, 1497, 1613, 1664, 3242 cm^−1^. HRMS (ESI): *m/z* [M+H]^+^ calcd for C_16_H_13_N_3_O_2_ 280.1081; found *m/z* 280.1071.

##### 2-(p-Tolyl)-1,3,4-oxadiazole (6e′)

Pale orange solid (31.1 mg, 78%); mp.: 85–86 ^o^C. ^1^H NMR: (400 MHz, CDCl_3_): δ 2.42 (s, 3H), 7.31 (d, *J* = 8.4 Hz, 2H), 7.96 (d, *J* = 8 Hz, 2H), 8.43 (s, 1H). ^13^C NMR (100 MHz, CDCl_3_): δ 21.6, 120.6, 127.0, 129.8, 142.6, 152.3, 164.9. FTIR u_max_ (KBr): 1067, 1102, 1497, 1611, 1927, 3126 cm^−1^. HRMS (ESI): *m/z* [M+H]^+^ calcd for C_9_H_8_N_2_O 161.0709; found *m/z* 161.0686.

##### *N*-(7-Fluoro-4-oxoquinazolin-3(4*H*)-yl)benzamide (6f)

White solid (46 mg, 65%); mp.: 189–191 ^o^C. ^1^H NMR: (400 MHz, DMSO-*d*_6_): δ 7.52-7.46 (m, 1H), 7.60–7.56 (m, 3H), 7.69–7.67 (m, 1H), 7.98 (d, *J* = 8 Hz, 2H), 8.26 (dd, *J* = 8.8 Hz, 6 Hz, 1H), 8.51 (s, 1H), 11.89 (s, 1H). ^13^C NMR (100 MHz, DMSO-*d*_6_): δ 113,5, 116.8, 119.4, 127.9, 128.2, 129.1, 130.1, 131.4, 133.3, 150.0, 150.9, 158.3, 165.1, 166.7, 167.6. FTIR u_max_ (KBr): 856, 1446, 1482, 1609, 1667, 1716, 3213 cm^−1^. HRMS (ESI): *m/z* [M+H]^+^ calcd for C_15_H_10_FN_3_O_2_ 284.0830; found *m/z* 284.0820.

##### Ethyl (4-oxoquinazolin-3(4*H*)-yl)carbamate (6g)

White solid (46.5 mg, 84%); mp.:179–181 ^o^C. ^1^H NMR: (400 MHz, DMSO-*d*_6_): δ 1.24 (t, *J* = 7.2 Hz, 3H), 4.19–4.13 (m, 2H), 7.61–7.57 (m, 1H), 7.72 (d, *J* = 7.6 Hz, 1H), 7.90-7.86 (m, 1H), 8.16 (dd, *J* = 8 Hz, 1.2 Hz, 1H), 8.35 (s, 1H), 10.67 (s, 1H). ^13^C NMR (100 MHz, DMSO-*d*_6_): δ 14.8, 62.3, 122.2, 126.8, 128.1, 128.2, 135.5, 147.6, 149.6, 156.4, 159.2. FTIR u_max_ (KBr): 1473, 1519, 1668, 1752, 2987, 3204 cm^−1^. HRMS (ESI): *m/z* [M+H]^+^ calcd for C_11_H_11_N_3_O_3_ 234.0873; found *m/z* 234.0868.

##### Ethyl (2-methyl-4-oxoquinazolin-3(4*H*)-yl)carbamate (6h)

White solid (45 mg, 73%); mp.: 130–132 ^o^C^1^H NMR: (400 MHz, DMSO-*d*_6_): δ 1.25 (t, *J* = 7 Hz, 3H), 2.41 (s, 3H), 4.19–4.13 (m, 2H), 7.51 (t, *J* = 7.6 Hz, 1H), 7.63 (d, *J* = 8.4 Hz, 1H), 7.85–7.81 (m, 1H), 8.08 (d, *J* = 7.2 Hz, 1H), 10.45 (s, 1H). ^13^C NMR (100 MHz, DMSO-*d*_6_): δ14.8, 21.6, 62.3, 120.9, 126.8, 127.3, 127.4, 135.5, 146.9, 156.1, 156.9, 159.7. FTIR u_max_ (KBr): 1471, 1610, 1663, 1754, 2990, 3217 cm^−1^. HRMS (ESI): *m/z* [M+H]^+^ calcd for C_12_H_13_N_3_O_3_ 248.1030; found *m/z* 248.1035.

## 4. Conclusion

A novel method has been developed for the synthesis of 3-Acyl-2-alkyl(aryl)-4-hydroxyquinolines and 3-acylamino-4(3*H*) quinazolinones. Quinoline derivatives were synthesized in one step from the reaction of *N*-(2-aminobenzoyl)benzotriazoles, which are easy-handle starting materials with diketones. Quinazolinone derivatives were obtained with *N*-(2-aminobenzoyl)benzotriazoles, orthoesters, and hydrazides as three-components with generally high yields. In comparison with the other methods in the literature, the reactions were carried out in the absence of any catalyst under mild reaction conditions in one-pot.

## Supplemental information

Figure S1 ^1^H Spectrum of 1-(4-Hydroxy-2-methylquinolin-3-yl)ethanone 3a.

Figure S2 ^13^C Spectrum of 1-(4-Hydroxy-2-methylquinolin-3-yl)ethanone 3a.

Figure S3 ^1^H Spectrum of (4-Hydroxy-6-methyl-2-phenylquinolin-3-yl)(phenyl)methanone (3b).

Figure S4 ^13^C Spectrum of (4-Hydroxy-6-methyl-2-phenylquinolin-3-yl)(phenyl)methanone (3b)

Figure S5 ^1^H Spectrum of (4-Hydroxy-8-methyl-2-phenylquinolin-3-yl)(phenyl)methanone (3c).

Figure S6 ^13^C Spectrum of (4-Hydroxy-8-methyl-2-phenylquinolin-3-yl)(phenyl)methanone (3c).

Figure S7 ^1^H Spectrum of 1-(4-Hydroxy-6,7-dimethoxy-2-methylquinolin-3-yl)ethanone (3d).

Figure S8 ^13^C Spectrum of 1-(4-Hydroxy-6,7-dimethoxy-2-methylquinolin-3-yl)ethanone (3d).

Figure S9 ^1^H Spectrum of 1-(7-Fluoro-4-hydroxy-2-methylquinolin-3-yl)ethanone (3e).

Figure S10 ^13^C Spectrum of 1-(7-Fluoro-4-hydroxy-2-methylquinolin-3-yl)ethanone (3e).

Figure S11 ^1^H Spectrum of 1-(6-Chloro-4-hydroxy-2-methylquinolin-3-yl)ethanone (3f).

Figure S12 ^13^C Spectrum of 1-(6-Chloro-4-hydroxy-2-methylquinolin-3-yl)ethanone (3f).

Figure S13 ^1^H Spectrum of 1-(7-Chloro-4-hydroxy-2-methylquinolin-3-yl)ethanone (3g).

Figure S14 ^13^C Spectrum of 1-(7-Chloro-4-hydroxy-2-methylquinolin-3-yl)ethanone (3g).

Figure S15 ^1^H Spectrum of 1-(6-Bromo-4-hydroxy-2-methylquinolin-3-yl)ethanone (3h).

Figure S16 ^13^C Spectrum of 1-(6-Bromo-4-hydroxy-2-methylquinolin-3-yl)ethanone (3h).

Figure S17 ^1^H Spectrum of (4-Hydroxy-6-iodo-2-phenylquinolin-3-yl)(phenyl)methanone (3i).

Figure S18 ^13^C Spectrum of (4-Hydroxy-6-iodo-2-phenylquinolin-3-yl)(phenyl)methanone (3i).

Figure S19 ^1^H Spectrum of 1-(4-Hydroxy-6-iodo-2-methylquinolin-3-yl)ethanone (3j).

Figure S20 ^13^C Spectrum of 1-(4-Hydroxy-6-iodo-2-methylquinolin-3-yl)ethanone (3j).

Figure S21 ^1^H Spectrum of *N*-(4-Oxoquinazolin-3(4*H*)-yl)acetamide (6a).

Figure S22 ^13^C Spectrum of *N*-(4-Oxoquinazolin-3(4*H*)-yl)acetamide (6a).

Figure S23 ^1^H Spectrum of *N*-(4-Oxoquinazolin-3(4*H*)-yl)benzamide (6b).

Figure S24 ^13^C Spectrum of *N*-(4-Oxoquinazolin-3(4*H*)-yl)benzamide (6b).

Figure S25 ^1^H Spectrum of 4-Methoxy-*N*-(4-oxoquinazolin-3(4*H*)-yl)benzamide (6c).

Figure S26 ^13^C Spectrum of 4-Methoxy-*N*-(4-oxoquinazolin-3(4*H*)-yl)benzamide (6c).

Figure S27 ^1^H Spectrum of *N*-(4-Oxo-2-phenylquinazolin-3(4*H*)-yl)benzamide (6d).

Figure S28 ^13^C Spectrum of *N*-(4-Oxo-2-phenylquinazolin-3(4*H*)-yl)benzamide (6d).

Figure S29 ^1^H Spectrum of 4-Methyl-*N*-(4-oxoquinazolin-3(4*H*)-yl)benzamide (6e).

Figure S30 ^13^C Spectrum of 4-Methyl-*N*-(4-oxoquinazolin-3(4*H*)-yl)benzamide (6e).

Figure S31 ^1^H Spectrum of *N*-(7-Fluoro-4-oxoquinazolin-3(4*H*)-yl)benzamide (6f).

Figure S32 ^1^H Spectrum of *N*-(7-Fluoro-4-oxoquinazolin-3(4*H*)-yl)benzamide (6f).

Figure S33 ^1^H Spectrum of Ethyl (4-oxoquinazolin-3(4*H*)-yl)carbamate (6g).

Figure S34 ^13^C Spectrum of Ethyl (4-oxoquinazolin-3(4*H*)-yl)carbamate (6g).

Figure S35 ^1^H Spectrum of Ethyl (2-methyl-4-oxoquinazolin-3(4*H*)-yl)carbamate (6h).

Figure S36 ^13^C Spectrum of Ethyl (2-methyl-4-oxoquinazolin-3(4*H*)-yl)carbamate (6h).

Figure S37 ^1^H Spectrum of 6,8-dichloro-2-methylquinolin-4-ol (3kb).

Figure S38 ^13^C Spectrum of 6,8-dichloro-2-methylquinolin-4-ol (3kb).

Figure S39 ^1^H Spectrum of 6,8-dibromo-2-methylquinolin-4-ol (3lb).

Figure S40 ^13^C Spectrum of 6,8-dibromo-2-methylquinolin-4-ol (3lb).

Figure S41 ^1^H Spectrum of 2,5-diphenyl-1,3,4-oxadiazole (6d′).

Figure S42 ^13^C Spectrum of 2,5-diphenyl-1,3,4-oxadiazole (6d′).

Figure S43 ^1^H Spectrum of 2-(*p*-Tolyl)-1,3,4-oxadiazole (6e′).

Figure S44 ^13^C Spectrum of 2-(*p*-Tolyl)-1,3,4-oxadiazole (6e′).

Figure S45 HRMS Spectrum of 1-(4-Hydroxy-2-methylquinolin-3-yl)ethanone (3a).

Figure S46 HRMS Spectrum of (4-Hydroxy-6-methyl-2-phenylquinolin-3-yl)(phenyl)methanone (3b).

Figure S47 HRMS Spectrum of (4-Hydroxy-8-methyl-2-phenylquinolin-3-yl)(phenyl)methanone (3c).

Figure S48 HRMS Spectrum of 1-(4-Hydroxy-6,7-dimethoxy-2-methylquinolin-3-yl)ethanone (3d).

Figure S49 HRMS Spectrum of 1-(7-Fluoro-4-hydroxy-2-methylquinolin-3-yl)ethanone (3e).

Figure S50 HRMS Spectrum of 1-(6-Chloro-4-hydroxy-2-methylquinolin-3-yl)ethanone (3f).

Figure S51 HRMS Spectrum of 1-(7-Chloro-4-hydroxy-2-methylquinolin-3-yl)ethanone (3g).

Figure S52 HRMS Spectrum of 1-(6-Bromo-4-hydroxy-2-methylquinolin-3-yl)ethanone (3h).

Figure S53 HRMS Spectrum of (4-Hydroxy-6-iodo-2-phenylquinolin-3-yl)(phenyl)methanone (3i).

Figure S54 HRMS Spectrum of 1-(4-Hydroxy-6-iodo-2-methylquinolin-3-yl)ethanone (3j).

Figure S55 HRMS Spectrum *of N*-(4-Oxoquinazolin-3(4*H*)-yl)acetamide (6a).

Figure S56 HRMS Spectrum of *N*-(4-Oxoquinazolin-3(4*H*)-yl)benzamide (6b).

Figure S57 HRMS Spectrum of 4-Methoxy-*N*-(4-oxoquinazolin-3(4*H*)-yl)benzamide (6c).

Figure S58 HRMS Spectrum of *N*-(4-Oxo-2-phenylquinazolin-3(4*H*)-yl)benzamide (6d).

Figure S59 HRMS Spectrum of 4-Methyl-*N*-(4-oxoquinazolin-3(4*H*)-yl)benzamide (6e).

Figure S60 HRMS Spectrum of *N*-(7-Fluoro-4-oxoquinazolin-3(4*H*)-yl)benzamide (6f).

Figure S61 HRMS Spectrum of Ethyl (4-oxoquinazolin-3(4*H*)-yl)carbamate (6g).

Figure S62 HRMS Spectrum of Ethyl (2-methyl-4-oxoquinazolin-3(4*H*)-yl)carbamate (6h).

Figure S63 HRMS Spectrum of 6,8-dichloro-2-methylquinolin-4-ol (3kb).

Figure S64 HRMS Spectrum of 6,8-dibromo-2-methylquinolin-4-ol (3lb).

Figure S65 HRMS Spectrum of 2,5-diphenyl-1,3,4-oxadiazole (6d′).

Figure S66 HRMS Spectrum of 2-(*p*-Tolyl)-1,3,4-oxadiazole (6e′).

## Figures and Tables

**Table 1:** The effect of temperature and solvent on the model reaction.

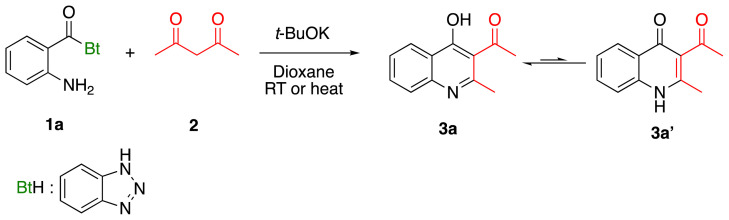		

Solvent	Reaction conditions	% Yield (3a)
THF	R.T	24
THF	Reflux	39
DMF	R.T	44
DMF	Reflux	49
Dioxane	Reflux	59

**Table 2:** 3-Acyl-2-alkyl(aryl)-4-hydroxyquinolines 3a–l.

Product	Structure	Yield % (Lit.)	Product	Structure	Yield % (Lit.)
**3a**	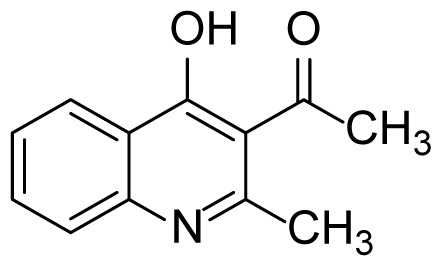	59 (51 ^29^)	**3g**	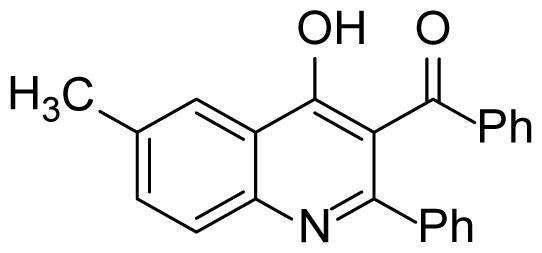	51 (39 ^6^)
**3b**	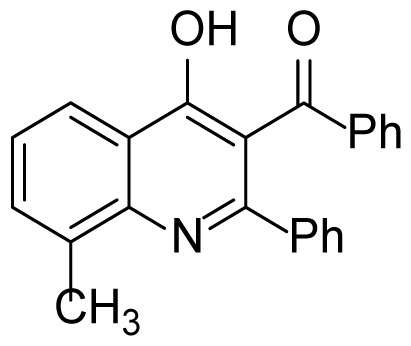	26 (88 ^2^)	**3h**	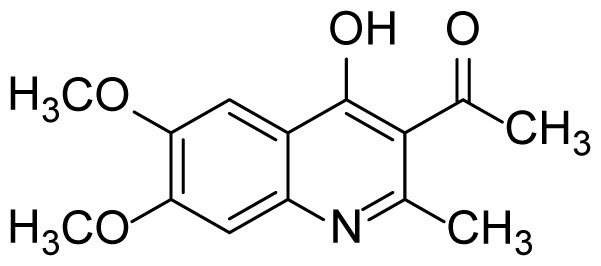	14 -
**3c**	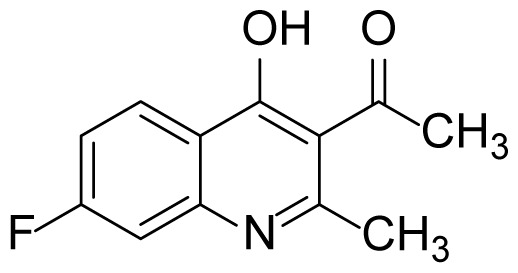	22 (76 ^30^)	**3i**	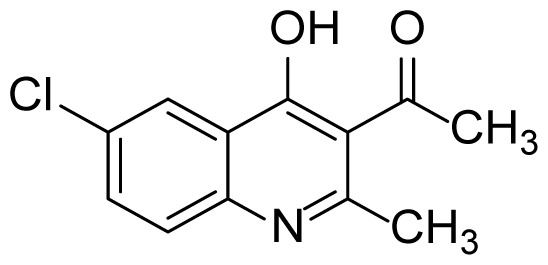	27 -
**3d**	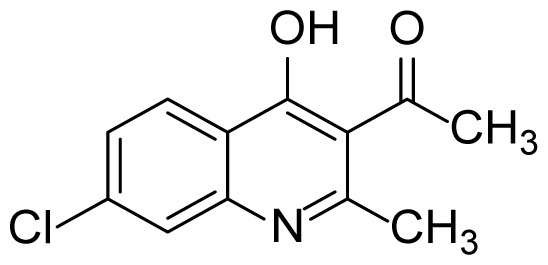	40 -	**3j**	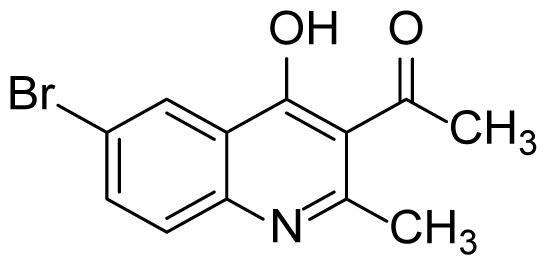	44 (89 ^31^)
**3e**	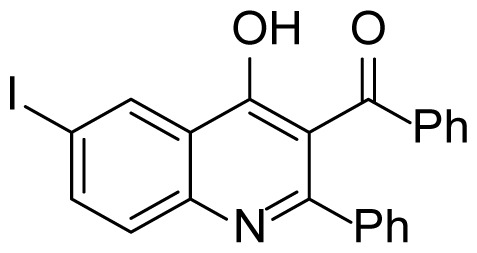	55 -	**3k**	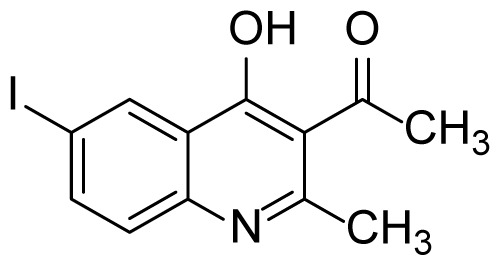	0 -
**3f**	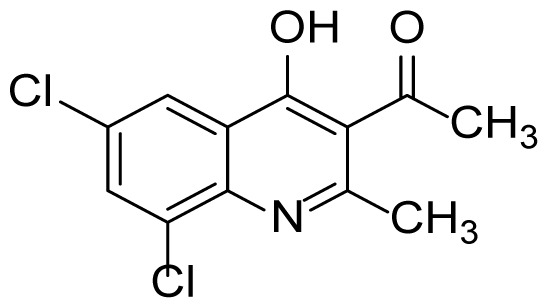	40 -	**3l**	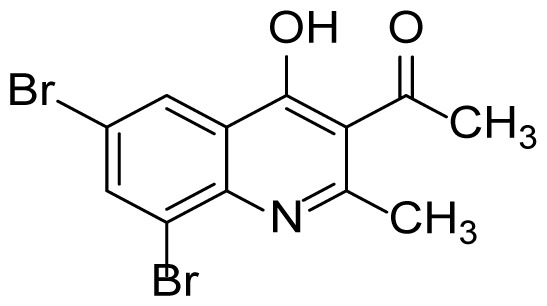	0 -

**Table 3:** 3-acylamino-4(3*H*) quinazolinones (6a–6h).

Product	Structure	Yield %(Lit.)	Product	Structure	Yield % (Lit.)
**6a**	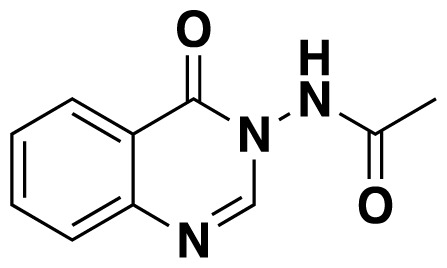	64 (65 ^32^)	**6e**	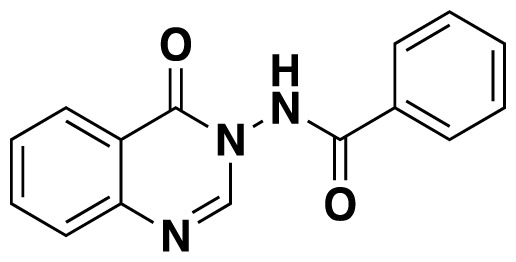	34 -
**6b**	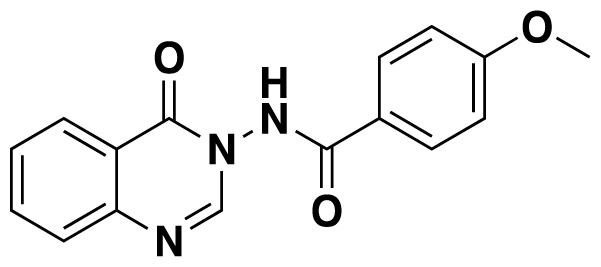	59 (52 ^33^)	**6f**	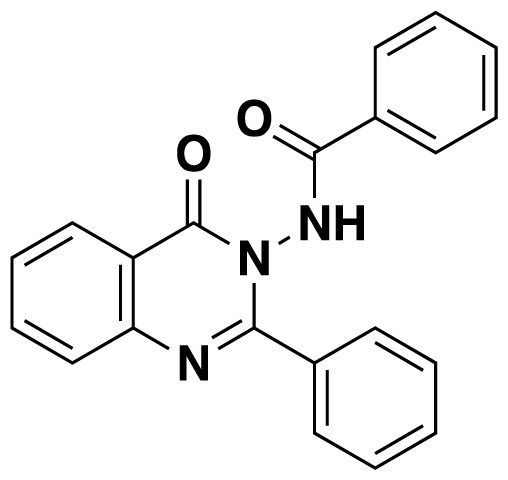	65 -
**6c**	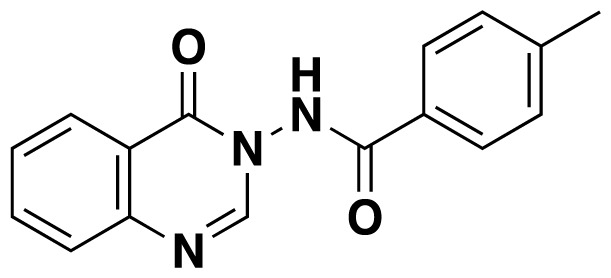	35 -	**6g**	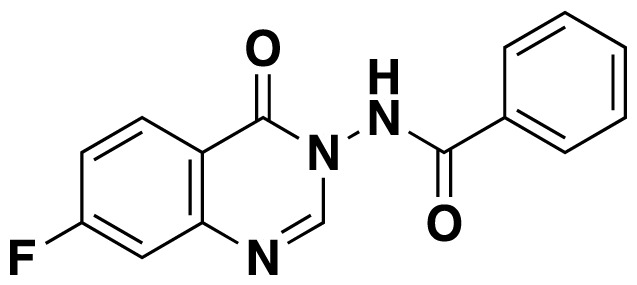	84 -
**6d**	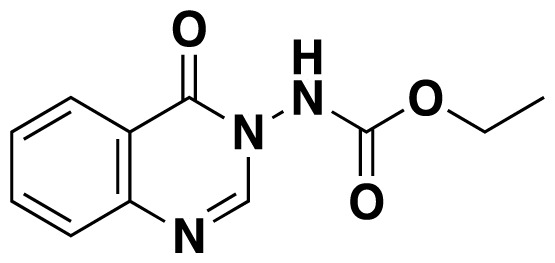	53 (50) [39]	**6h**	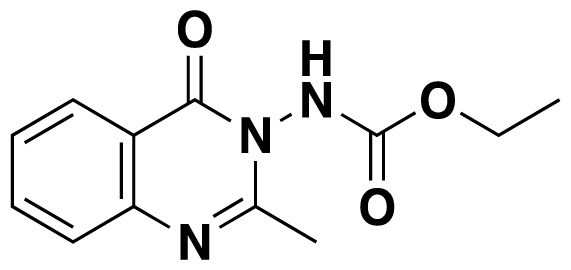	73 (88) [40]
