# Supplementary figures and images for: N-(2-Aminobenzoyl)benzotriazole Mediated Synthesis of 3-Acyl-2-alkyl(aryl)-4-hydroxyquinolines and 3-Acylamino-4(3H) quinazolinones
Source: Turk J Chem. 2023 Oct 16;48(1):97–107. doi: 10.55730/1300-0527.3642 (PMC10965190; doi:10.55730/1300-0527.3642)

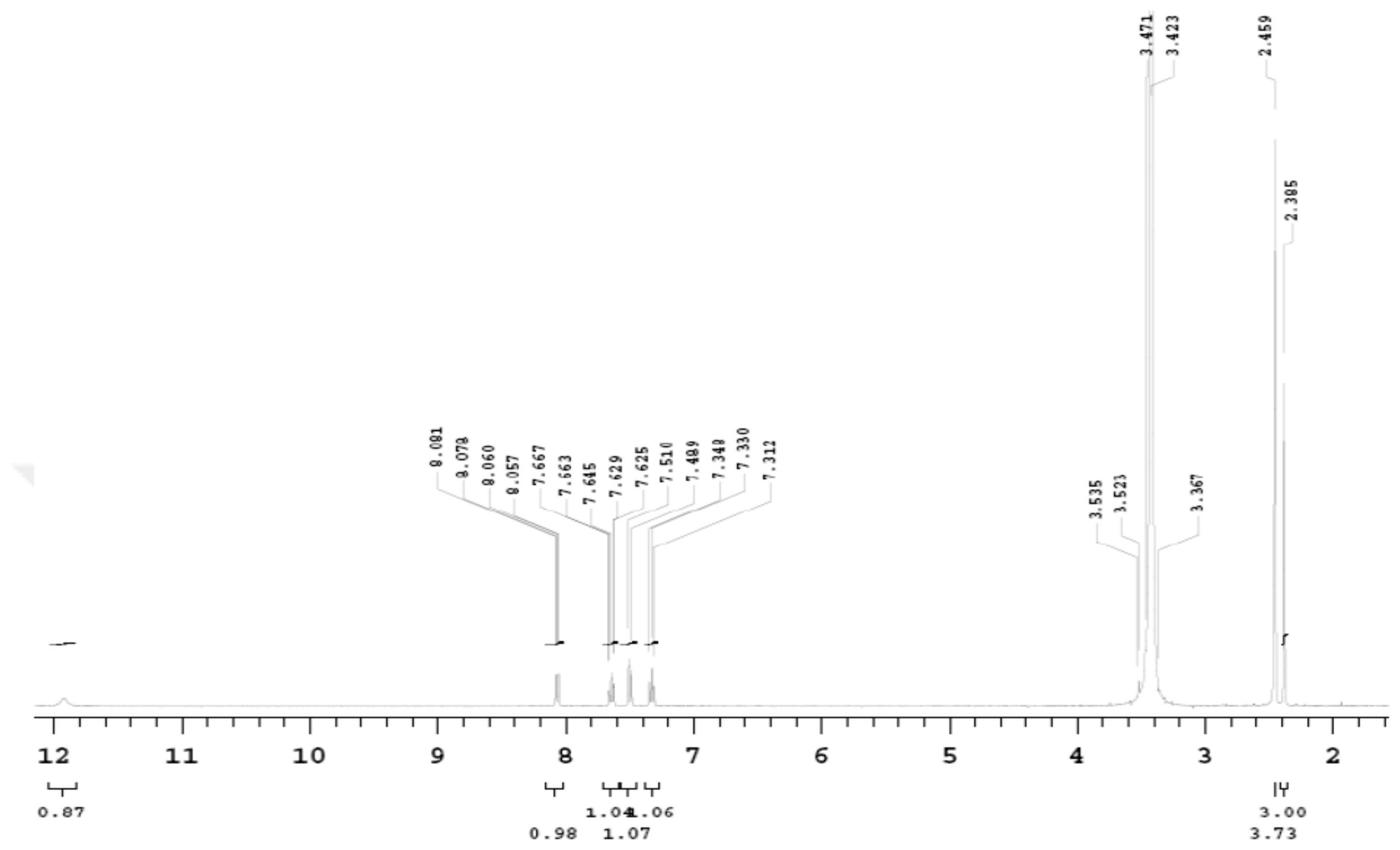

Supplement: Figure S1 — 1H Spectrum of 1-(4-Hydroxy-2-methylquinolin-3-yl)ethanone 3a. [file tjc-48-01-0097s1.tif]

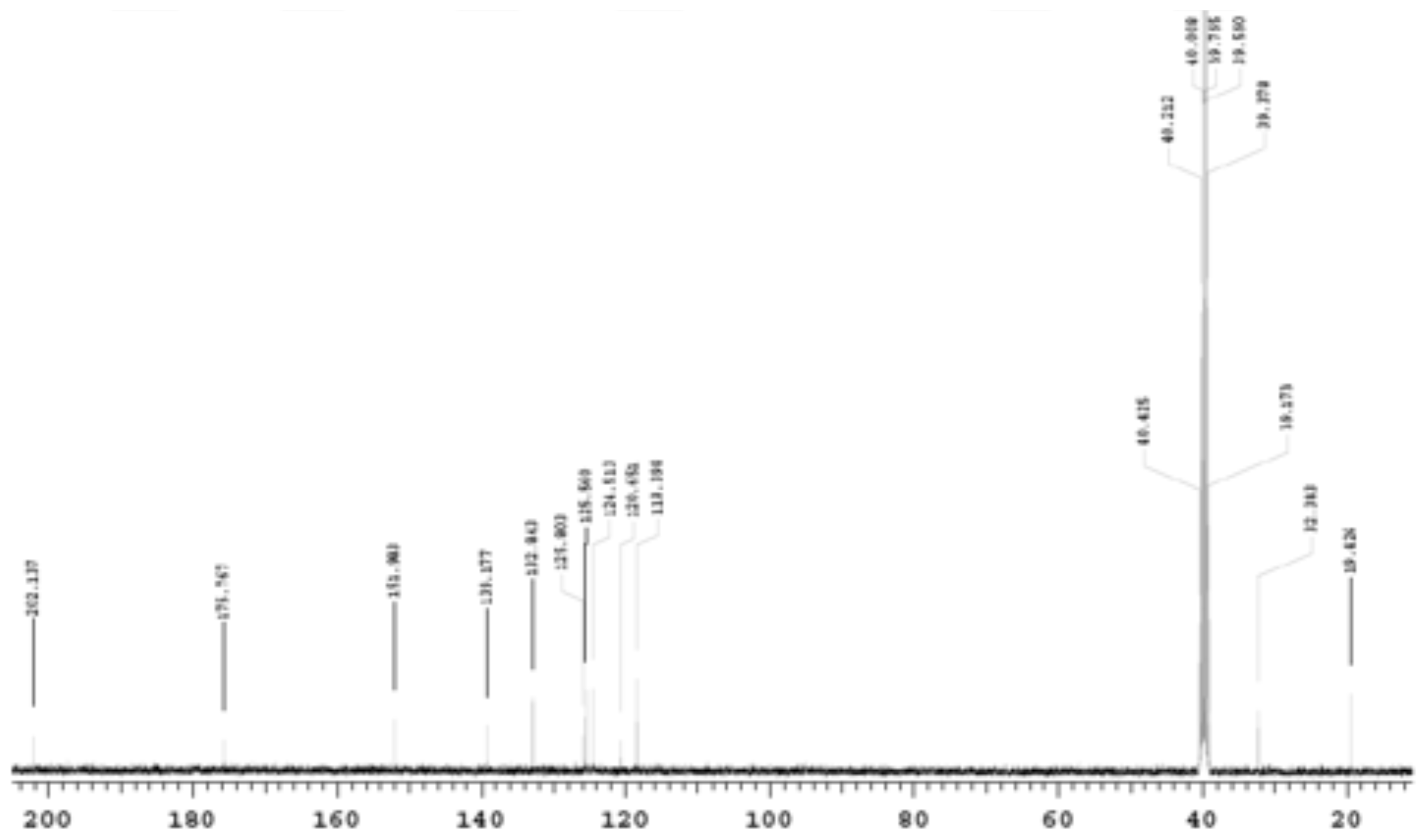

Supplement: Figure S2 — 13C Spectrum of 1-(4-Hydroxy-2-methylquinolin-3-yl)ethanone 3a. [file tjc-48-01-0097s2.tif]

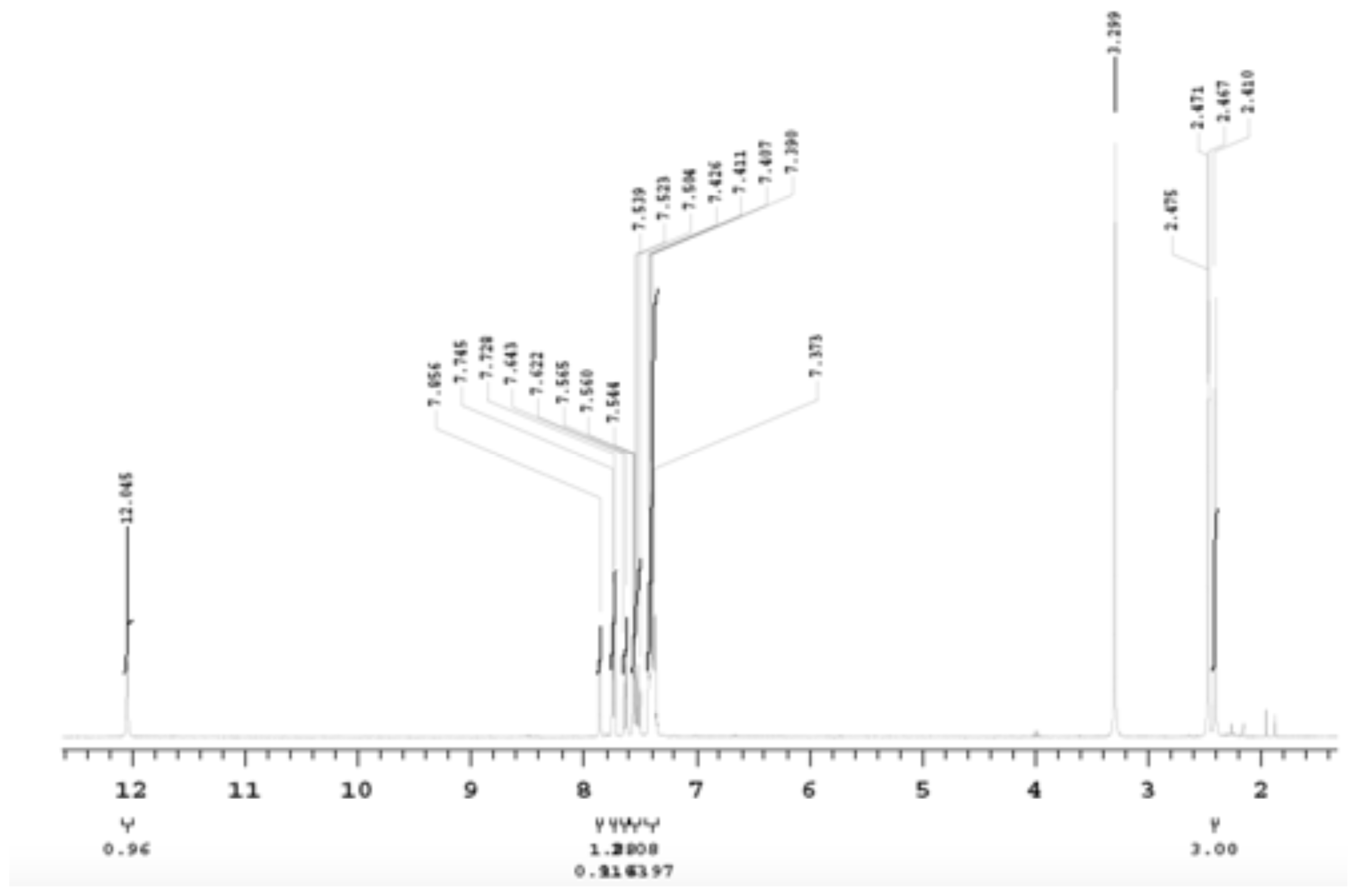

Supplement: Figure S3 — 1H Spectrum of (4-Hydroxy-6-methyl-2-phenylquinolin-3-yl)(phenyl)methanone (3b). [file tjc-48-01-0097s3.tif]

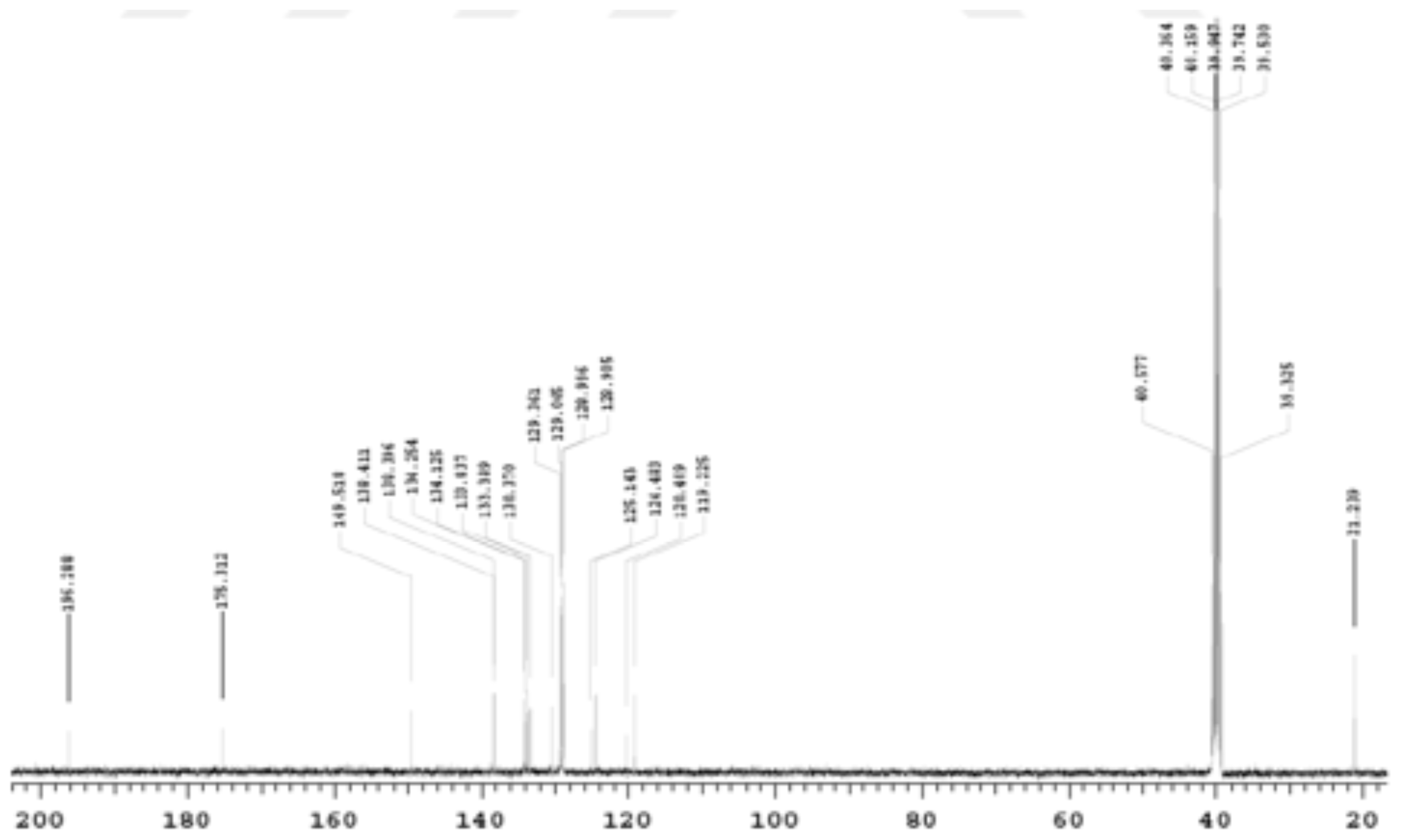

Supplement: Figure S4 — 13C Spectrum of (4-Hydroxy-6-methyl-2-phenylquinolin-3-yl)(phenyl)methanone (3b) [file tjc-48-01-0097s4.tif]

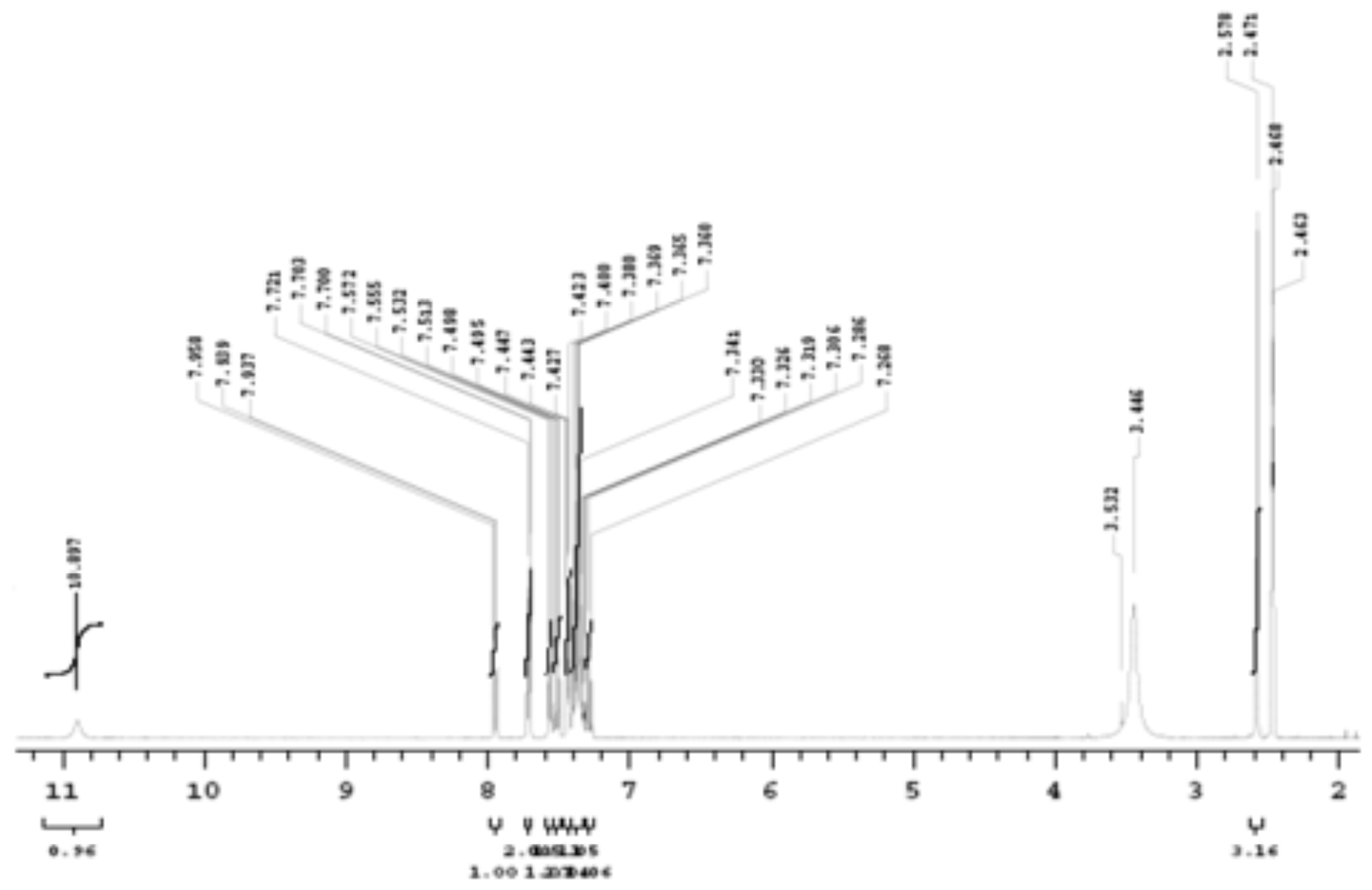

Supplement: Figure S5 — 1H Spectrum of (4-Hydroxy-8-methyl-2-phenylquinolin-3-yl)(phenyl)methanone (3c). [file tjc-48-01-0097s5.tif]

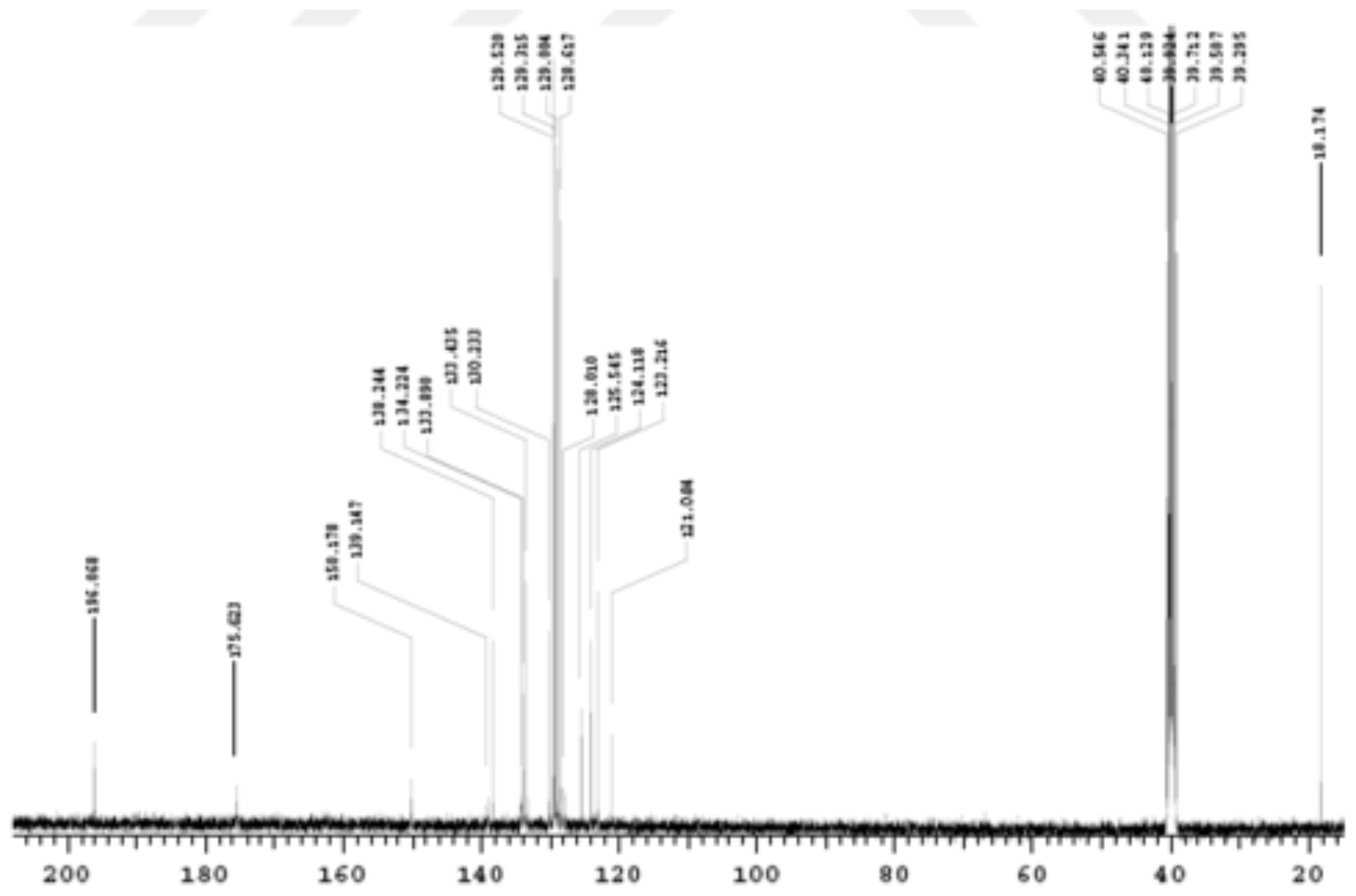

Supplement: Figure S6 — 13C Spectrum of (4-Hydroxy-8-methyl-2-phenylquinolin-3-yl)(phenyl)methanone (3c). [file tjc-48-01-0097s6.tif]

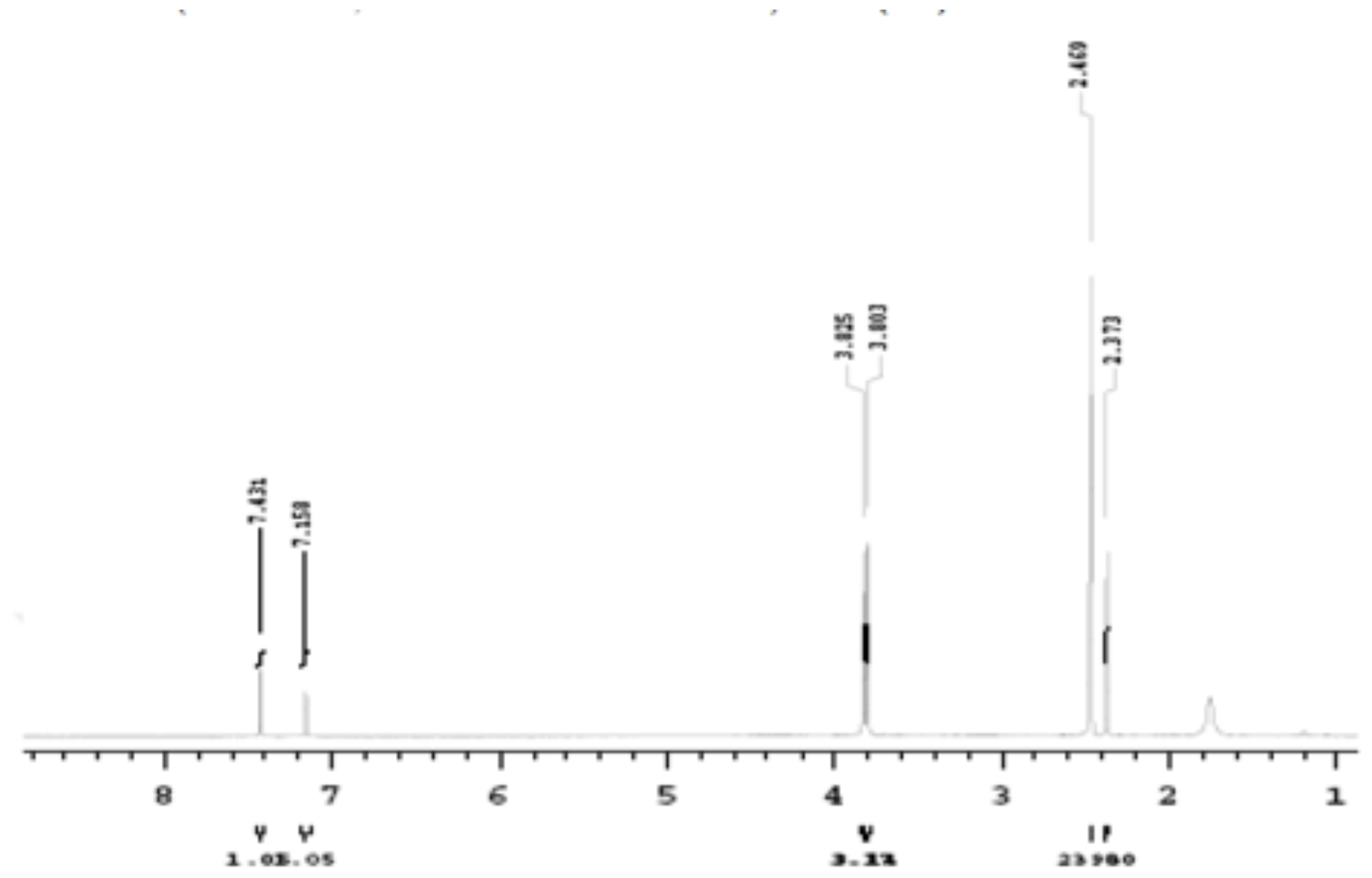

Supplement: Figure S7 — 1H Spectrum of 1-(4-Hydroxy-6,7-dimethoxy-2-methylquinolin-3-yl)ethanone (3d). [file tjc-48-01-0097s7.tif]

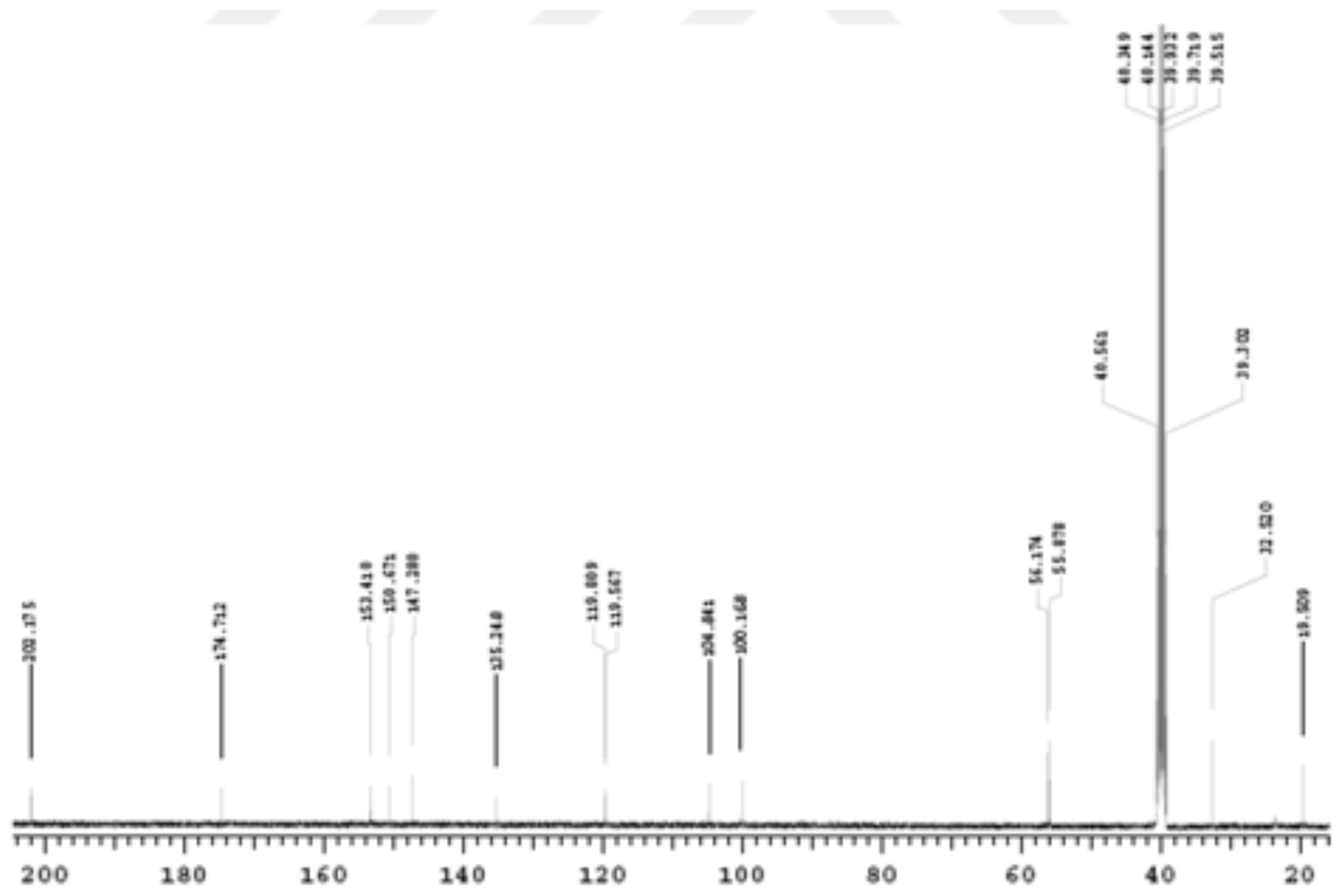

Supplement: Figure S8 — 13C Spectrum of 1-(4-Hydroxy-6,7-dimethoxy-2-methylquinolin-3-yl)ethanone (3d). [file tjc-48-01-0097s8.tif]

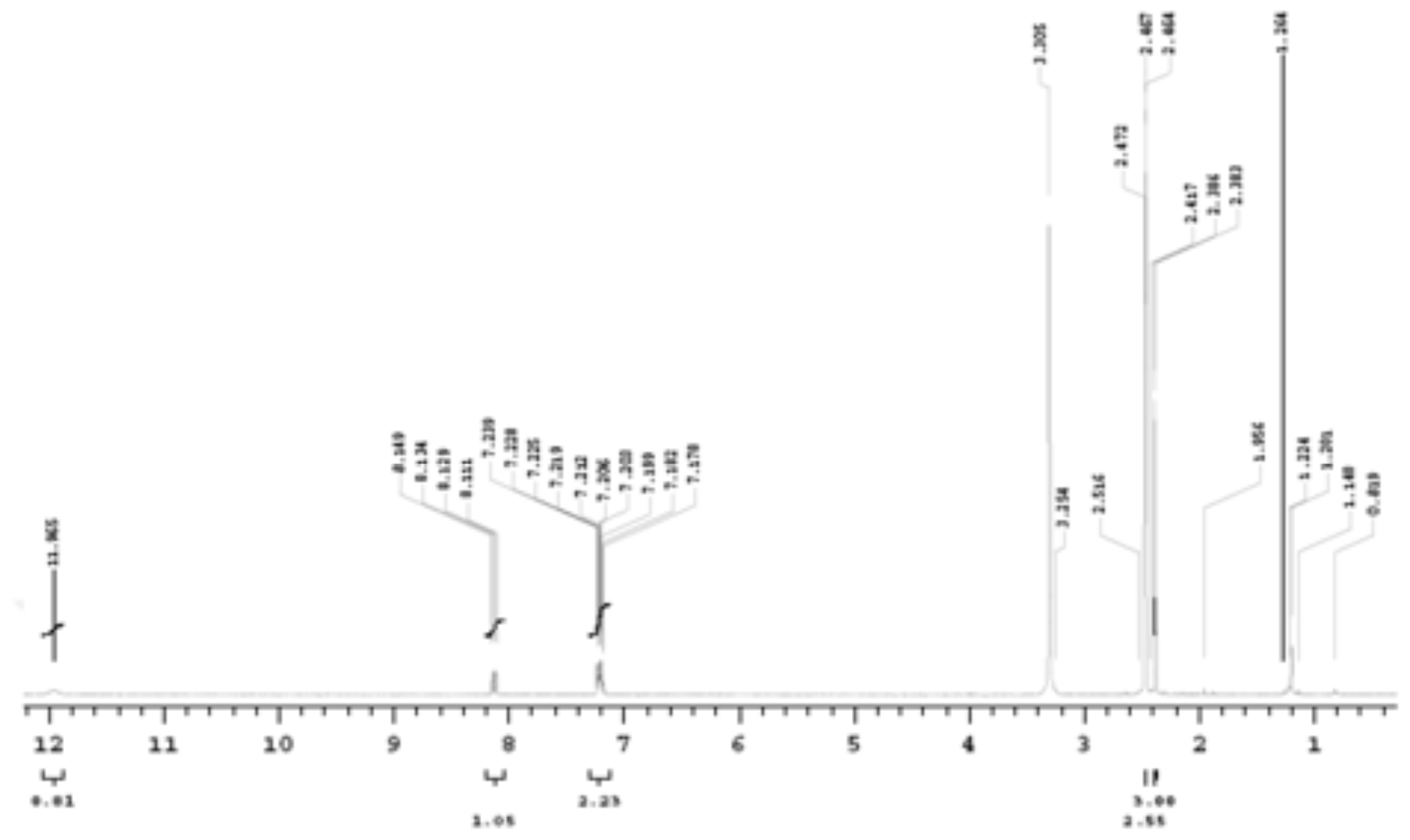

Supplement: Figure S9 — 1H Spectrum of 1-(7-Fluoro-4-hydroxy-2-methylquinolin-3-yl)ethanone (3e). [file tjc-48-01-0097s9.tif]

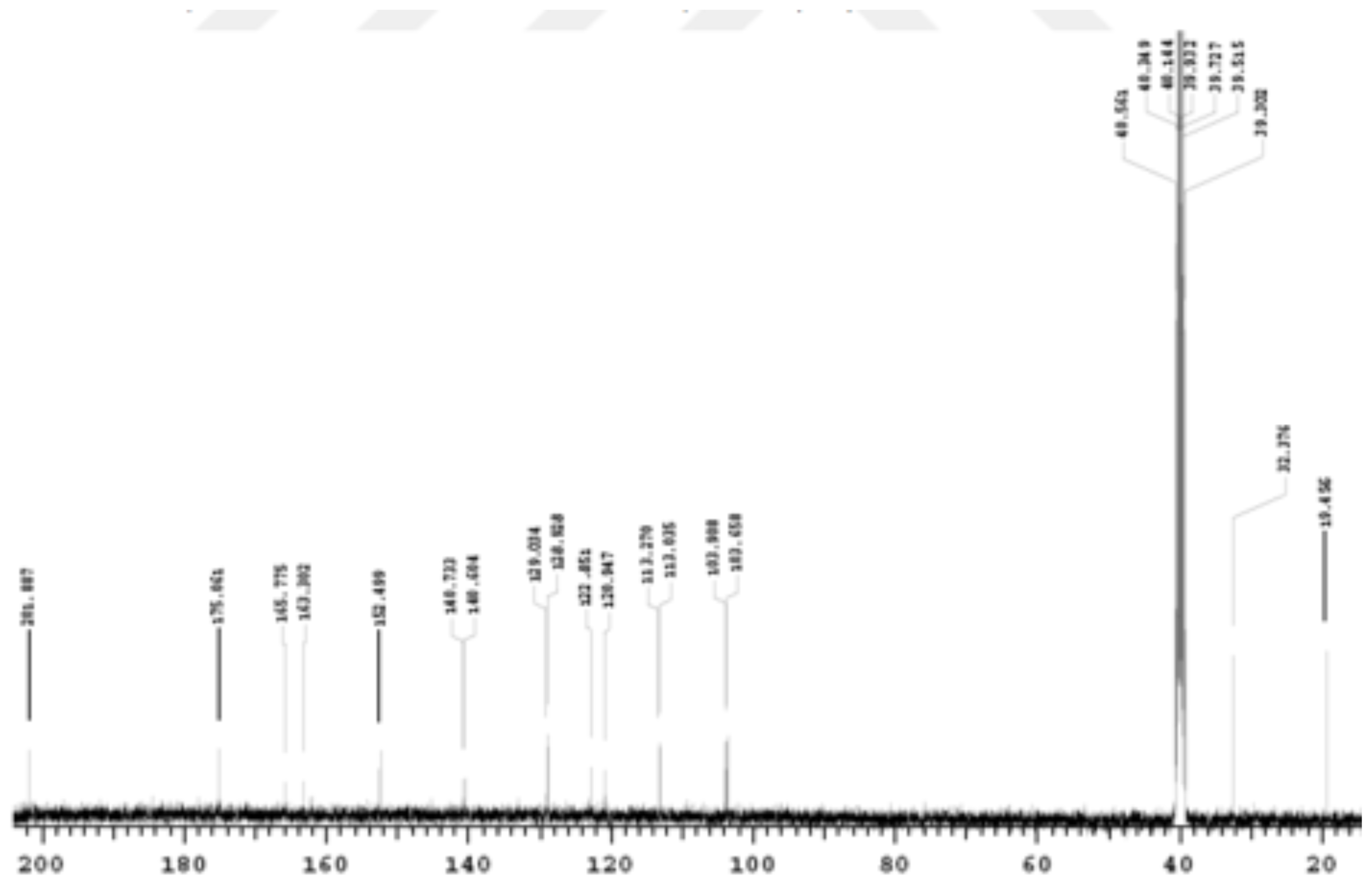

Supplement: Figure S10 — 13C Spectrum of 1-(7-Fluoro-4-hydroxy-2-methylquinolin-3-yl)ethanone (3e). [file tjc-48-01-0097s10.tif]

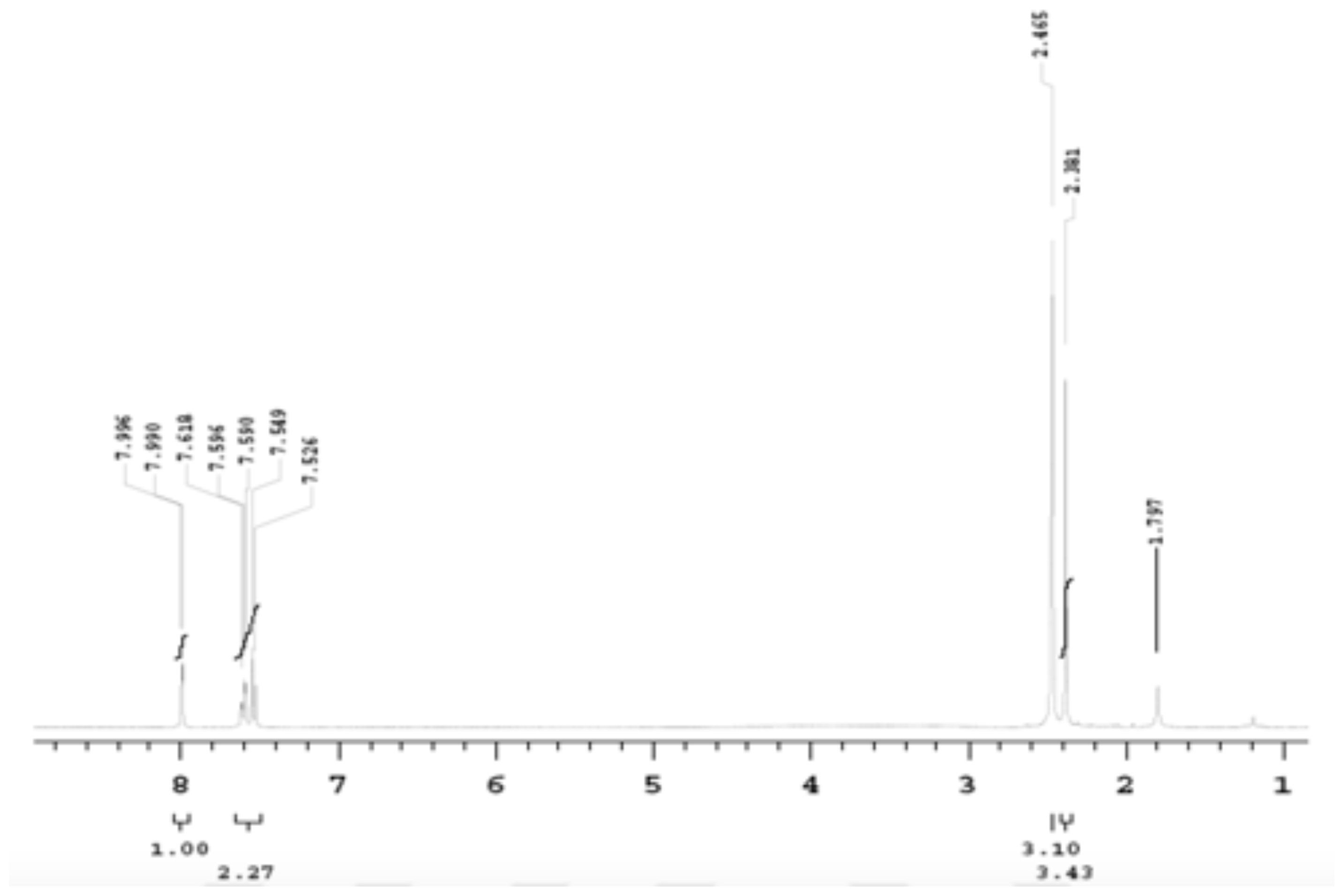

Supplement: Figure S11 — 1H Spectrum of 1-(6-Chloro-4-hydroxy-2-methylquinolin-3-yl)ethanone (3f). [file tjc-48-01-0097s11.tif]

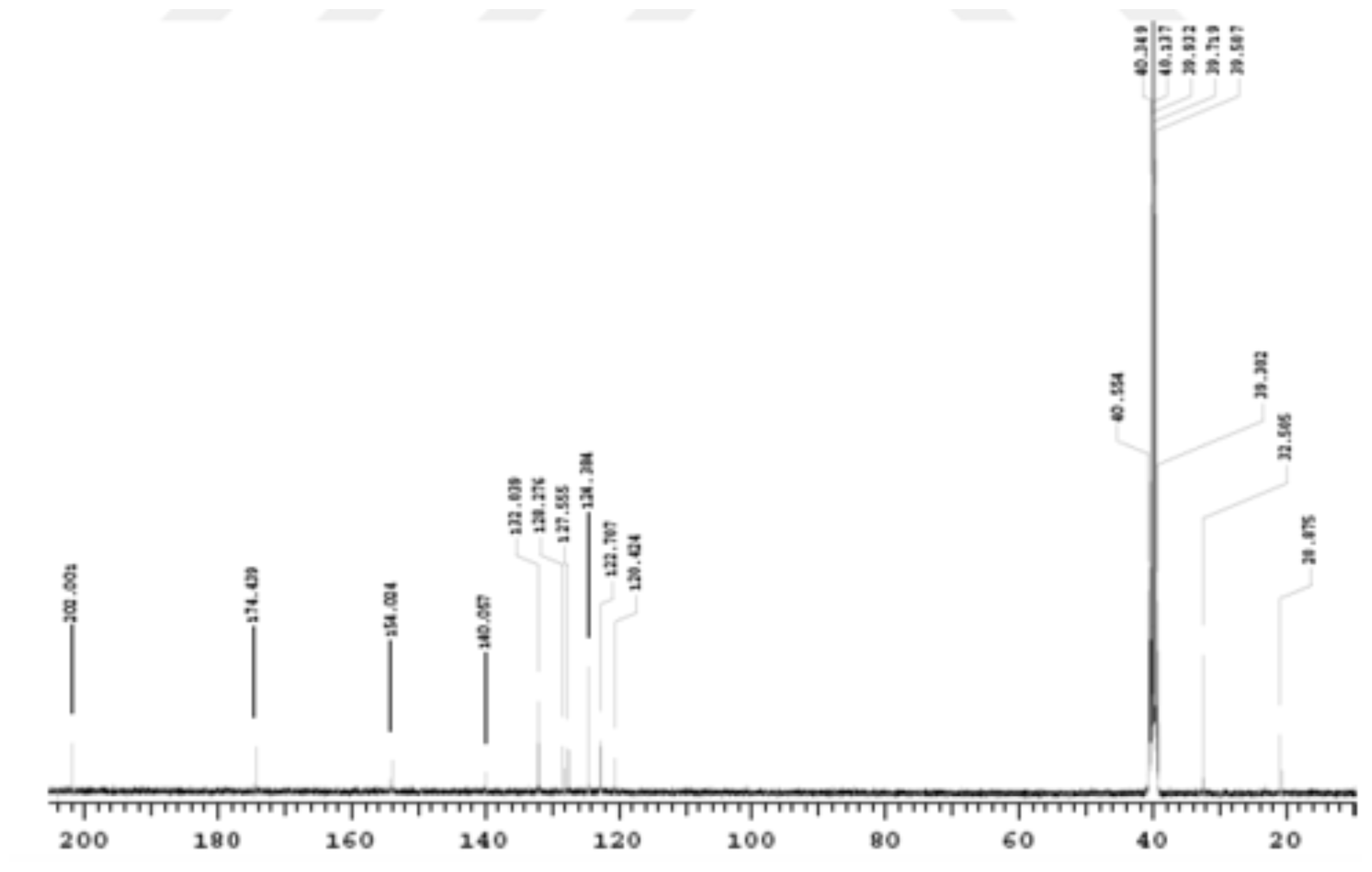

Supplement: Figure S12 — 13C Spectrum of 1-(6-Chloro-4-hydroxy-2-methylquinolin-3-yl)ethanone (3f). [file tjc-48-01-0097s12.tif]

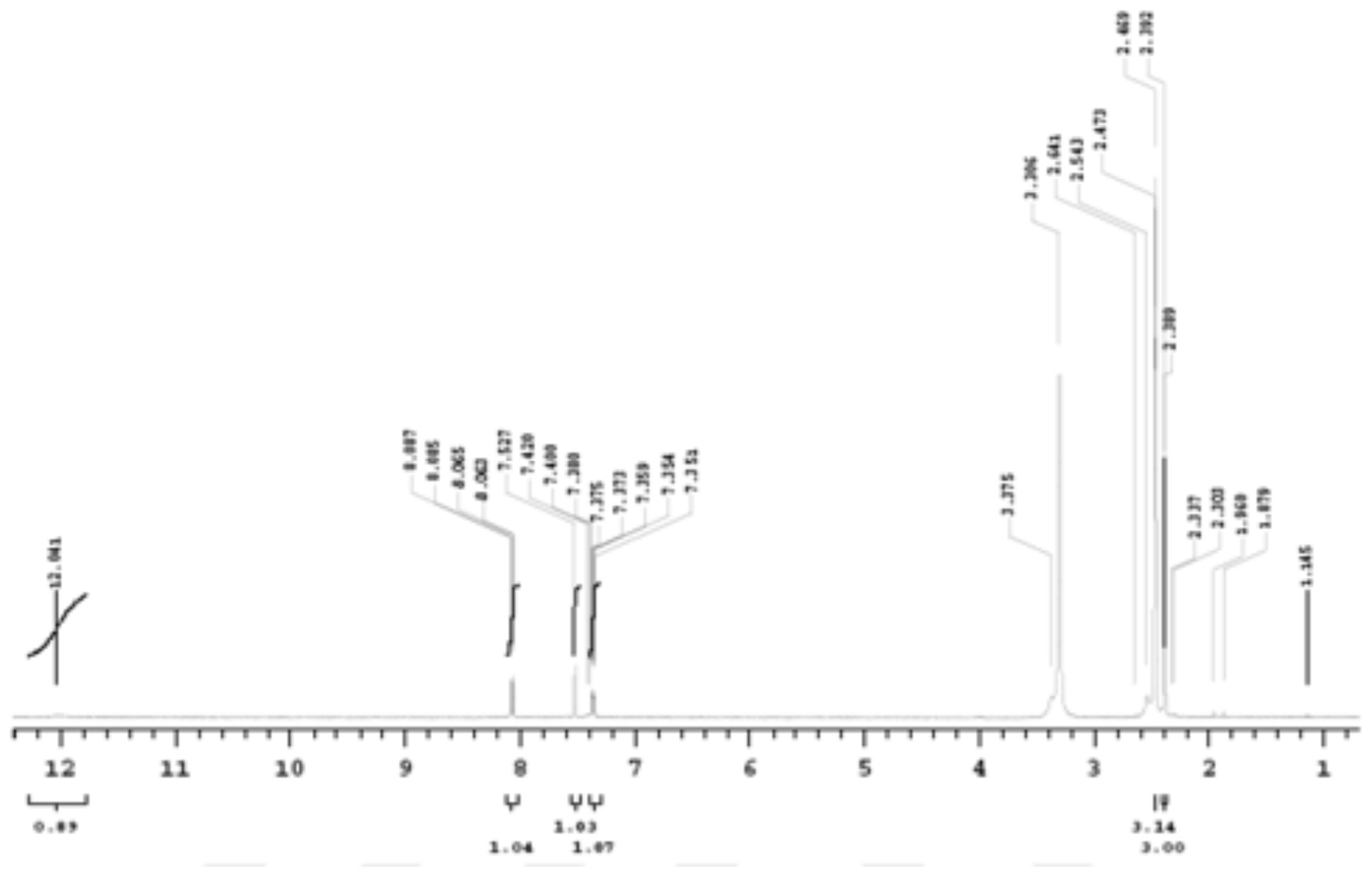

Supplement: Figure S13 — 1H Spectrum of 1-(7-Chloro-4-hydroxy-2-methylquinolin-3-yl)ethanone (3g). [file tjc-48-01-0097s13.tif]

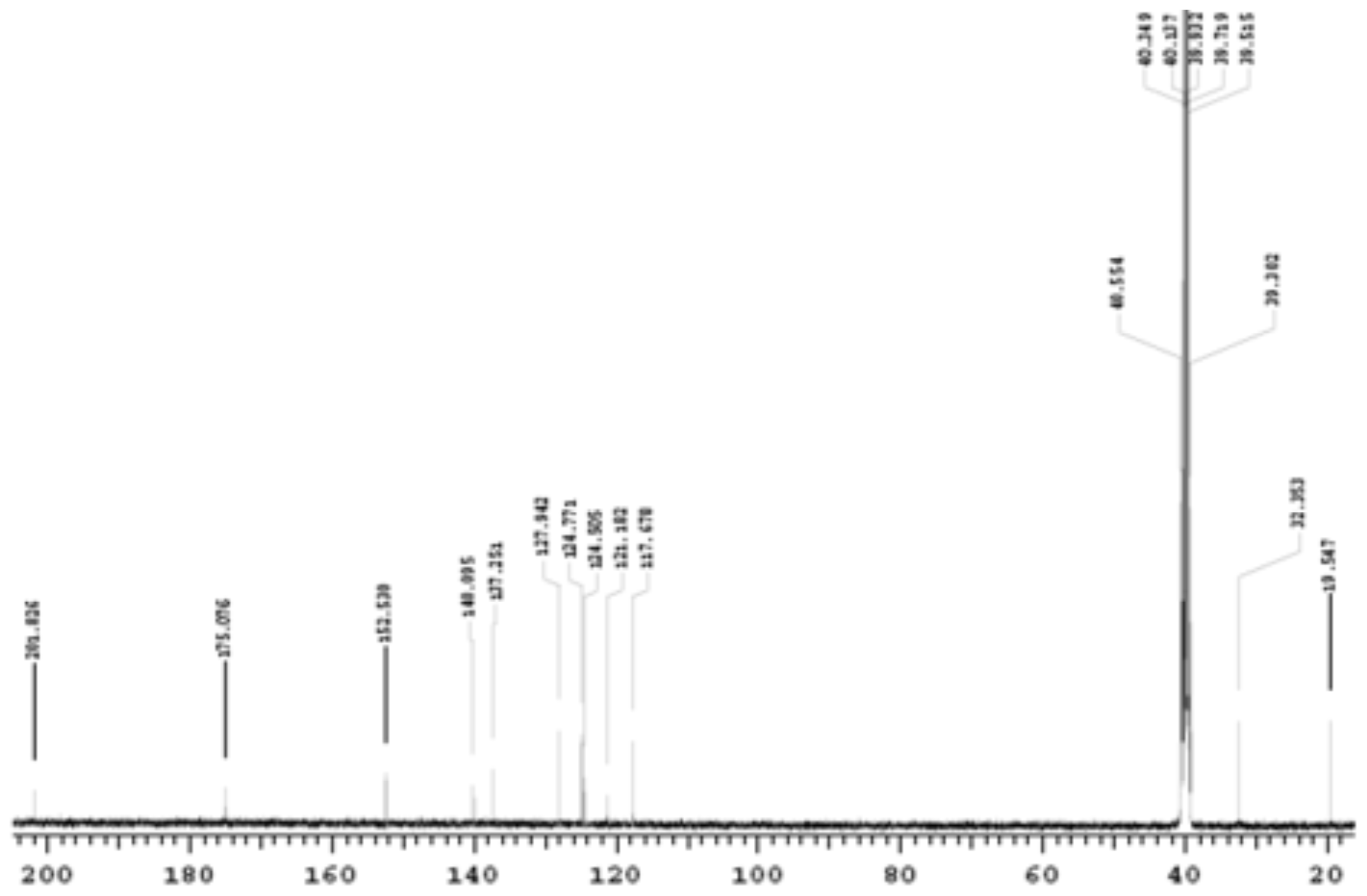

Supplement: Figure S14 — 13C Spectrum of 1-(7-Chloro-4-hydroxy-2-methylquinolin-3-yl)ethanone (3g). [file tjc-48-01-0097s14.tif]

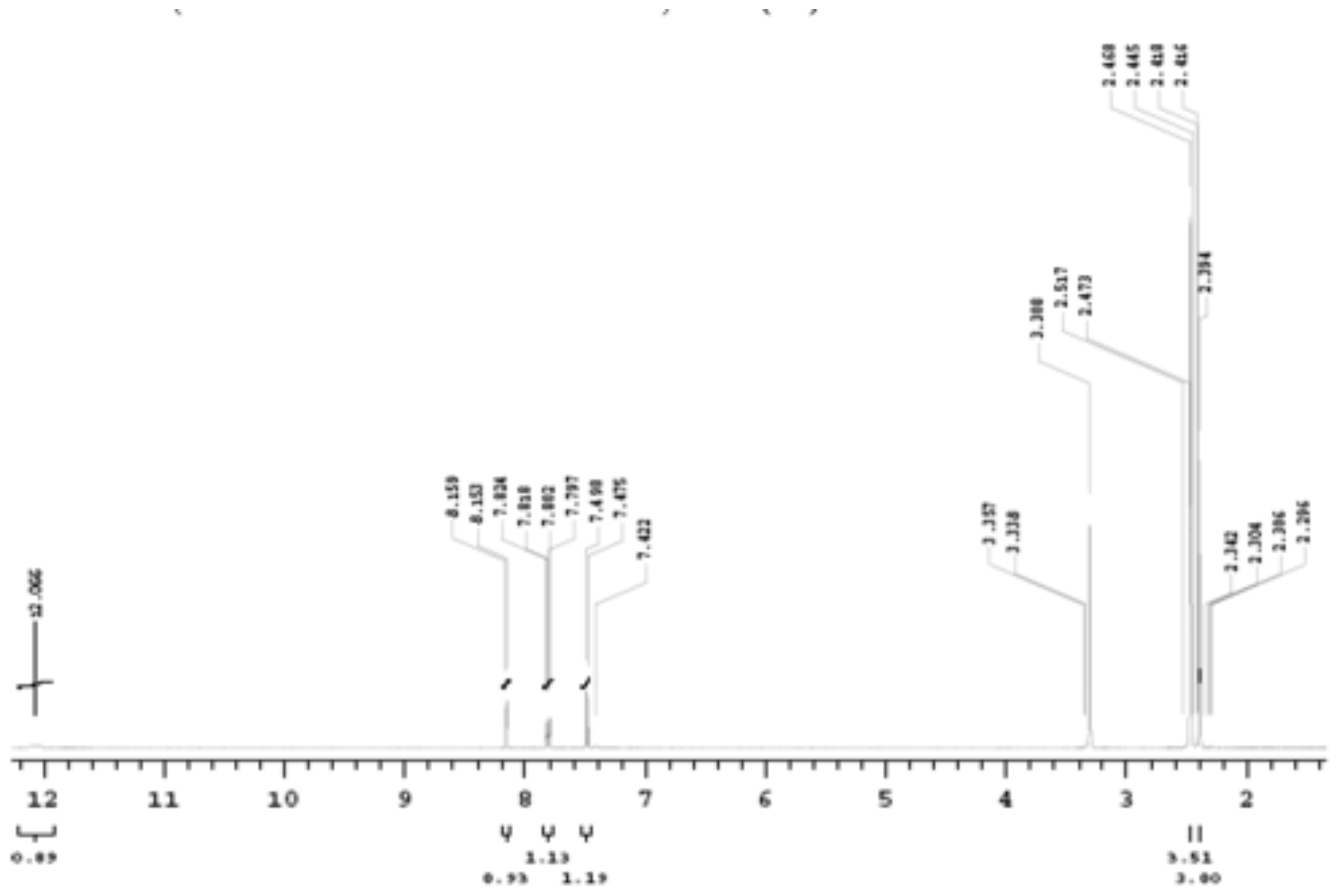

Supplement: Figure S15 — 1H Spectrum of 1-(6-Bromo-4-hydroxy-2-methylquinolin-3-yl)ethanone (3h). [file tjc-48-01-0097s15.tif]

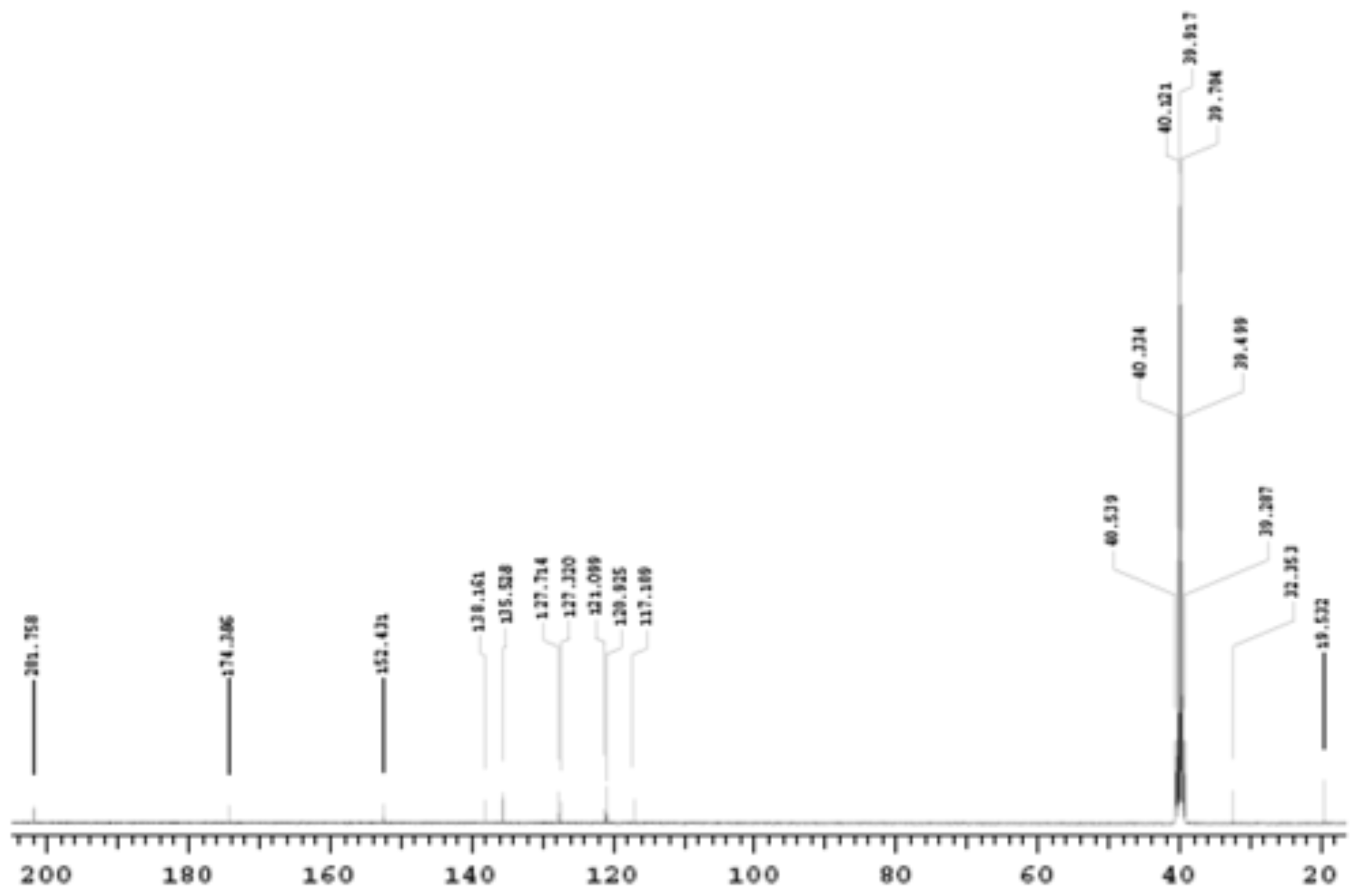

Supplement: Figure S16 — 13C Spectrum of 1-(6-Bromo-4-hydroxy-2-methylquinolin-3-yl)ethanone (3h). [file tjc-48-01-0097s16.tif]

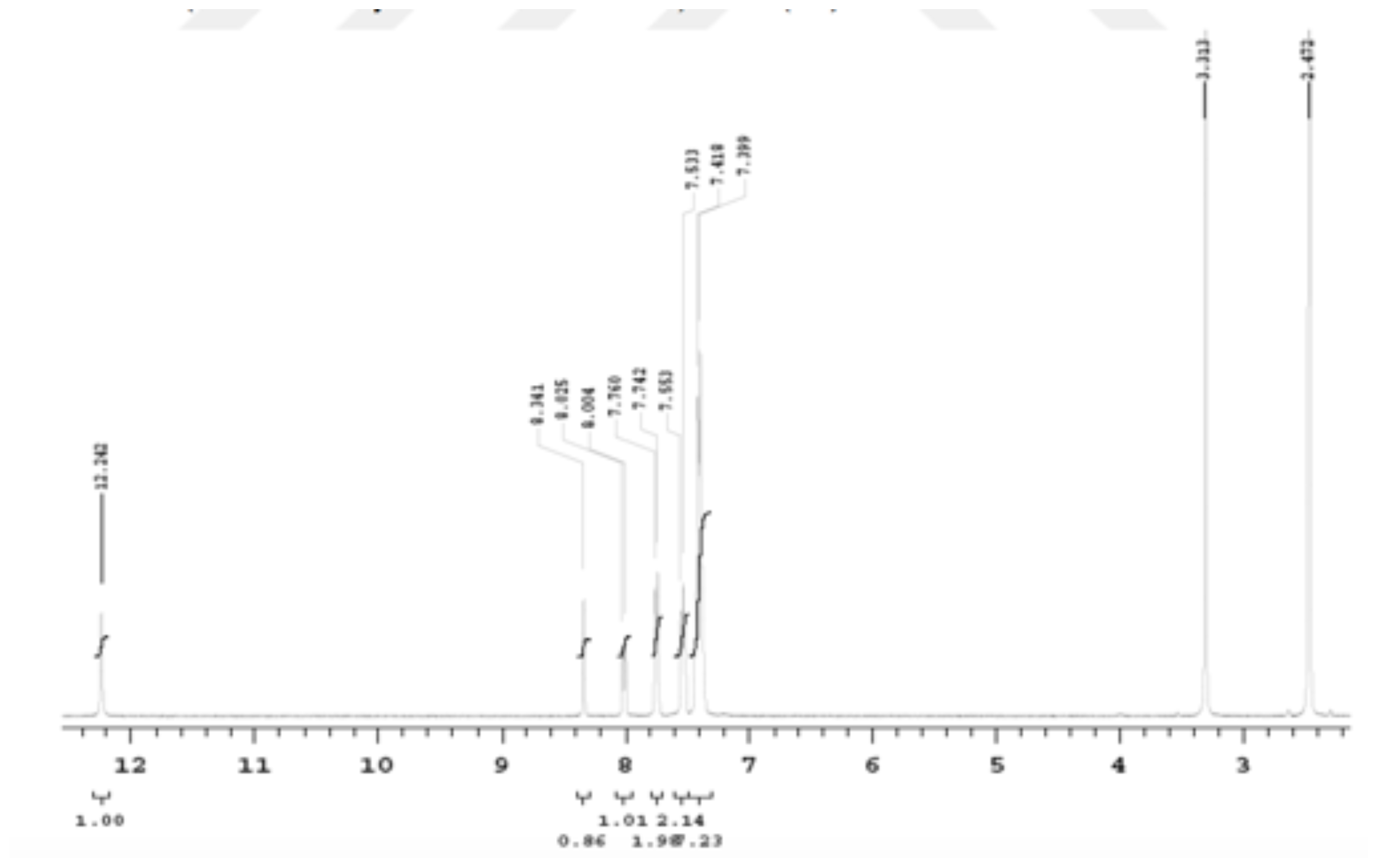

Supplement: Figure S17 — 1H Spectrum of (4-Hydroxy-6-iodo-2-phenylquinolin-3-yl)(phenyl)methanone (3i). [file tjc-48-01-0097s17.tif]

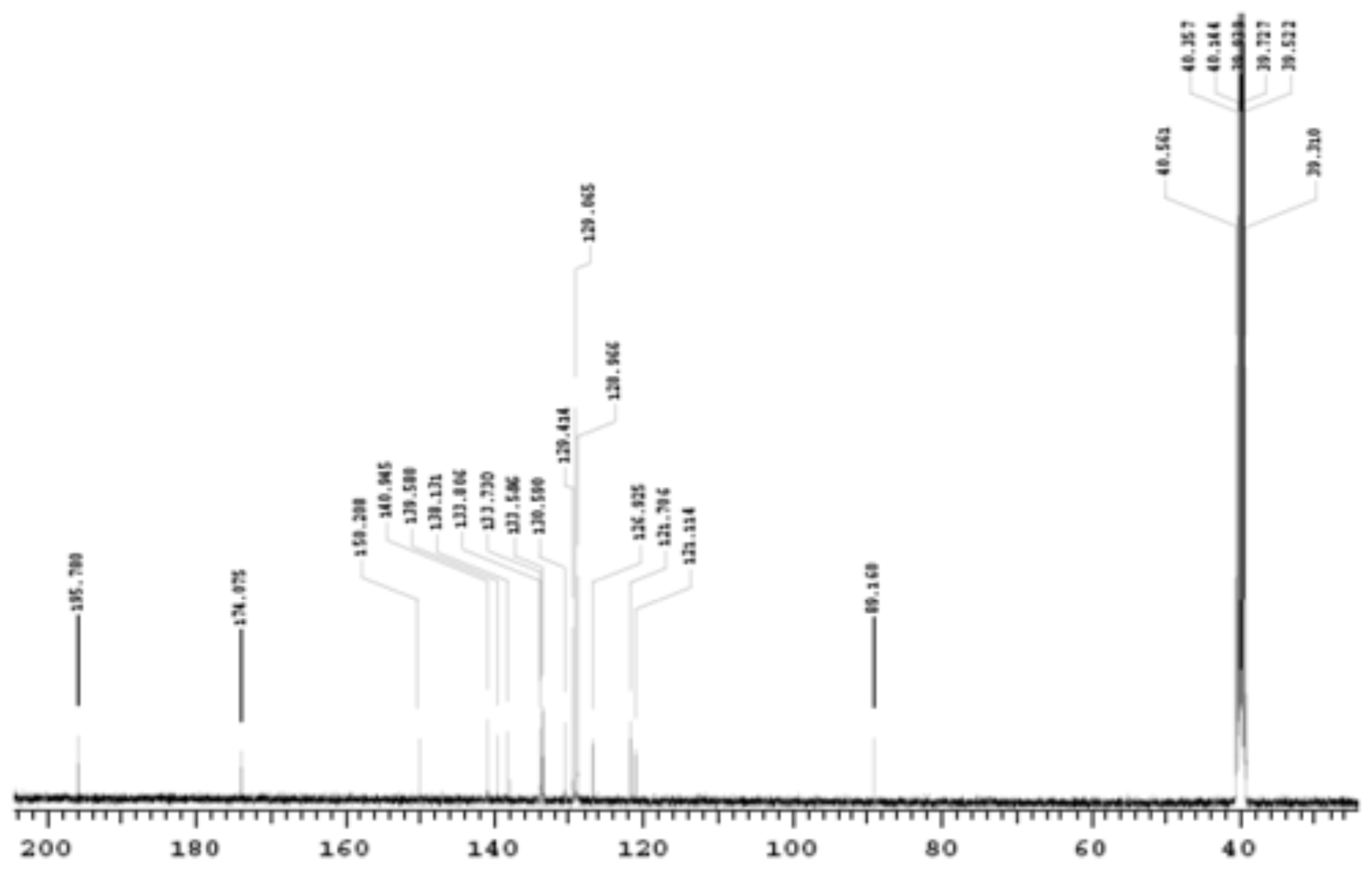

Supplement: Figure S18 — 13C Spectrum of (4-Hydroxy-6-iodo-2-phenylquinolin-3-yl)(phenyl)methanone (3i). [file tjc-48-01-0097s18.tif]

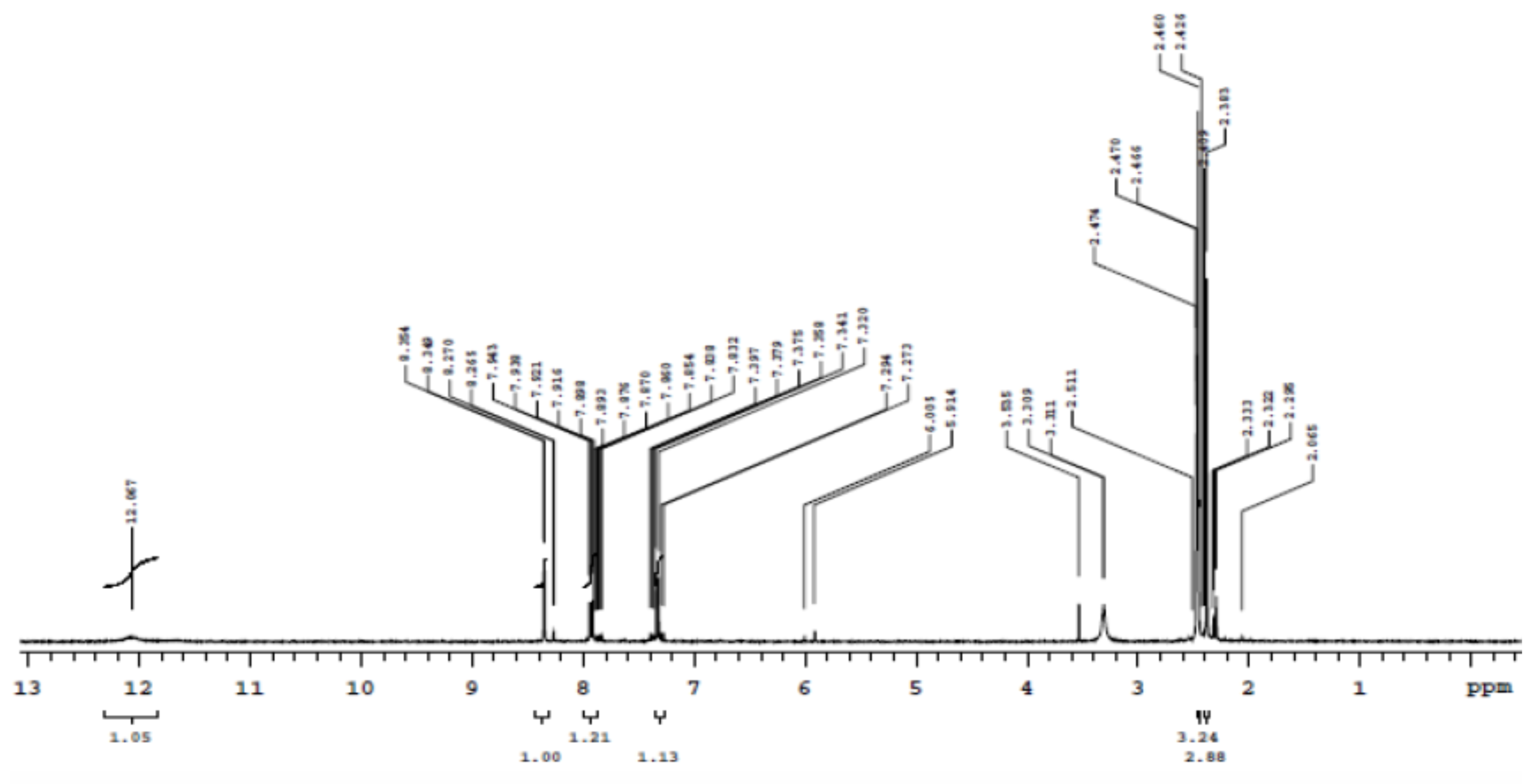

Supplement: Figure S19 — 1H Spectrum of 1-(4-Hydroxy-6-iodo-2-methylquinolin-3-yl)ethanone (3j). [file tjc-48-01-0097s19.tif]

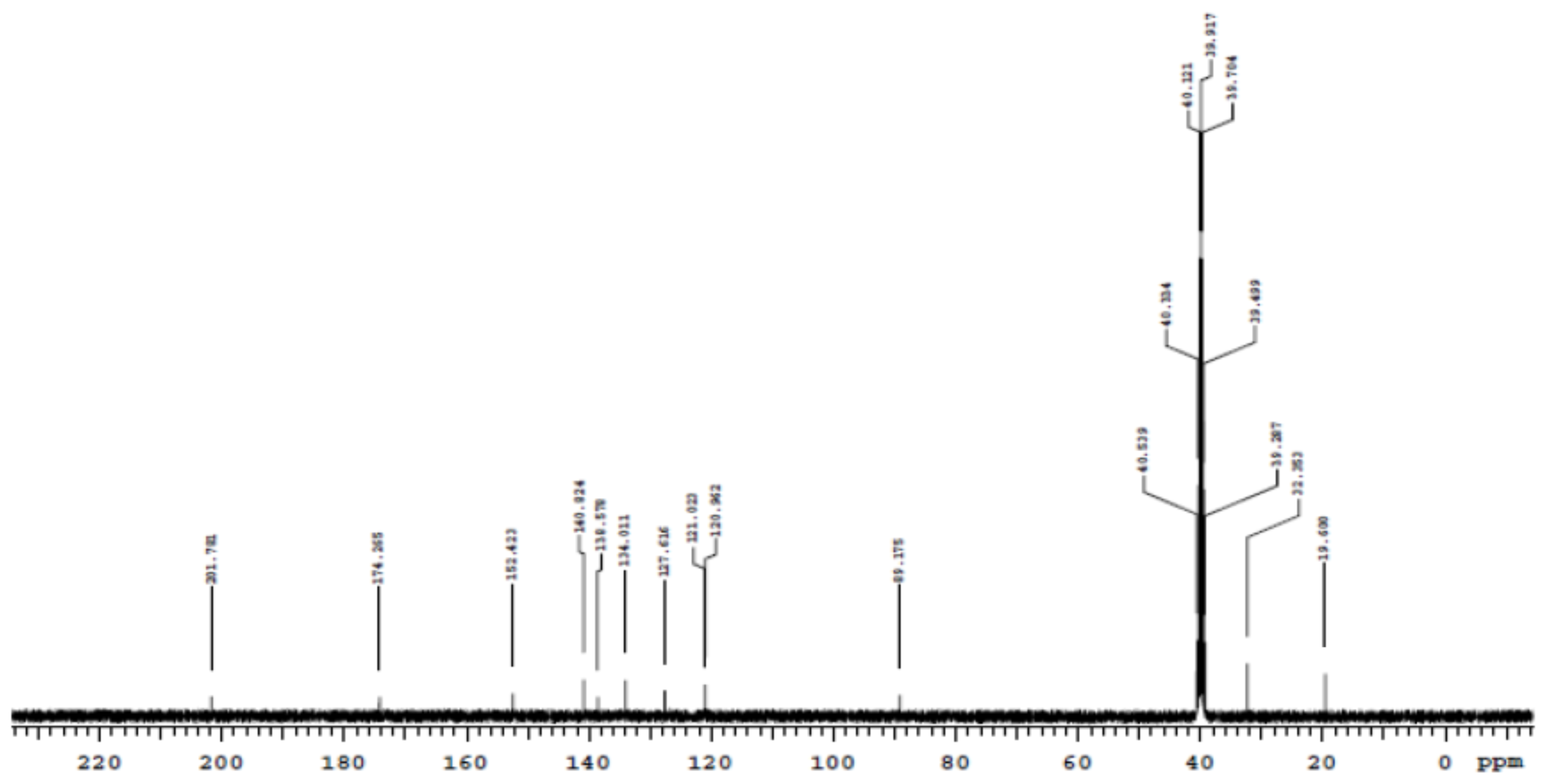

Supplement: Figure S20 — 13C Spectrum of 1-(4-Hydroxy-6-iodo-2-methylquinolin-3-yl)ethanone (3j). [file tjc-48-01-0097s20.tif]

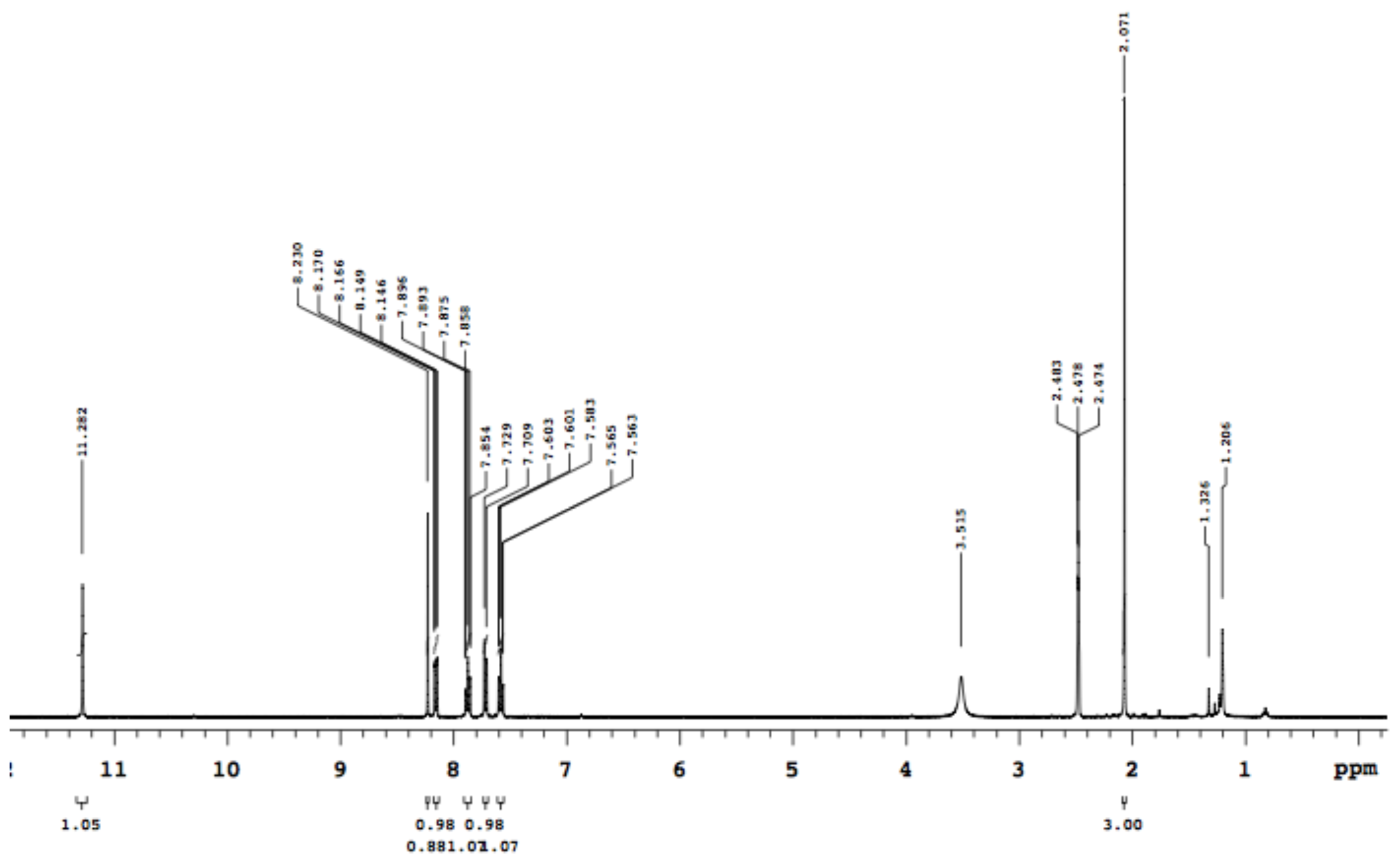

Supplement: Figure S21 — 1H Spectrum of N-(4-Oxoquinazolin-3(4H)-yl)acetamide (6a). [file tjc-48-01-0097s21.tif]

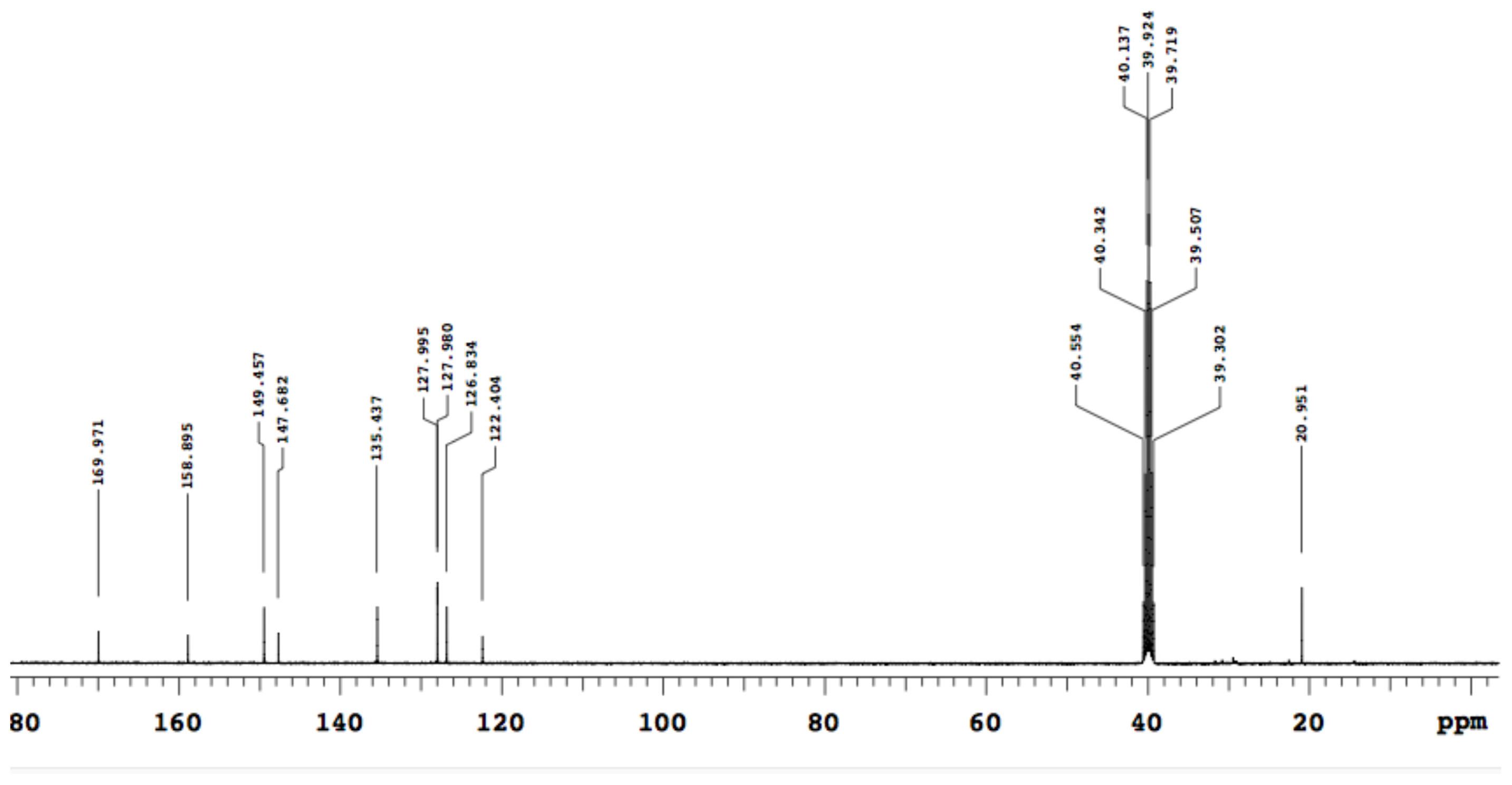

Supplement: Figure S22 — 13C Spectrum of N-(4-Oxoquinazolin-3(4H)-yl)acetamide (6a). [file tjc-48-01-0097s22.tif]

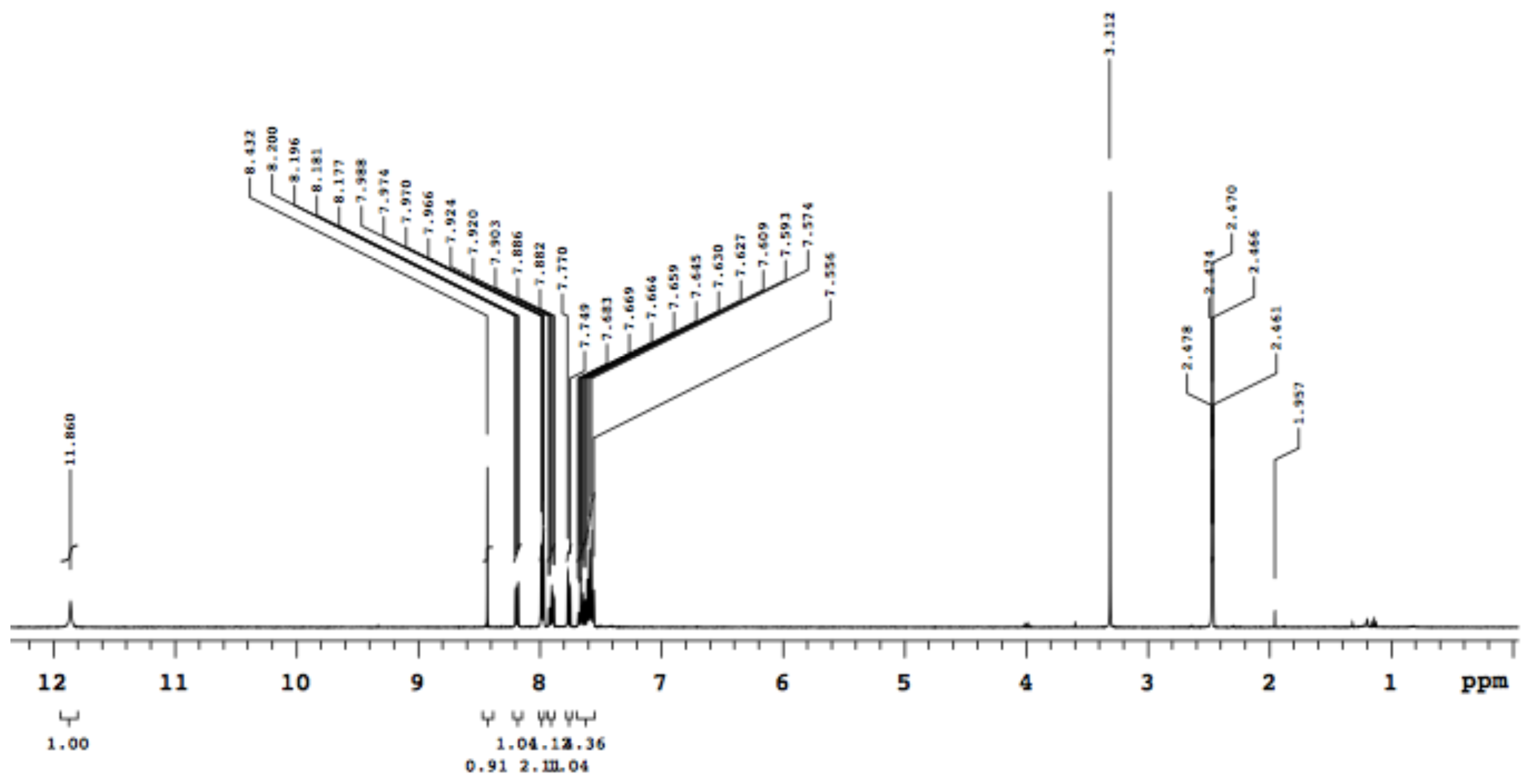

Supplement: Figure S23 — 1H Spectrum of N-(4-Oxoquinazolin-3(4H)-yl)benzamide (6b). [file tjc-48-01-0097s23.tif]

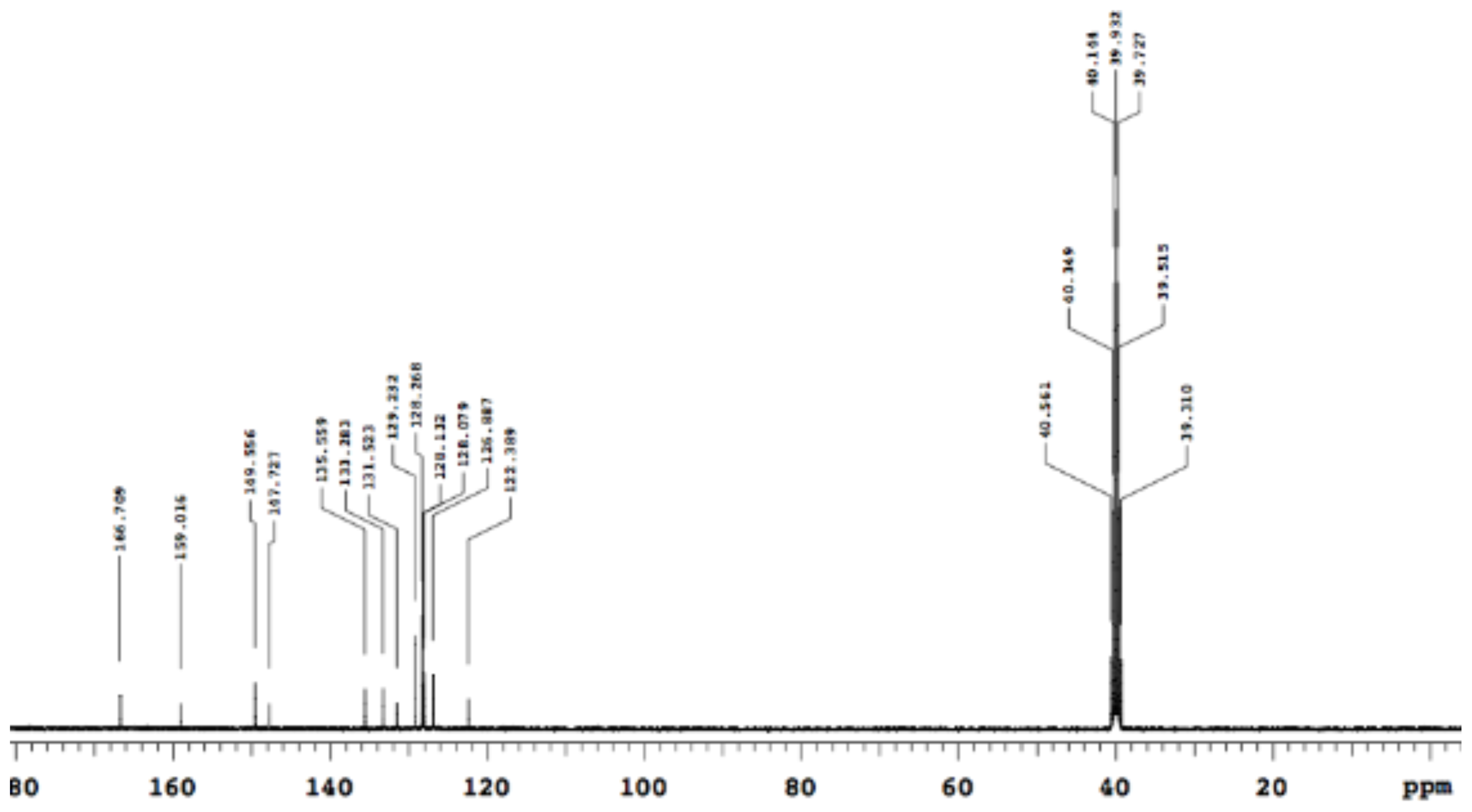

Supplement: Figure S24 — 13C Spectrum of N-(4-Oxoquinazolin-3(4H)-yl)benzamide (6b). [file tjc-48-01-0097s24.tif]

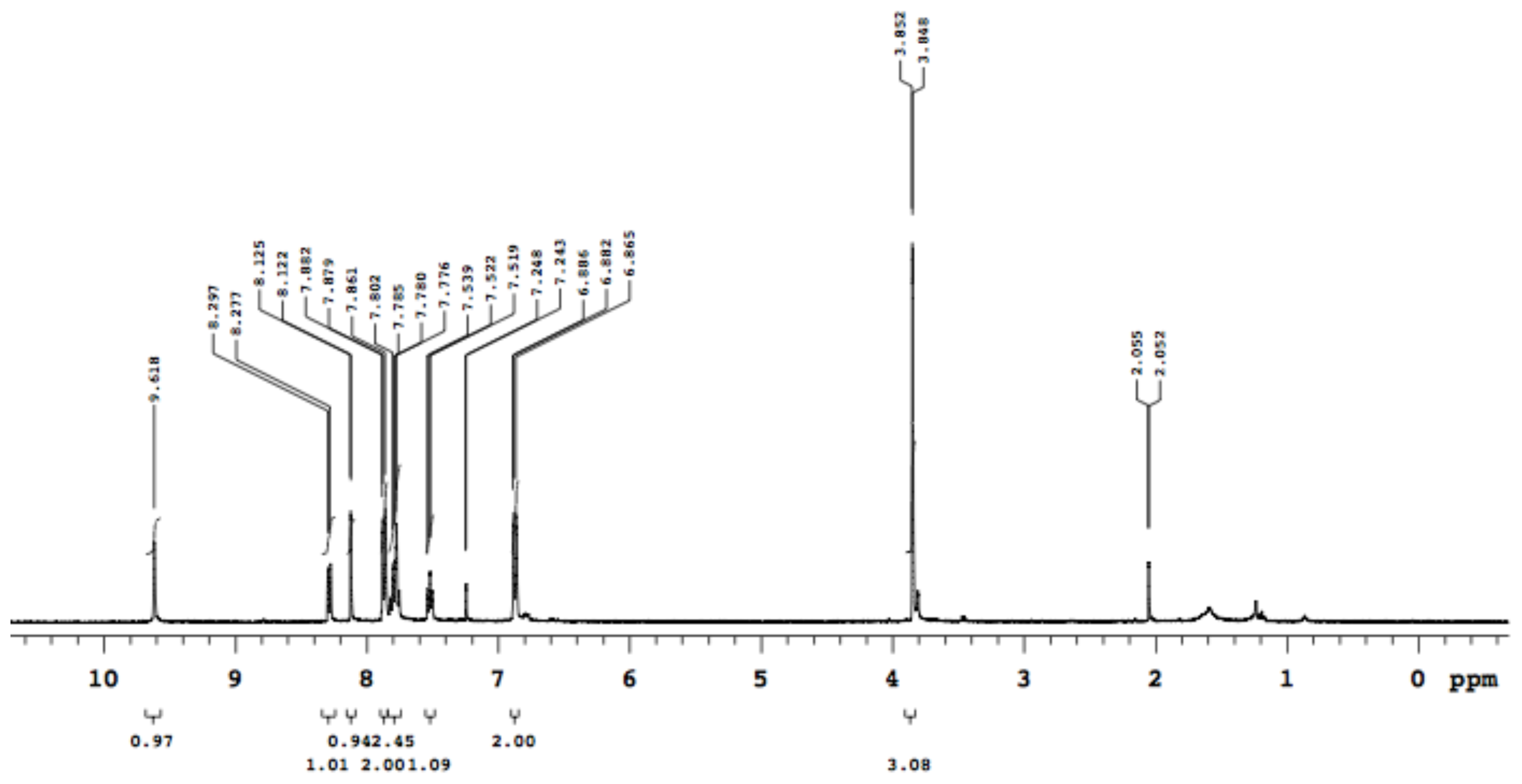

Supplement: Figure S25 — 1H Spectrum of 4-Methoxy-N-(4-oxoquinazolin-3(4H)-yl)benzamide (6c). [file tjc-48-01-0097s25.tif]

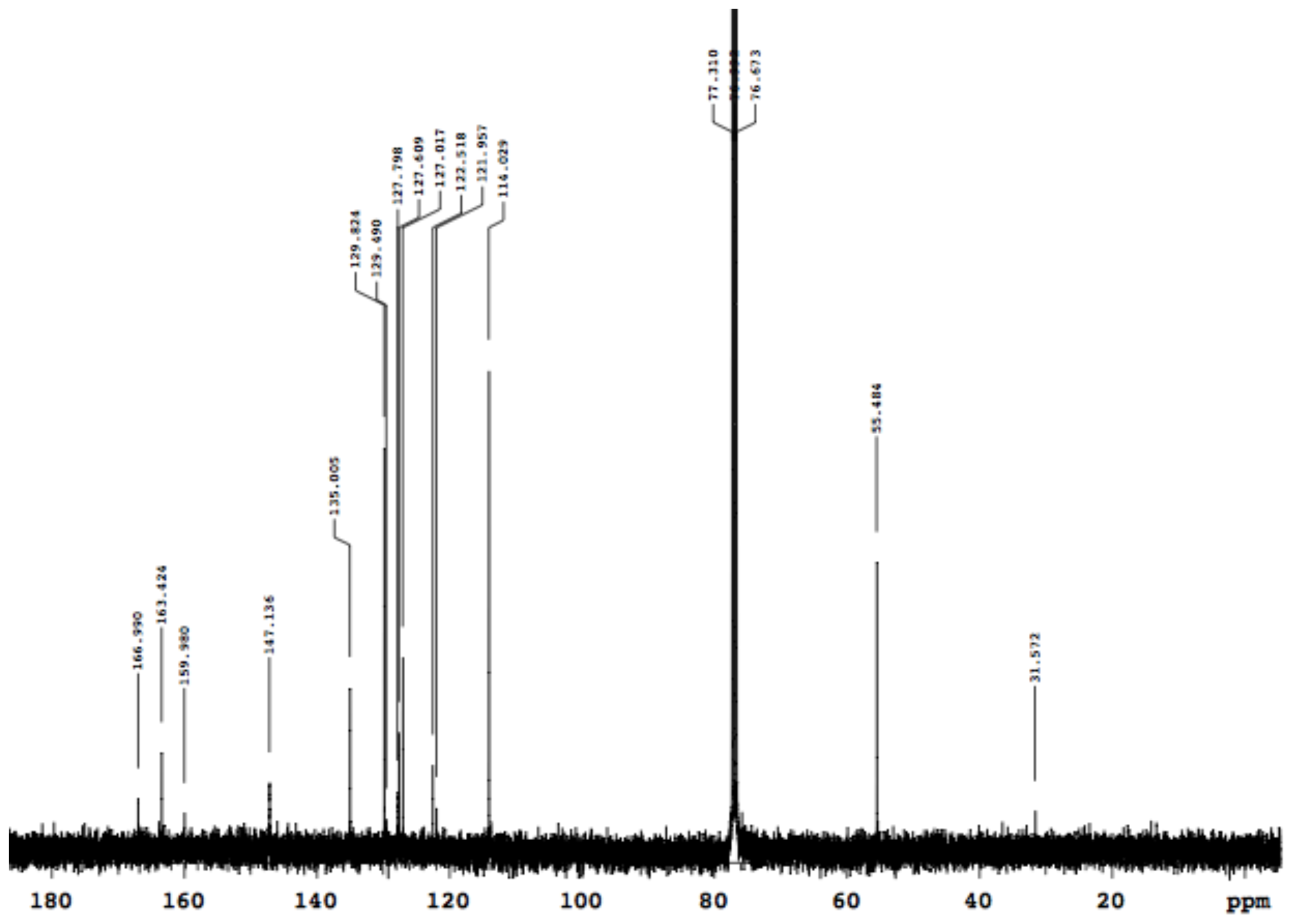

Supplement: Figure S26 — 13C Spectrum of 4-Methoxy-N-(4-oxoquinazolin-3(4H)-yl)benzamide (6c). [file tjc-48-01-0097s26.tif]

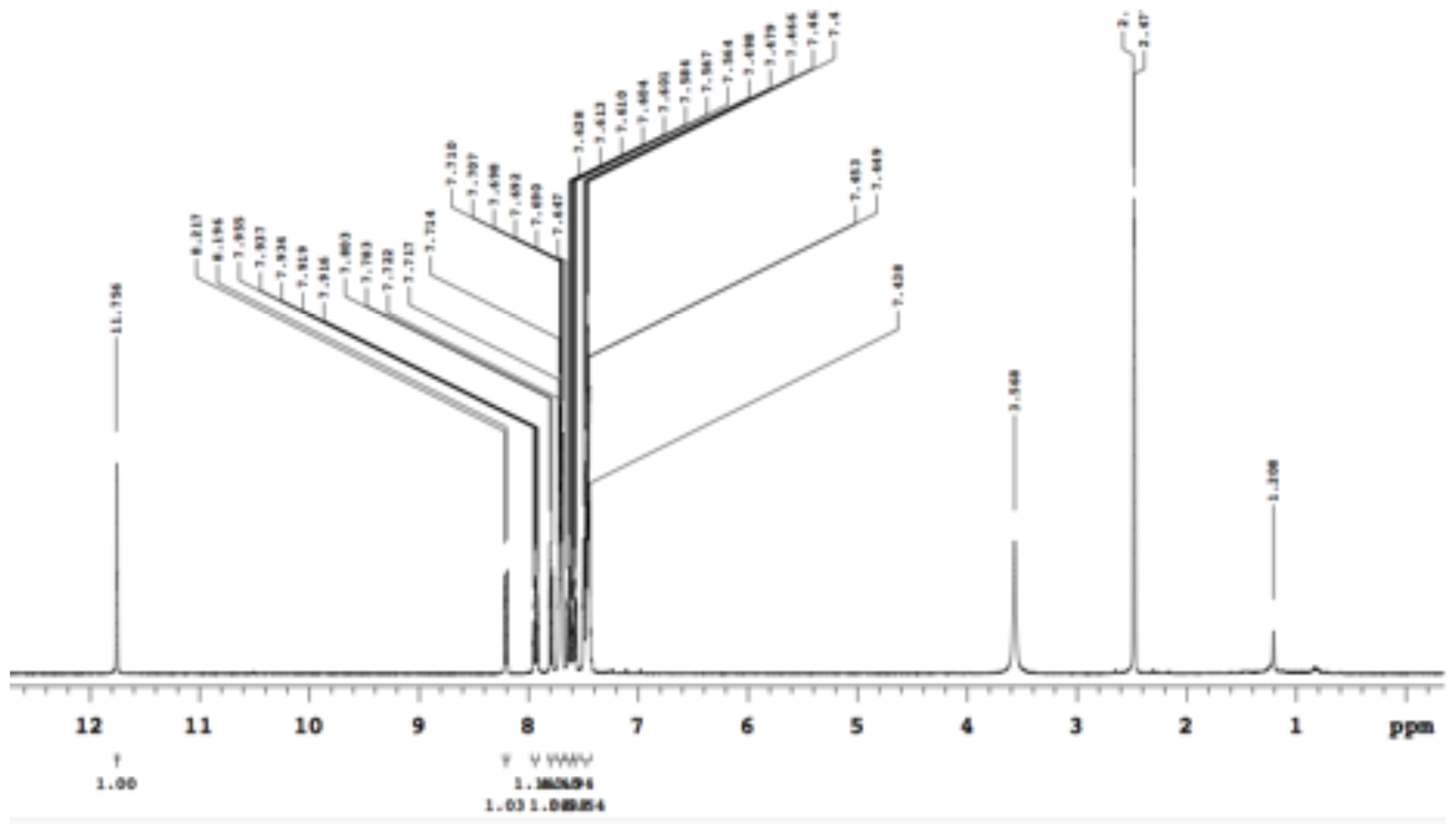

Supplement: Figure S27 — 1H Spectrum of N-(4-Oxo-2-phenylquinazolin-3(4H)-yl)benzamide (6d). [file tjc-48-01-0097s27.tif]

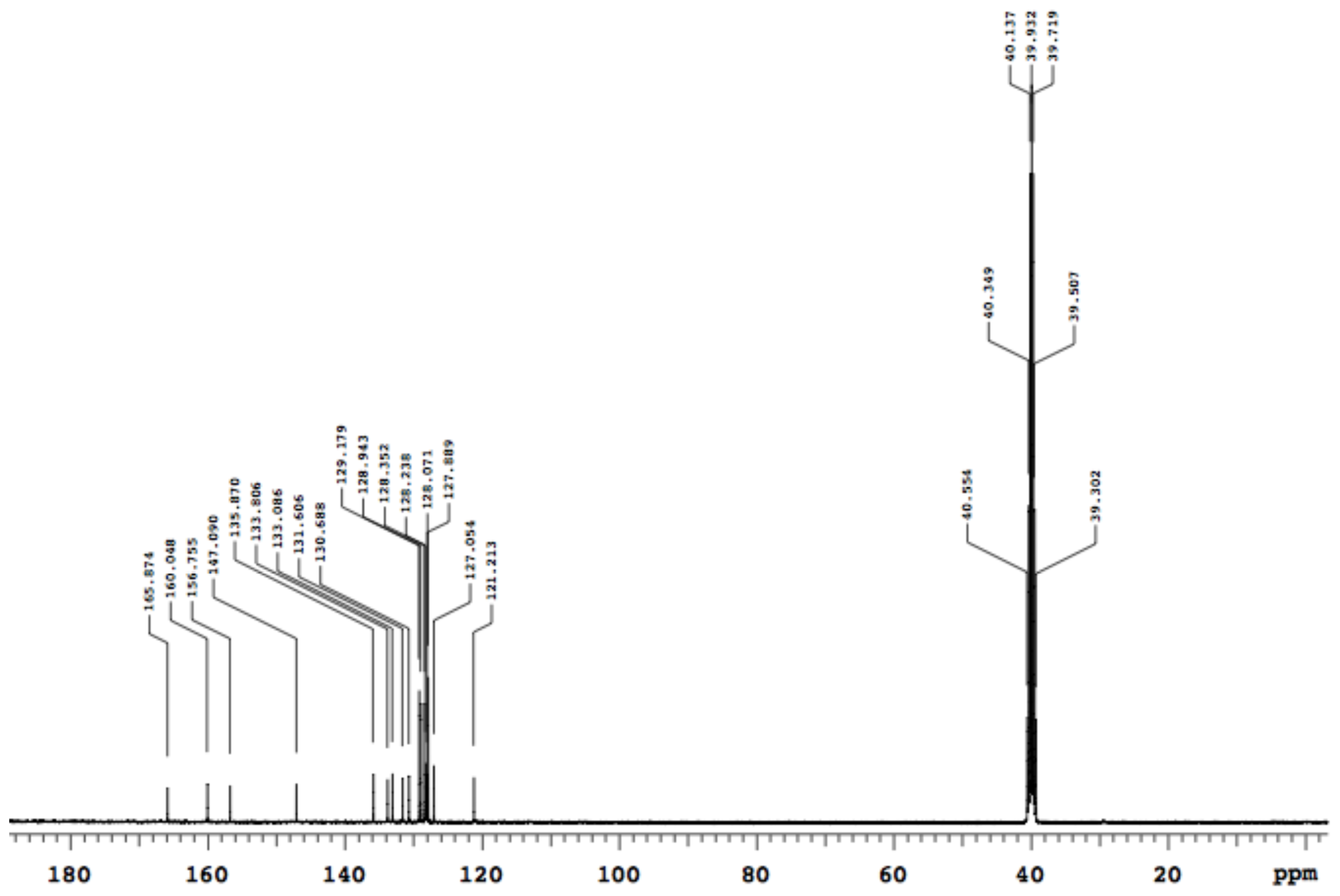

Supplement: Figure S28 — 13C Spectrum of N-(4-Oxo-2-phenylquinazolin-3(4H)-yl)benzamide (6d). [file tjc-48-01-0097s28.tif]

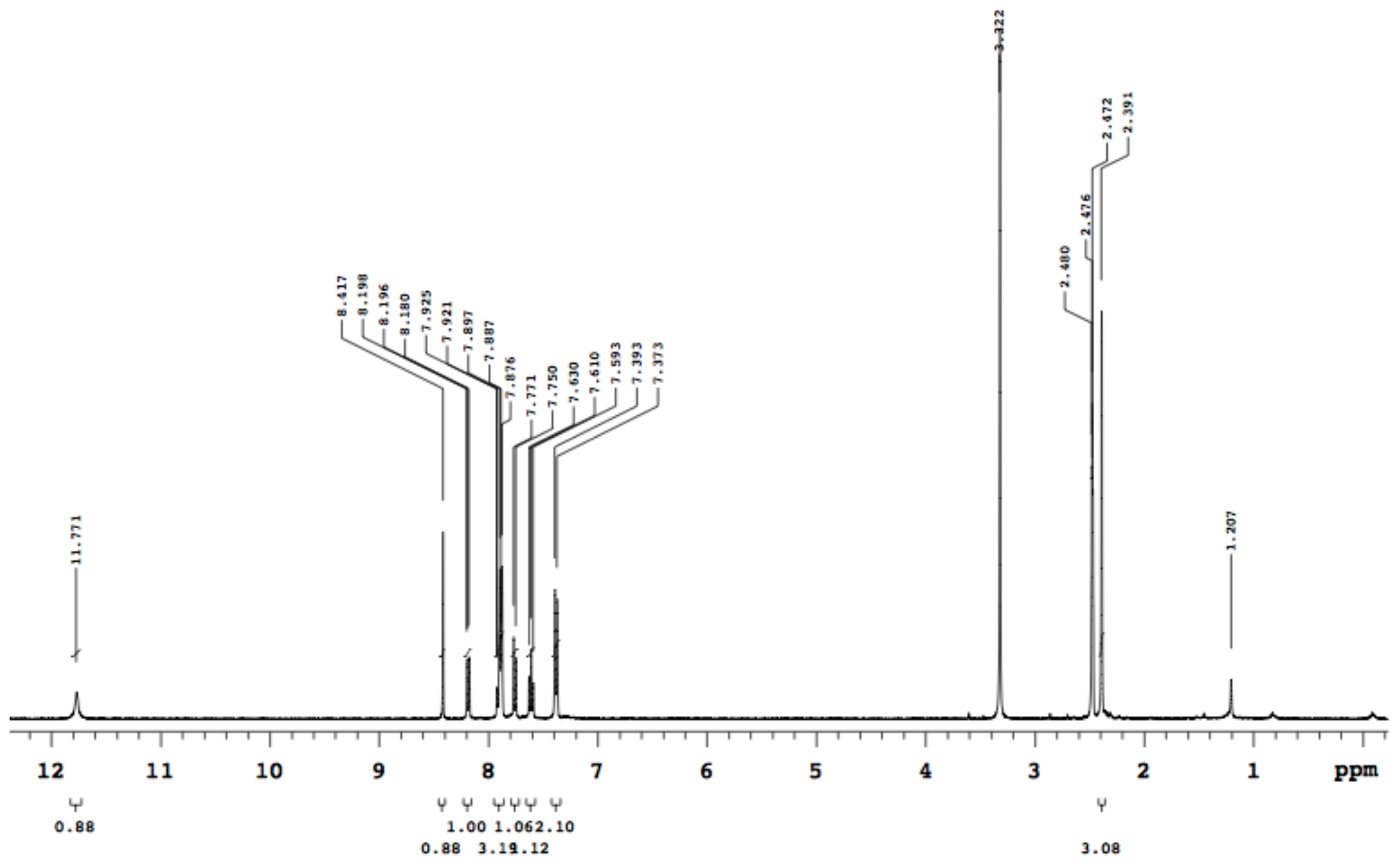

Supplement: Figure S29 — 1H Spectrum of 4-Methyl-N-(4-oxoquinazolin-3(4H)-yl)benzamide (6e). [file tjc-48-01-0097s29.tif]

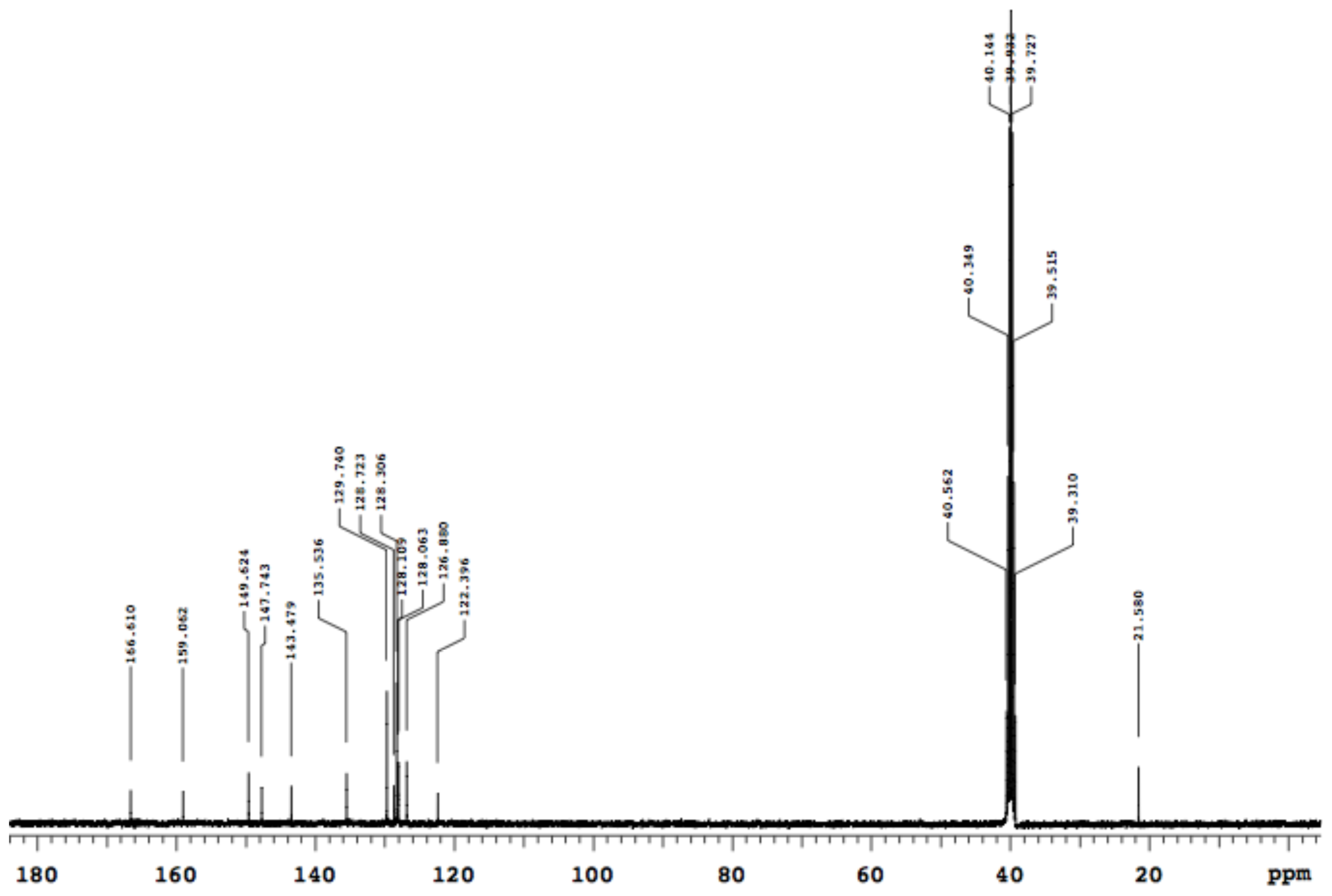

Supplement: Figure S30 — 13C Spectrum of 4-Methyl-N-(4-oxoquinazolin-3(4H)-yl)benzamide (6e). [file tjc-48-01-0097s30.tif]

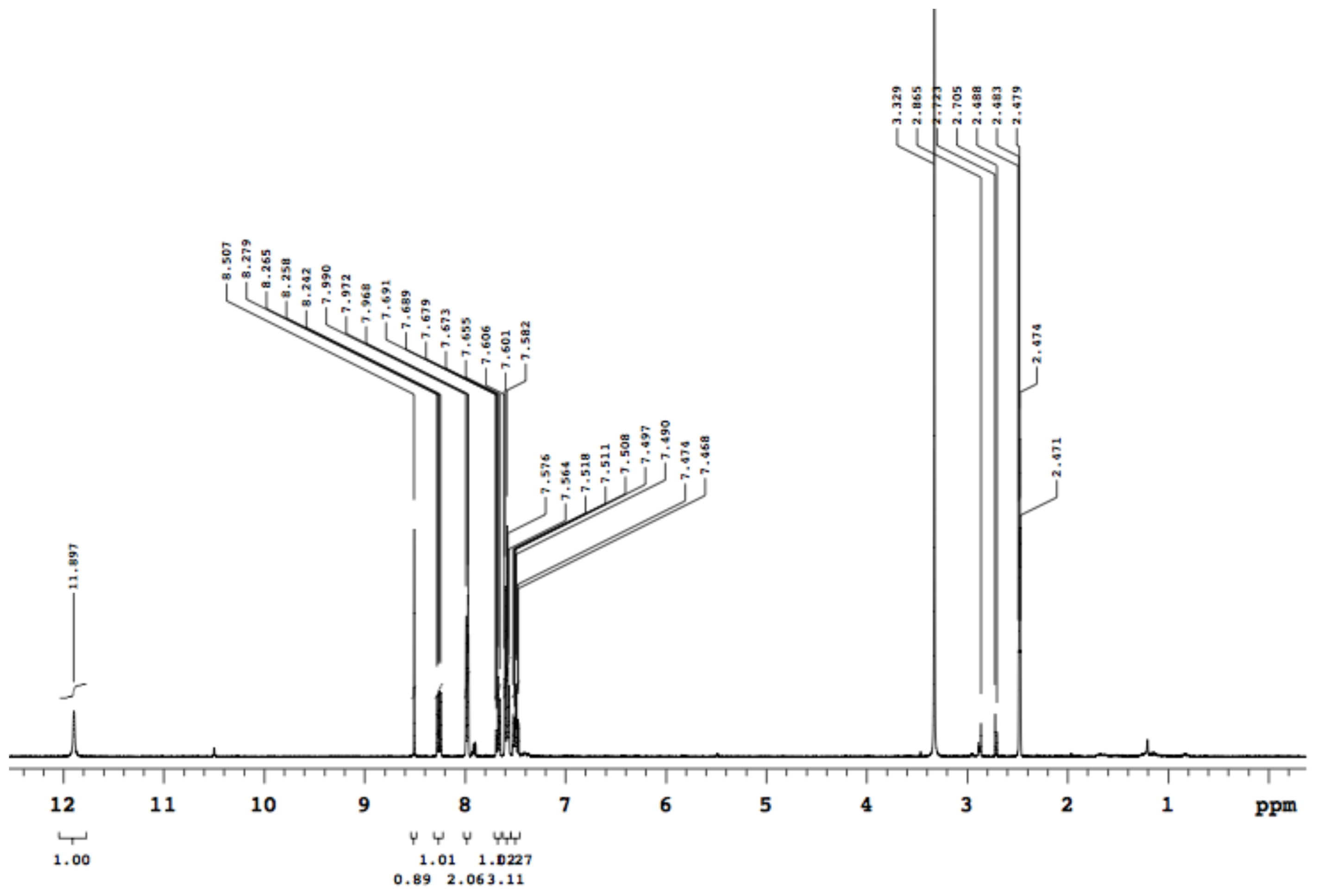

Supplement: Figure S31 — 1H Spectrum of N-(7-Fluoro-4-oxoquinazolin-3(4H)-yl)benzamide (6f). [file tjc-48-01-0097s31.tif]

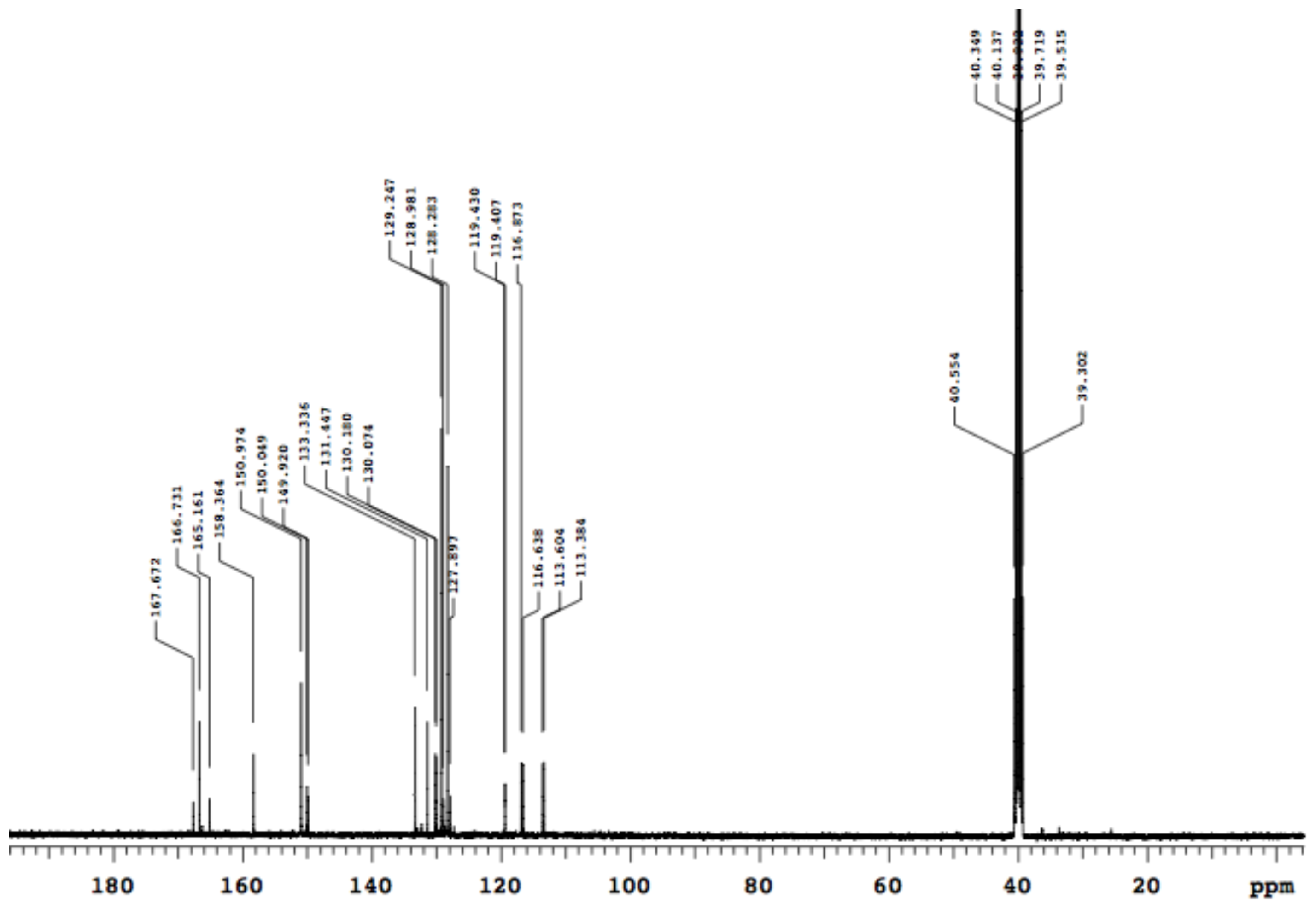

Supplement: Figure S32 — 1H Spectrum of N-(7-Fluoro-4-oxoquinazolin-3(4H)-yl)benzamide (6f). [file tjc-48-01-0097s32.tif]

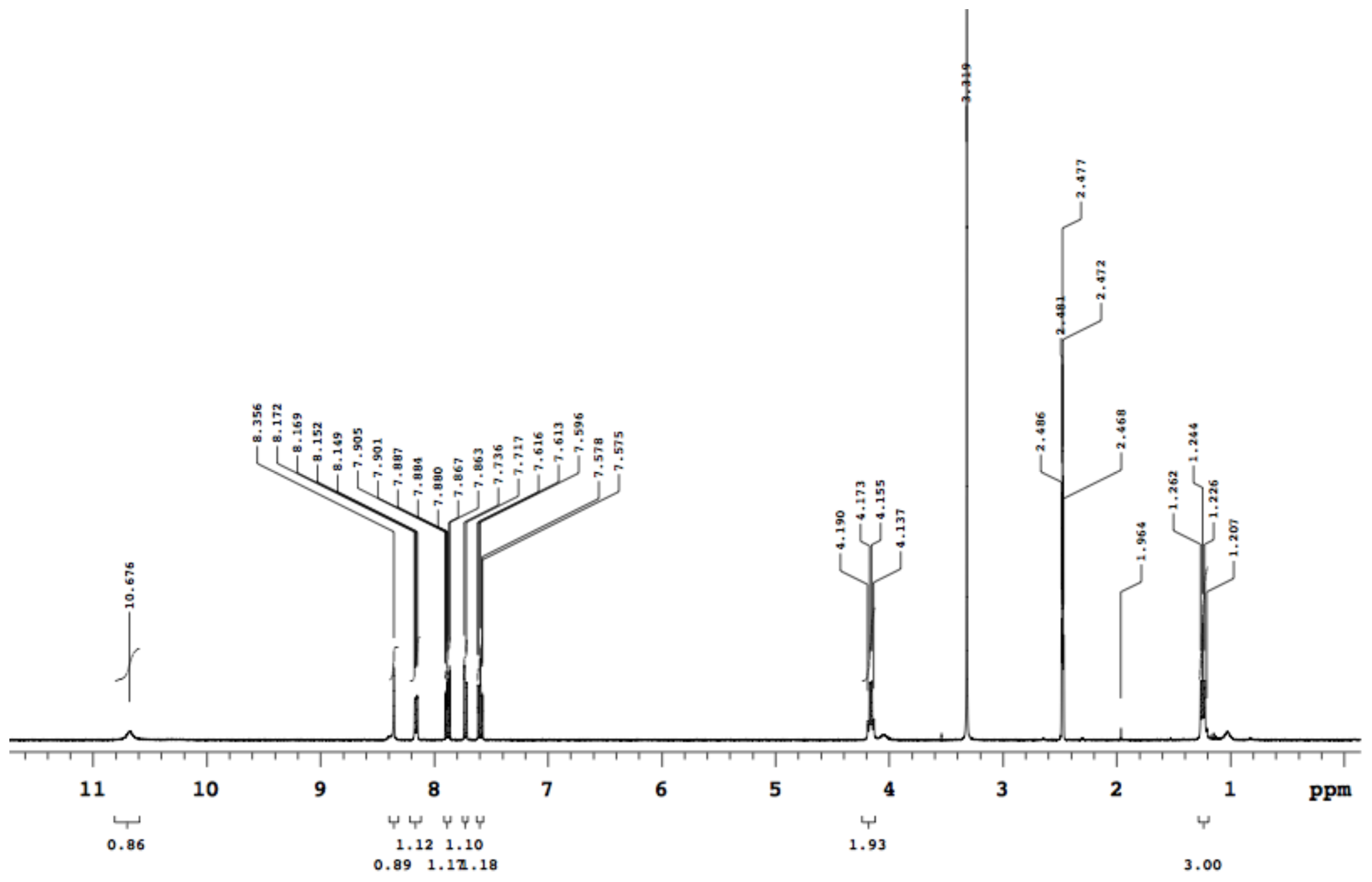

Supplement: Figure S33 — 1H Spectrum of Ethyl (4-oxoquinazolin-3(4H)-yl)carbamate (6g). [file tjc-48-01-0097s33.tif]

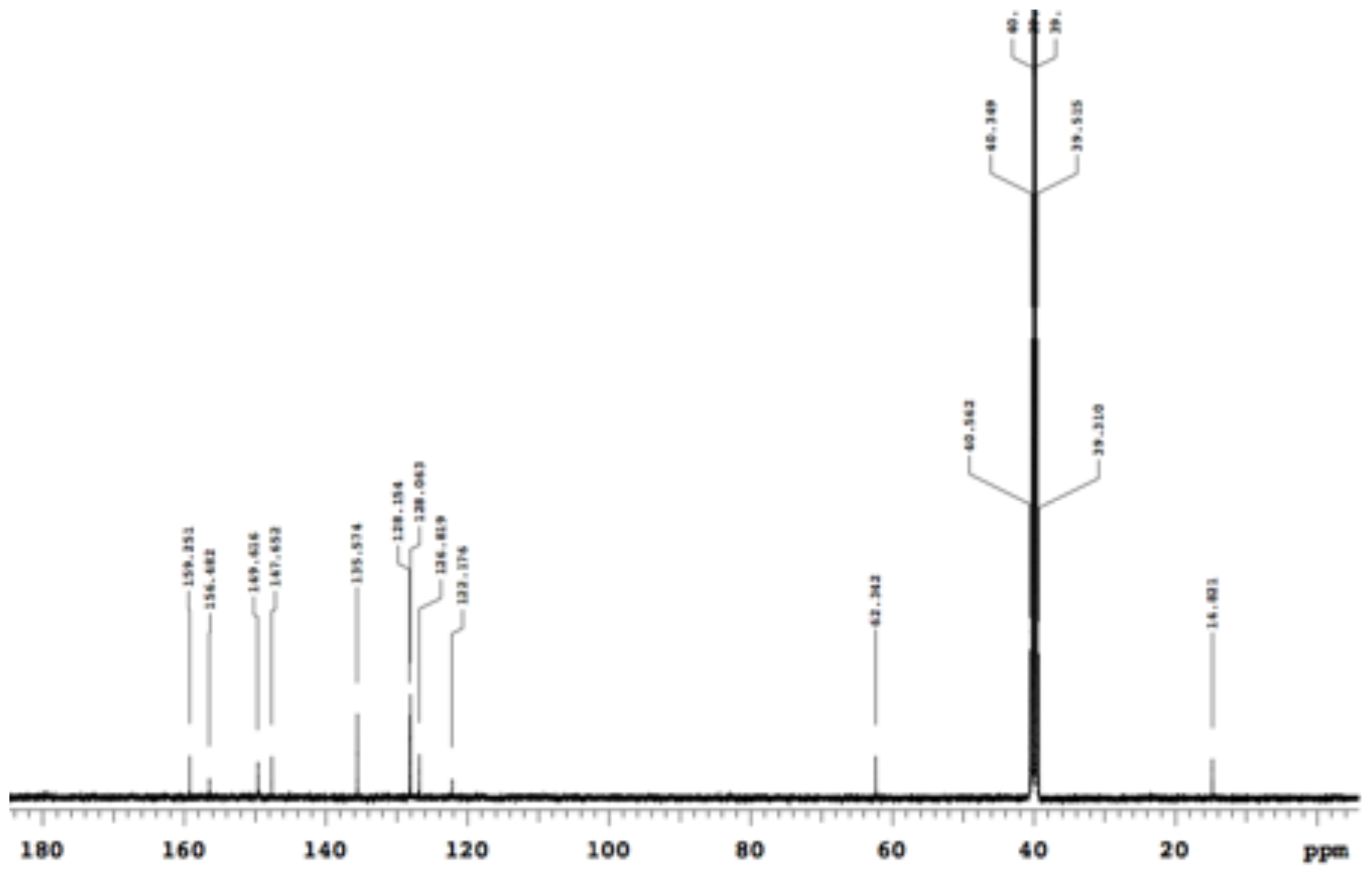

Supplement: Figure S34 — 13C Spectrum of Ethyl (4-oxoquinazolin-3(4H)-yl)carbamate (6g). [file tjc-48-01-0097s34.tif]

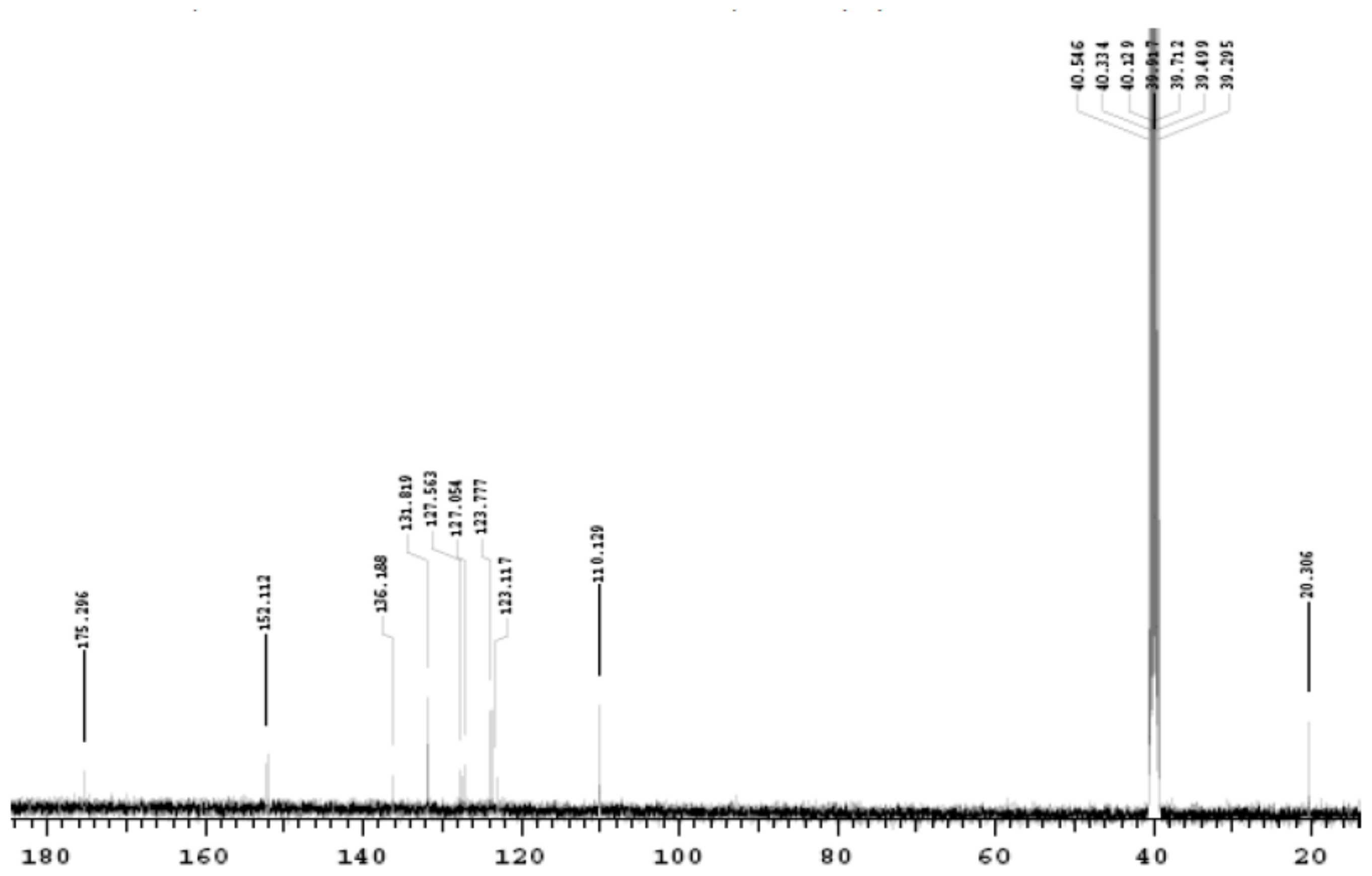

Supplement: Figure S35 — 1H Spectrum of Ethyl (2-methyl-4-oxoquinazolin-3(4H)-yl)carbamate (6h). [file tjc-48-01-0097s35.tif]

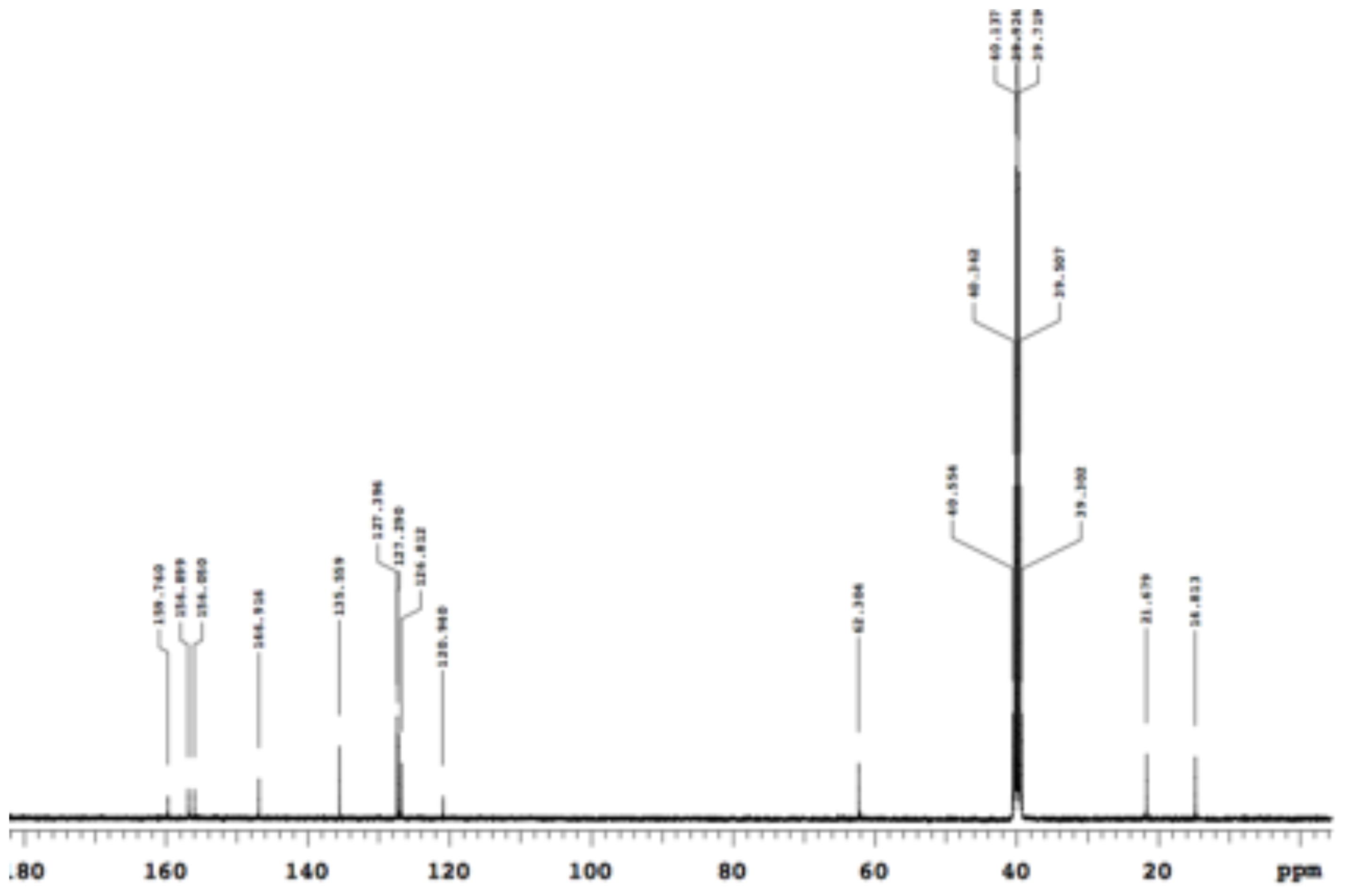

Supplement: Figure S36 — 13C Spectrum of Ethyl (2-methyl-4-oxoquinazolin-3(4H)-yl)carbamate (6h). [file tjc-48-01-0097s36.tif]

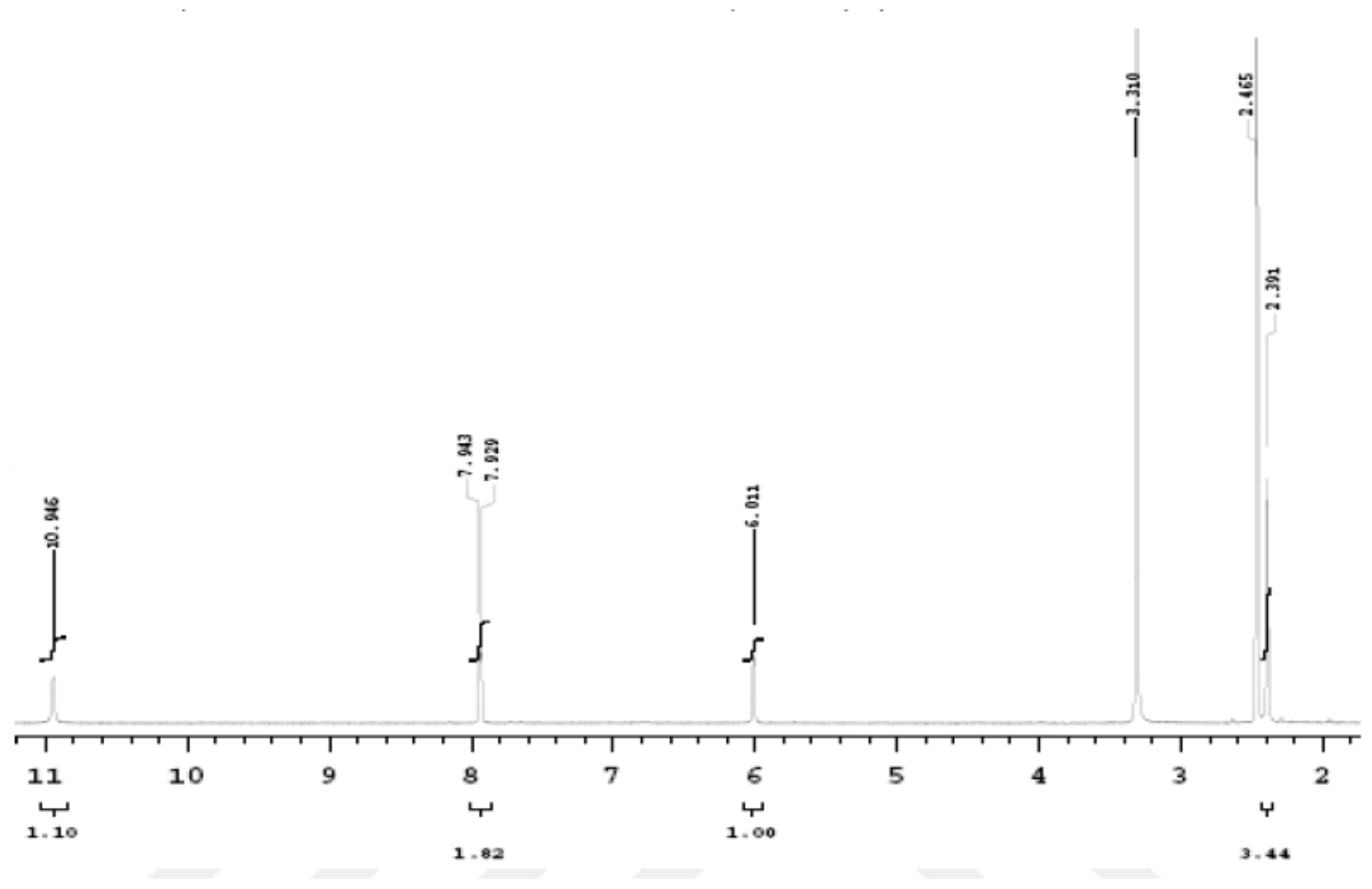

Supplement: Figure S37 — 1H Spectrum of 6,8-dichloro-2-methylquinolin-4-ol (3kb). [file tjc-48-01-0097s37.tif]

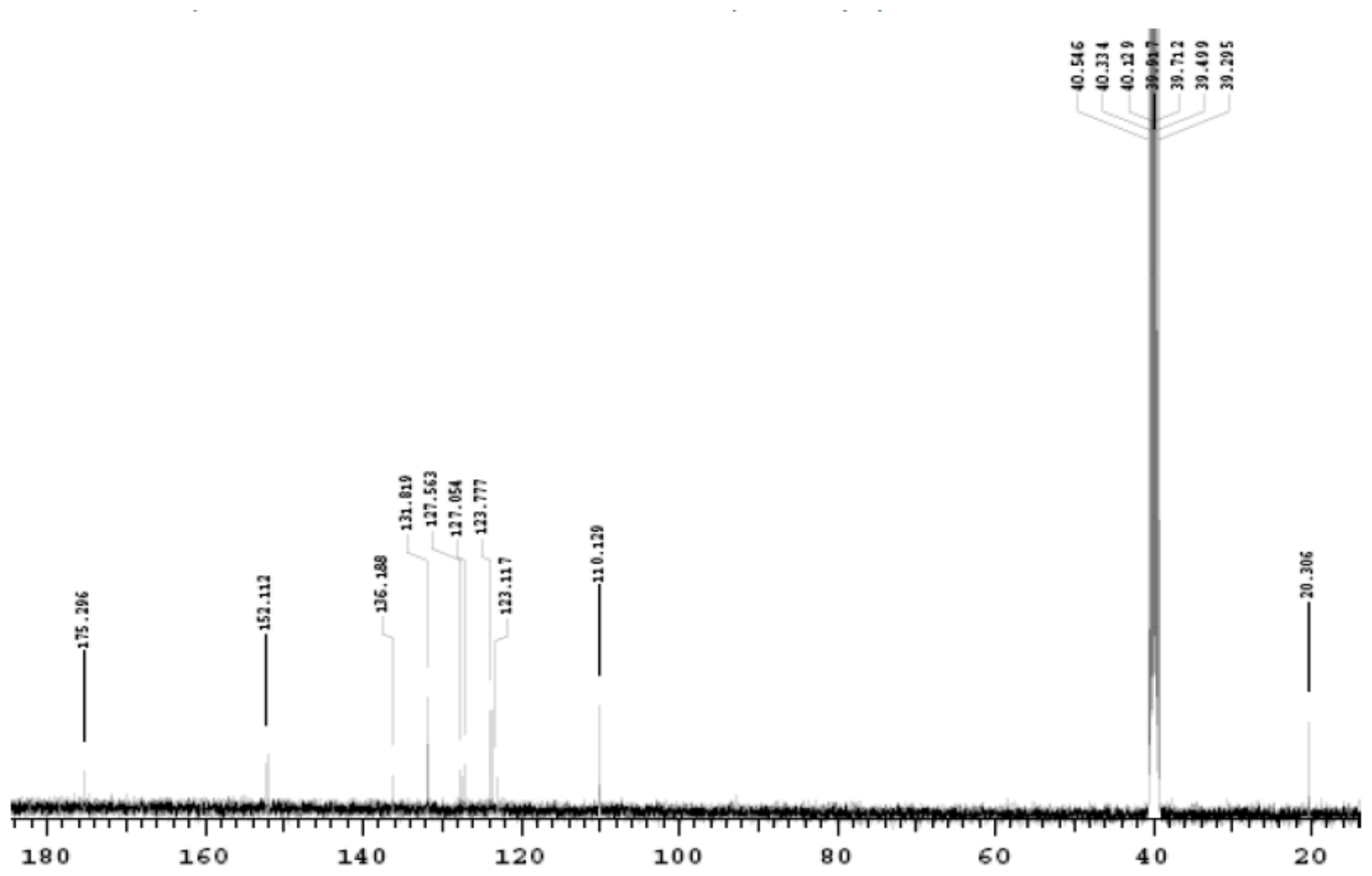

Supplement: Figure S38 — 13C Spectrum of 6,8-dichloro-2-methylquinolin-4-ol (3kb). [file tjc-48-01-0097s38.tif]

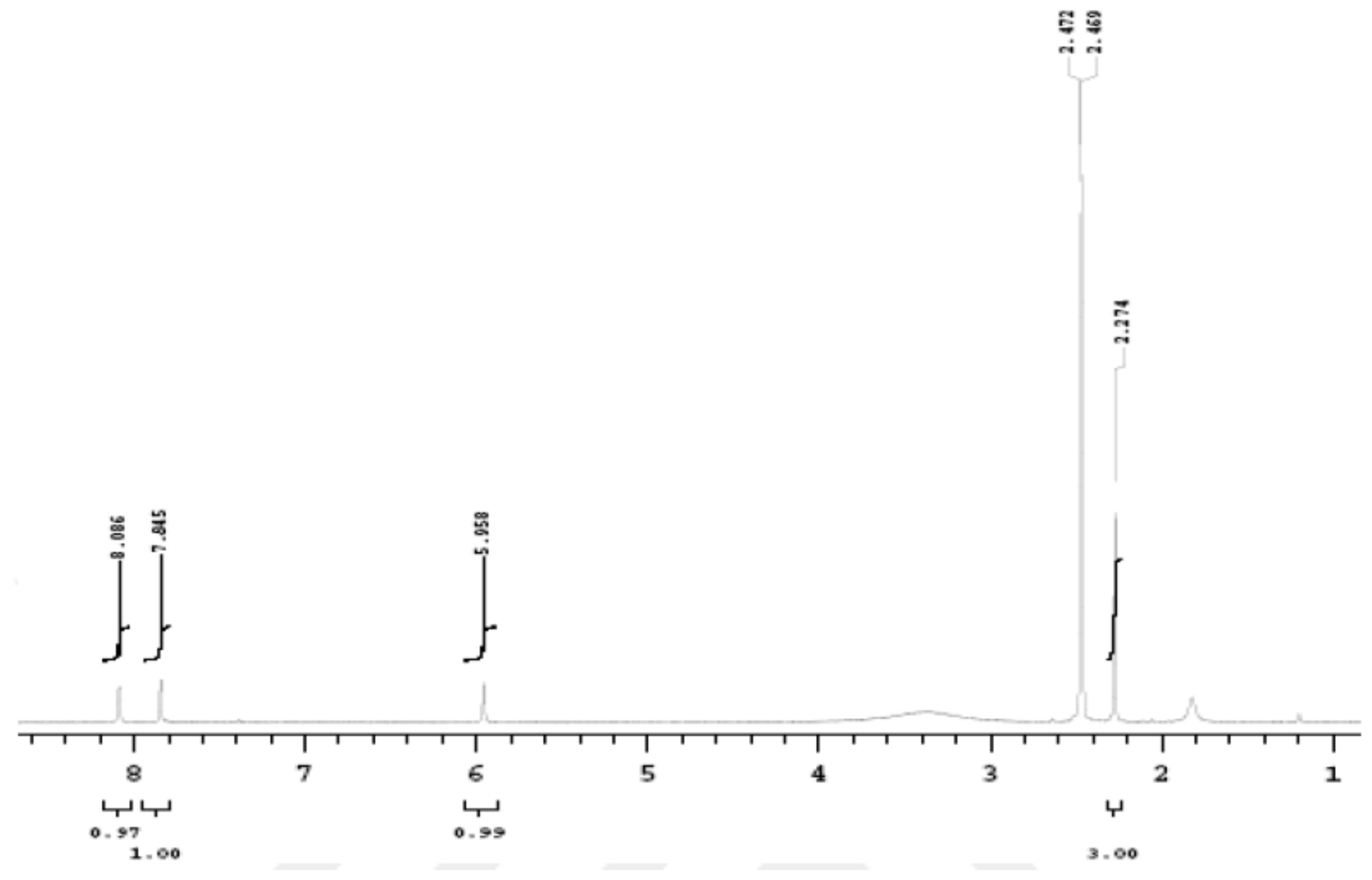

Supplement: Figure S39 — 1H Spectrum of 6,8-dibromo-2-methylquinolin-4-ol (3lb). [file tjc-48-01-0097s39.tif]

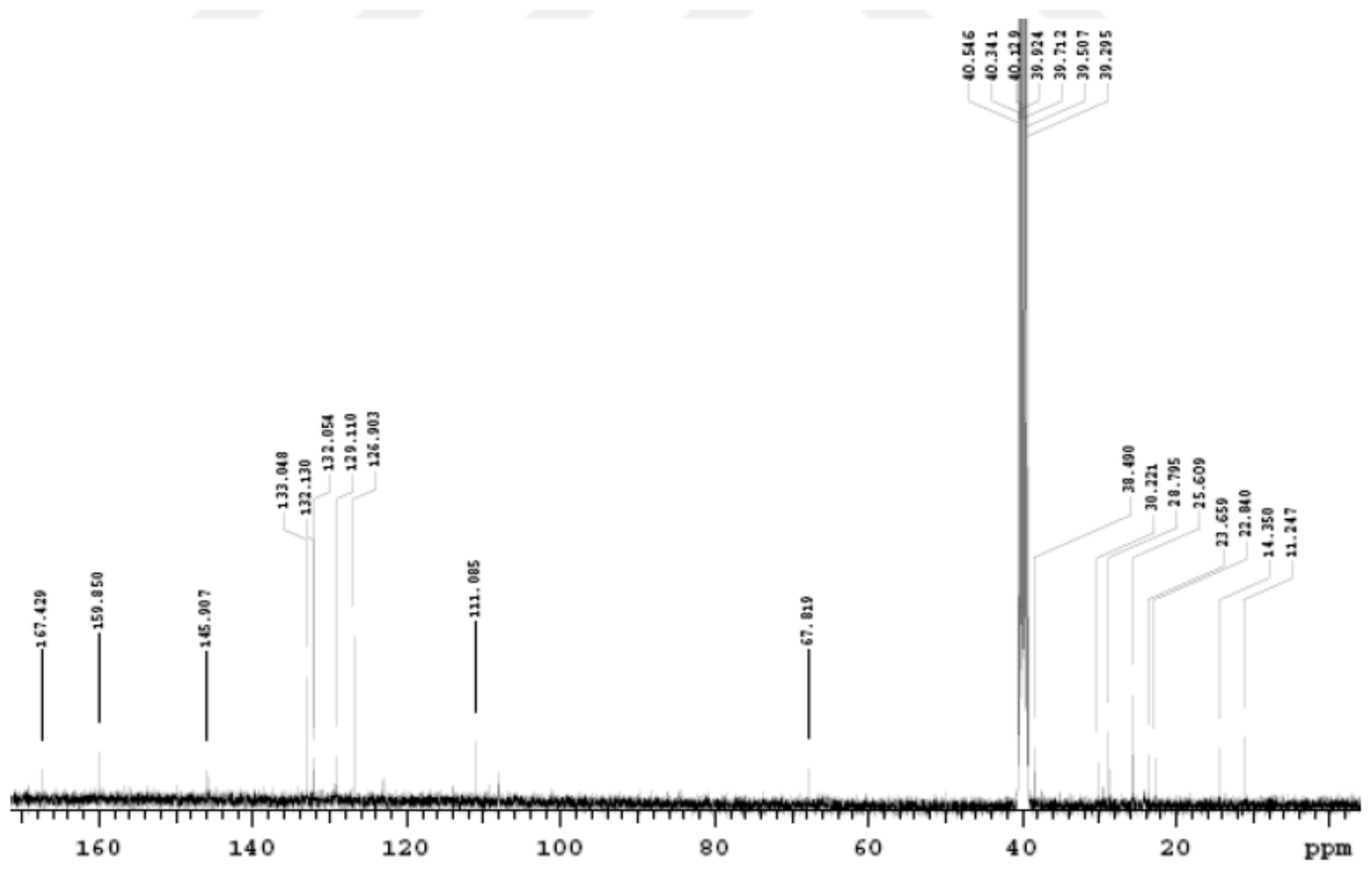

Supplement: Figure S40 — 13C Spectrum of 6,8-dibromo-2-methylquinolin-4-ol (3lb). [file tjc-48-01-0097s40.tif]

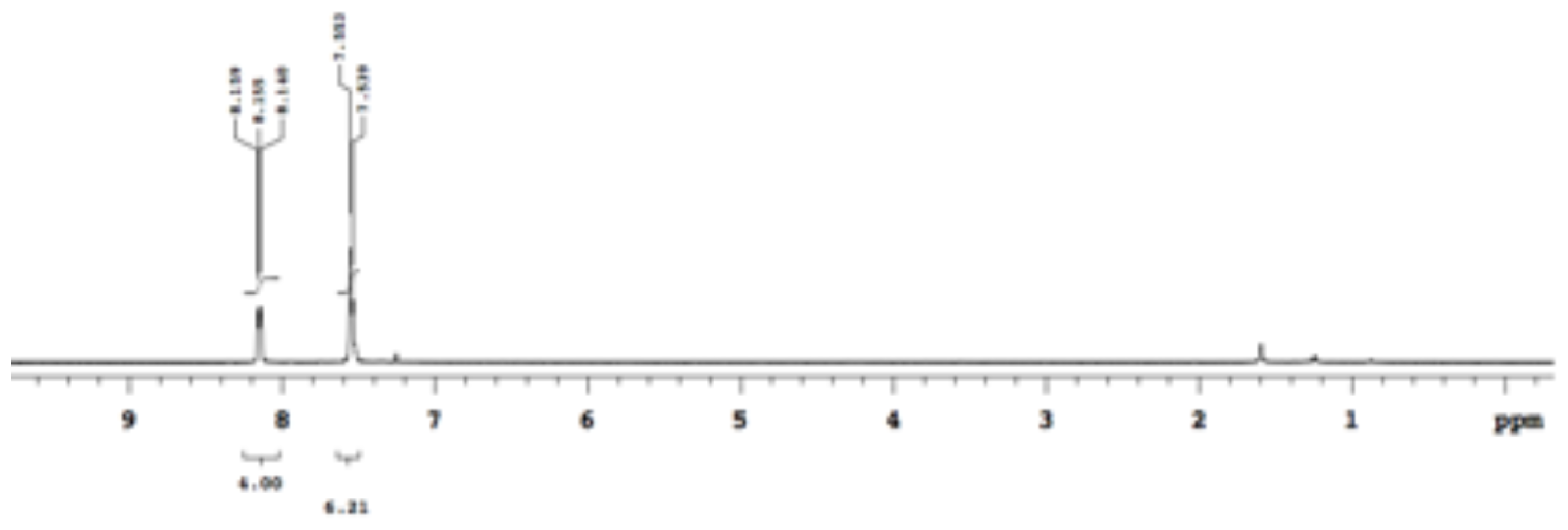

Supplement: Figure S41 — 1H Spectrum of 2,5-diphenyl-1,3,4-oxadiazole (6d′). [file tjc-48-01-0097s41.tif]

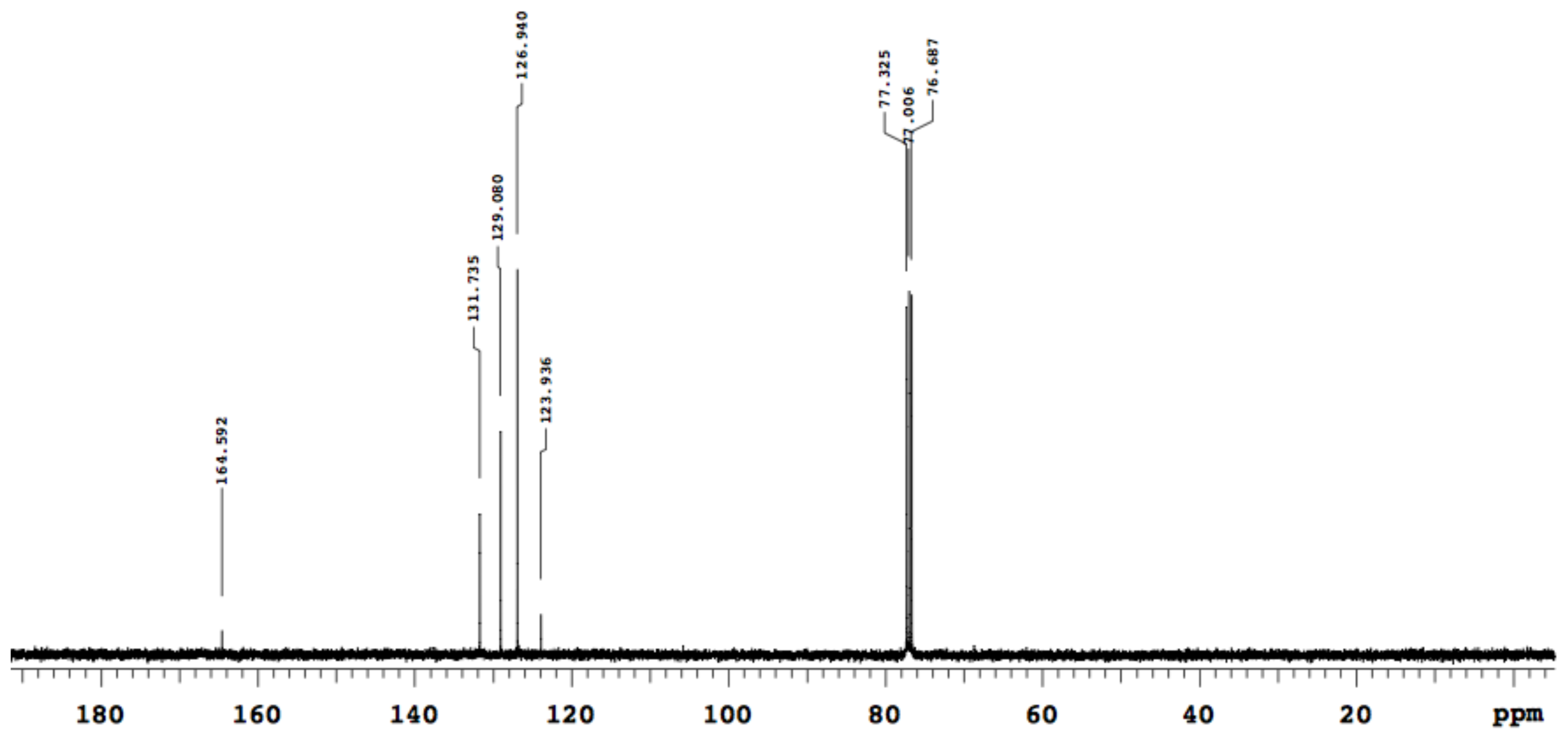

Supplement: Figure S42 — 13C Spectrum of 2,5-diphenyl-1,3,4-oxadiazole (6d′). [file tjc-48-01-0097s42.tif]

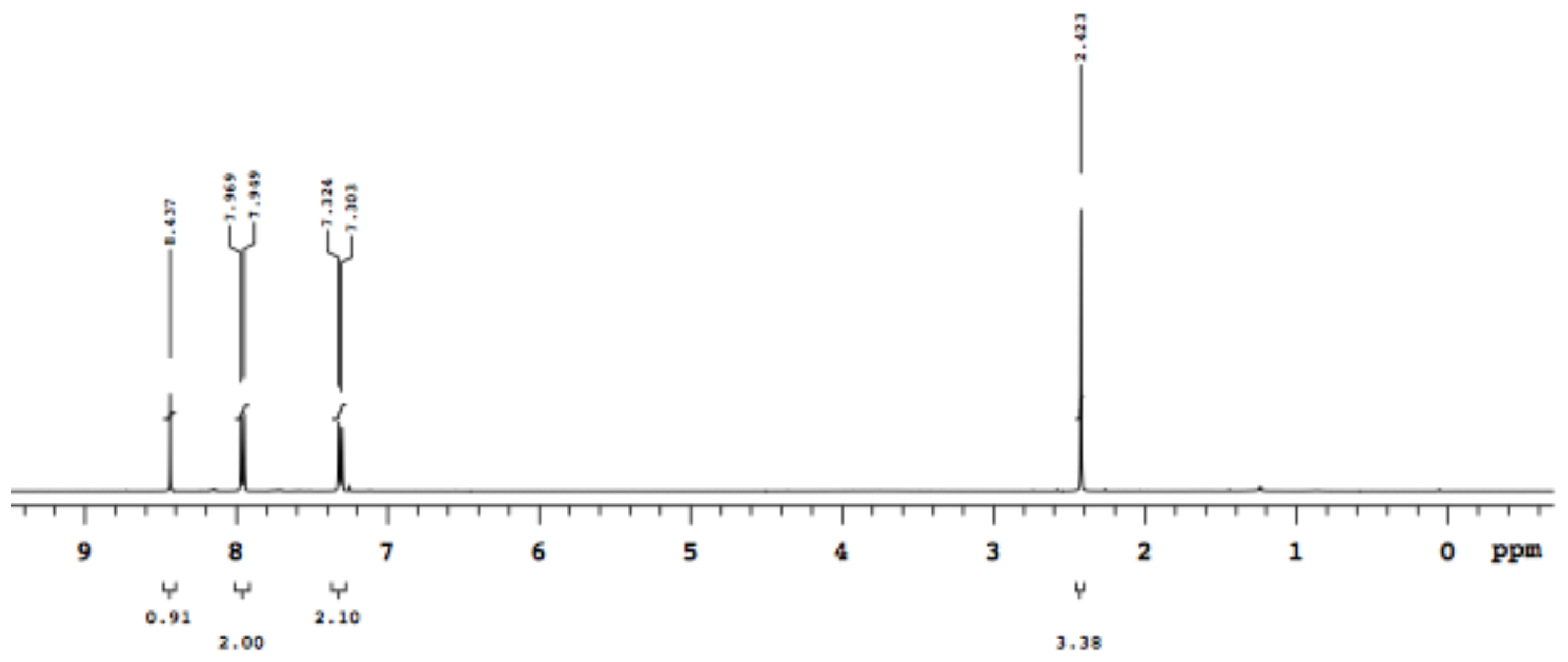

Supplement: Figure S43 — 1H Spectrum of 2-(p-Tolyl)-1,3,4-oxadiazole (6e′). [file tjc-48-01-0097s43.tif]

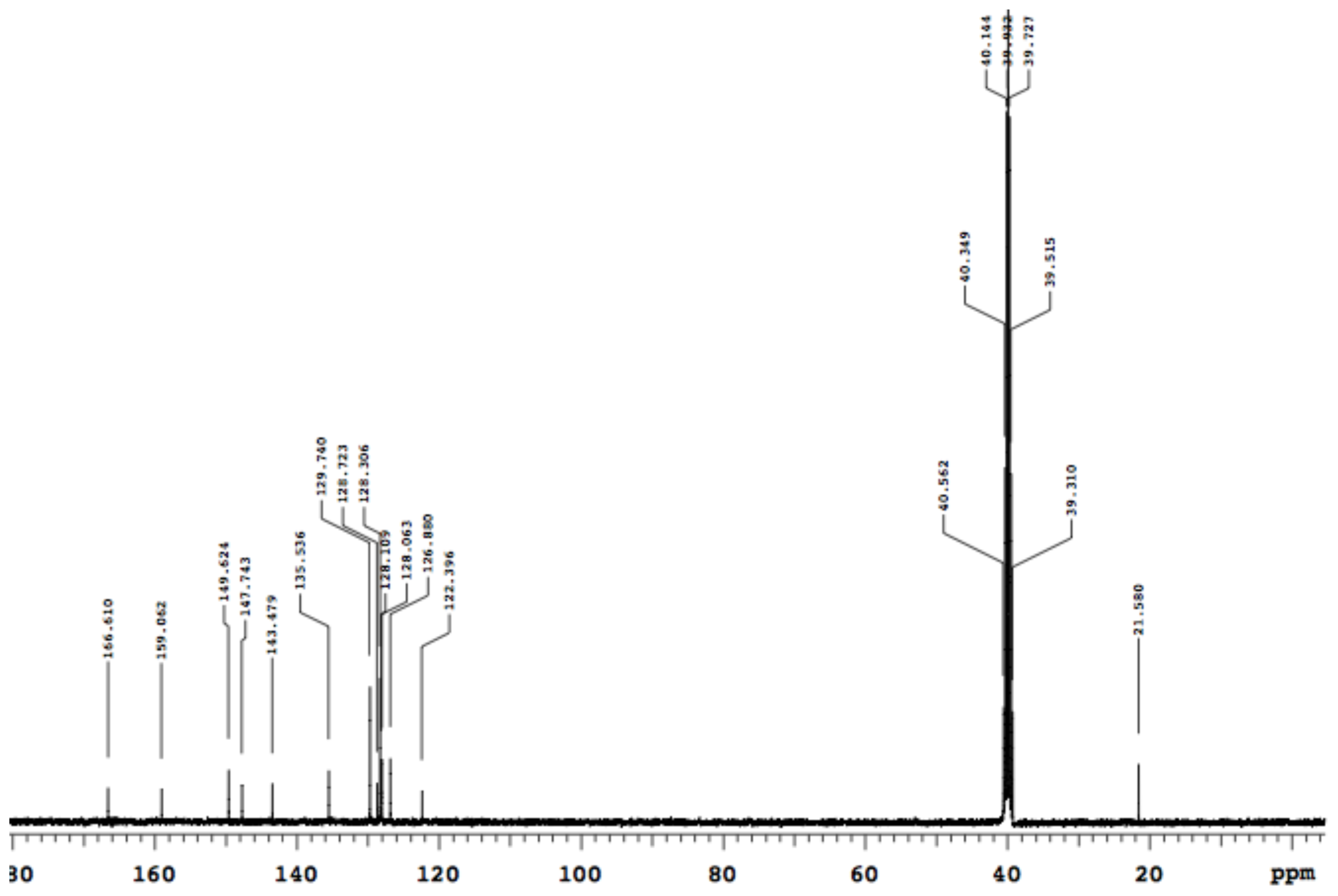

Supplement: Figure S44 — 13C Spectrum of 2-(p-Tolyl)-1,3,4-oxadiazole (6e′). [file tjc-48-01-0097s44.tif]

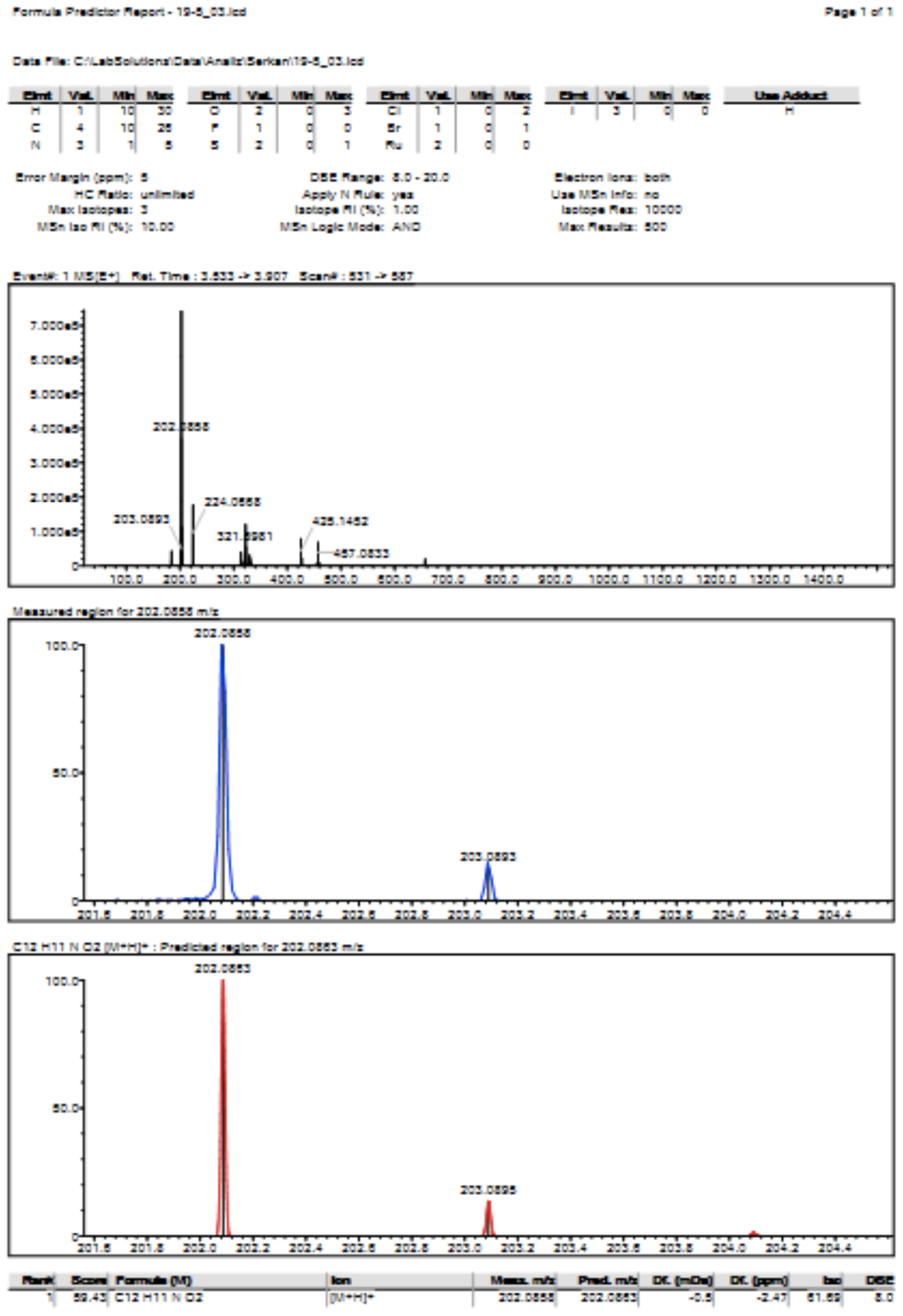

Supplement: Figure S45 — HRMS Spectrum of 1-(4-Hydroxy-2-methylquinolin-3-yl)ethanone (3a). [file tjc-48-01-0097s45.tif]

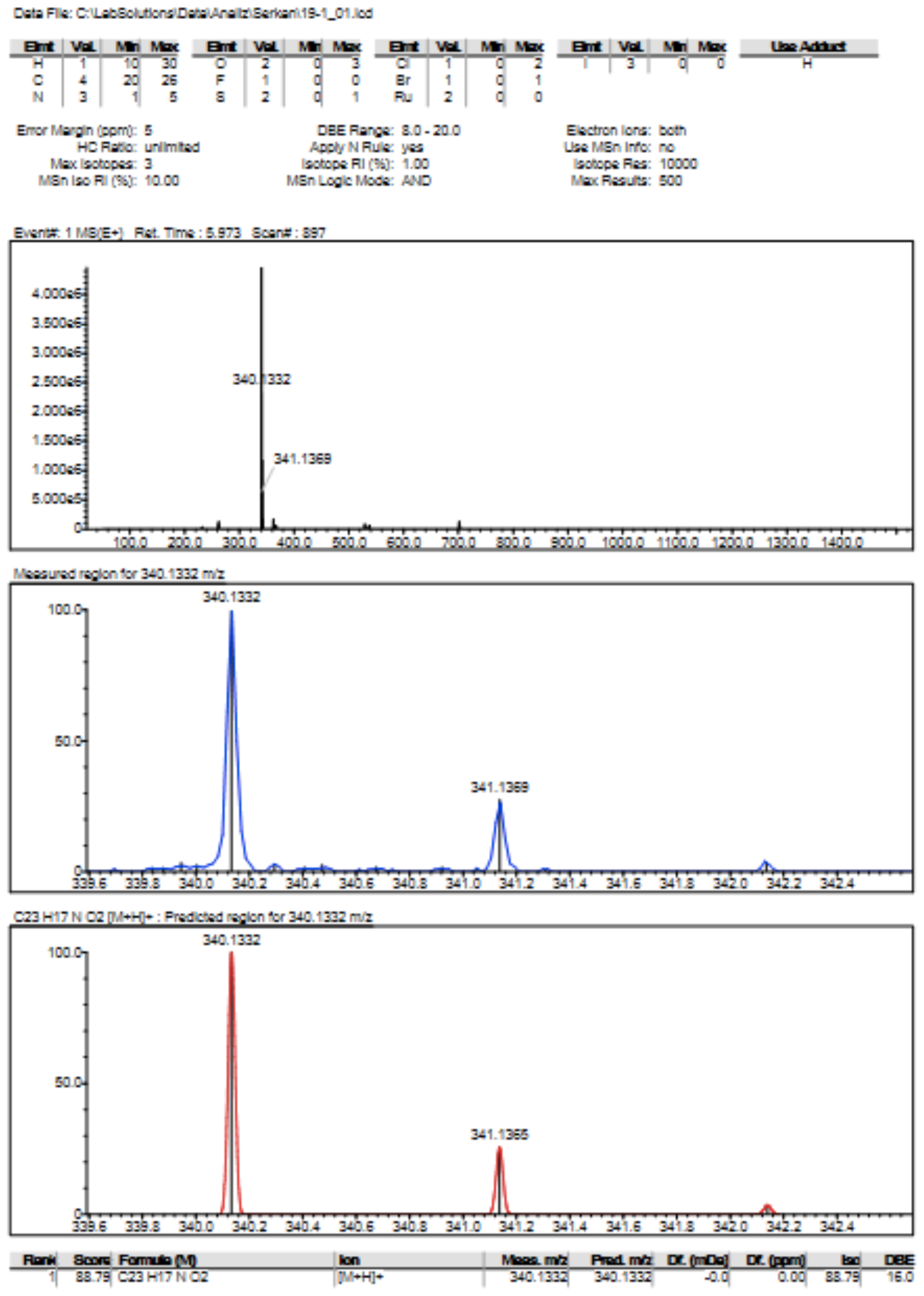

Supplement: Figure S46 — HRMS Spectrum of (4-Hydroxy-6-methyl-2-phenylquinolin-3-yl)(phenyl)methanone (3b). [file tjc-48-01-0097s46.tif]

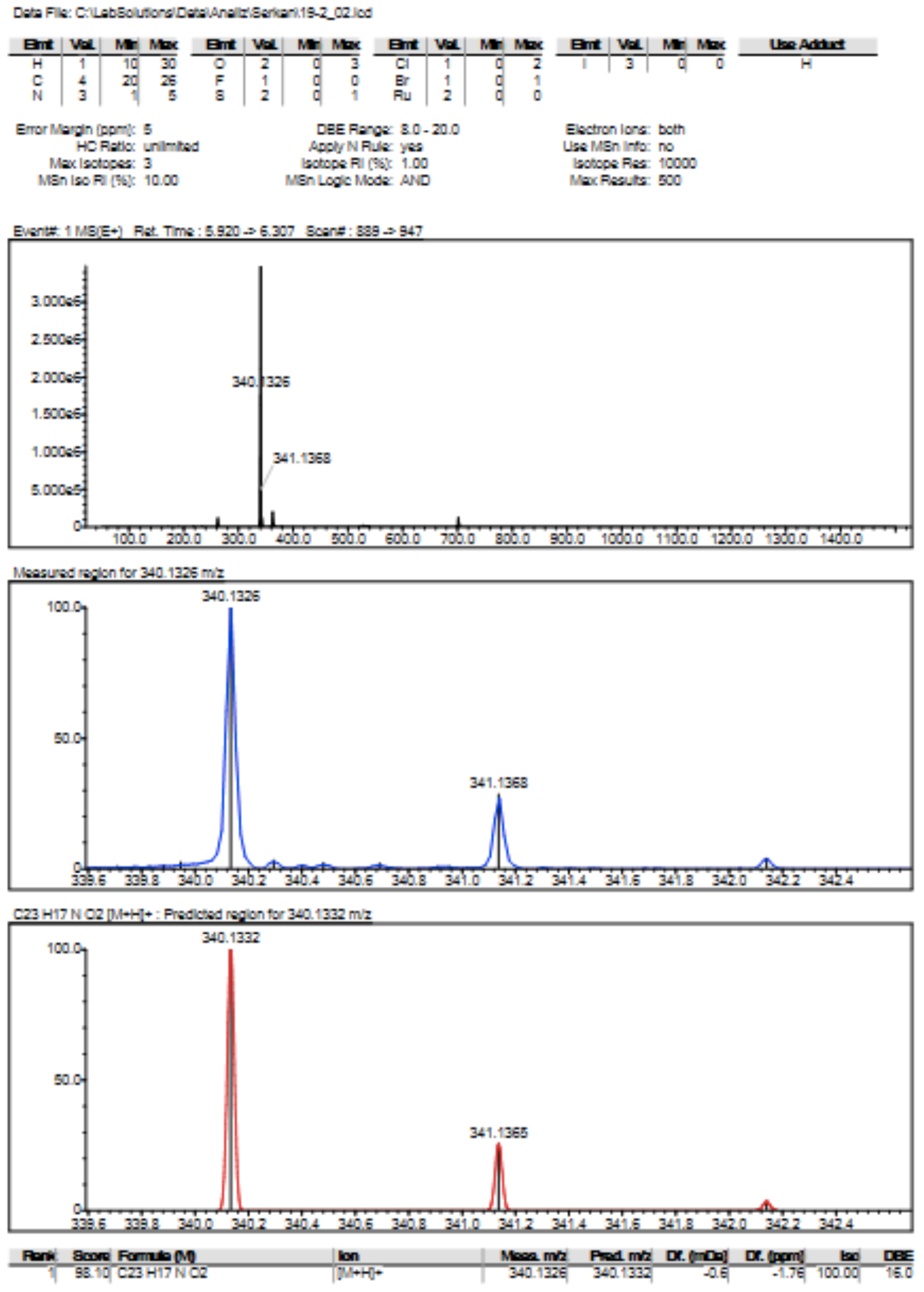

Supplement: Figure S47 — HRMS Spectrum of (4-Hydroxy-8-methyl-2-phenylquinolin-3-yl)(phenyl)methanone (3c). [file tjc-48-01-0097s47.tif]

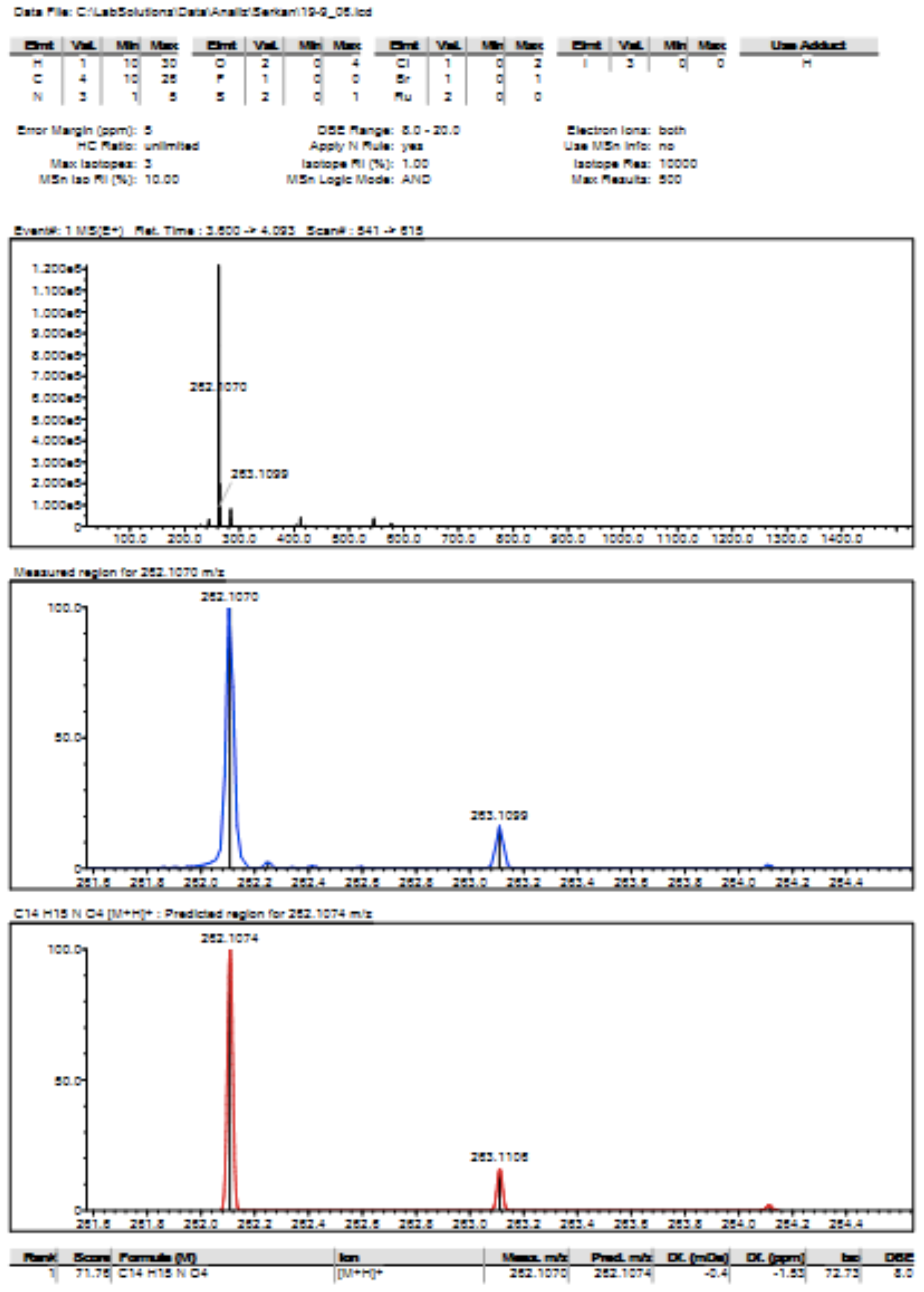

Supplement: Figure S48 — HRMS Spectrum of 1-(4-Hydroxy-6,7-dimethoxy-2-methylquinolin-3-yl)ethanone (3d). [file tjc-48-01-0097s48.tif]

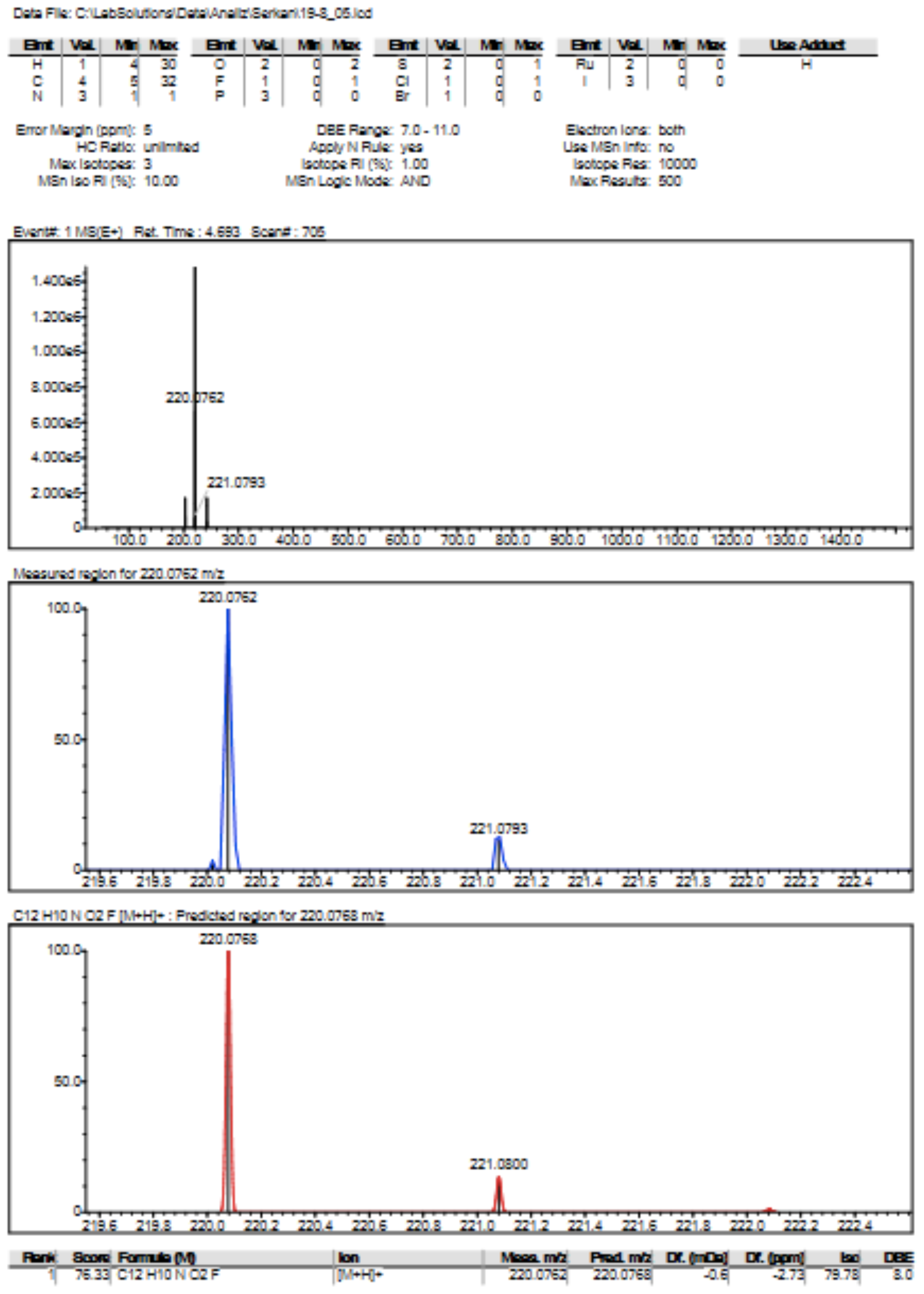

Supplement: Figure S49 — HRMS Spectrum of 1-(7-Fluoro-4-hydroxy-2-methylquinolin-3-yl)ethanone (3e). [file tjc-48-01-0097s49.tif]

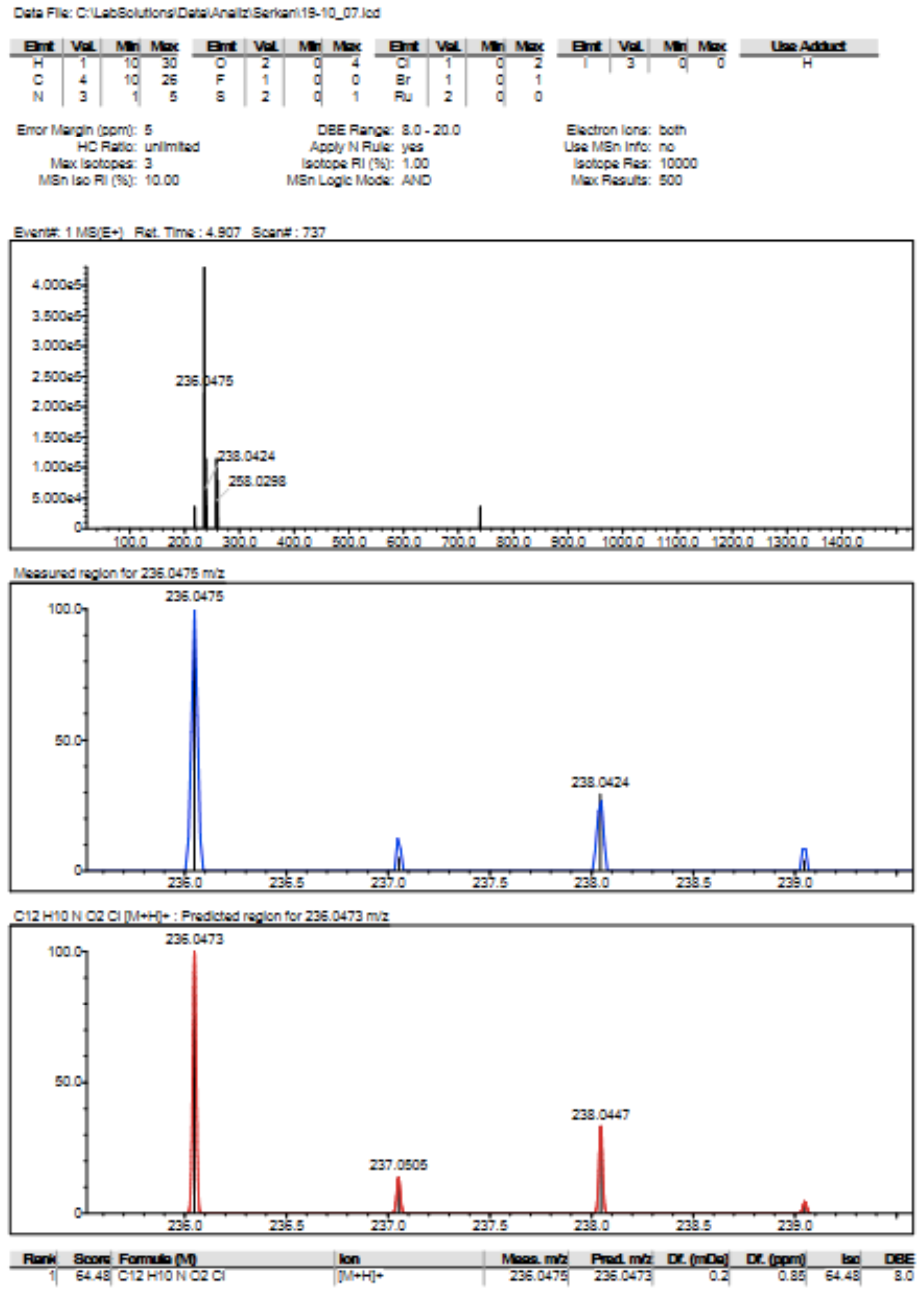

Supplement: Figure S50 — HRMS Spectrum of 1-(6-Chloro-4-hydroxy-2-methylquinolin-3-yl)ethanone (3f). [file tjc-48-01-0097s50.tif]

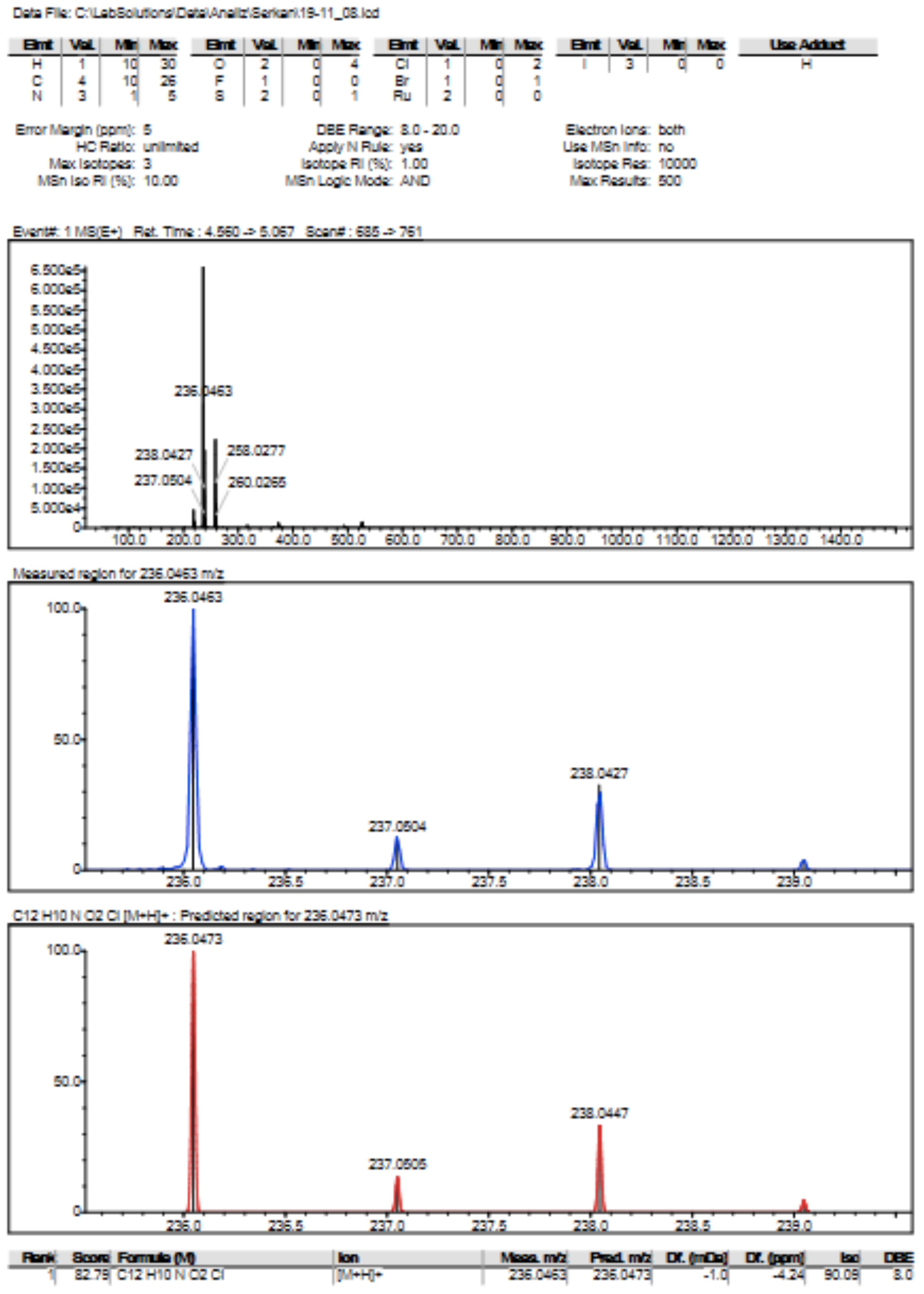

Supplement: Figure S51 — HRMS Spectrum of 1-(7-Chloro-4-hydroxy-2-methylquinolin-3-yl)ethanone (3g). [file tjc-48-01-0097s51.tif]

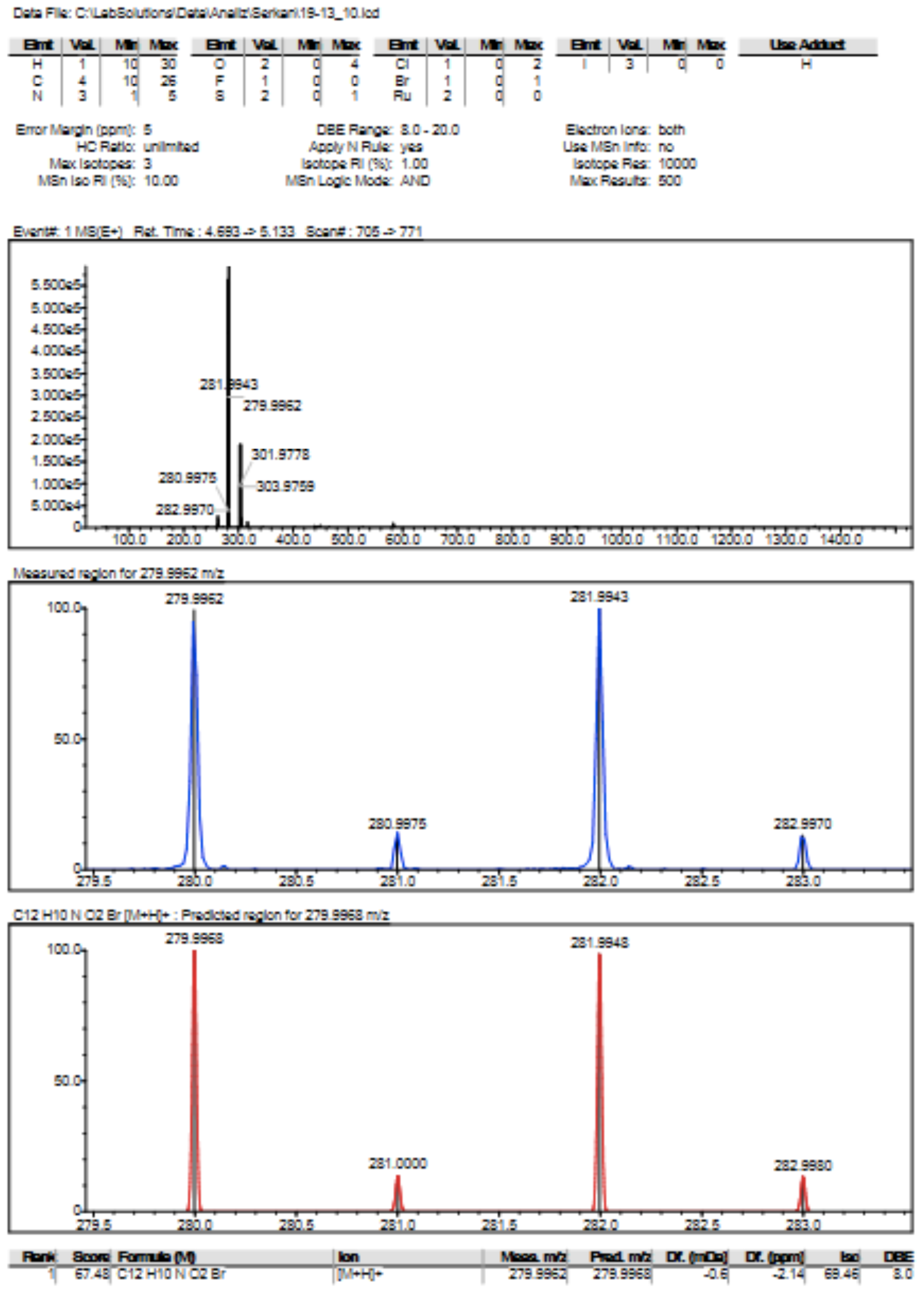

Supplement: Figure S52 — HRMS Spectrum of 1-(6-Bromo-4-hydroxy-2-methylquinolin-3-yl)ethanone (3h). [file tjc-48-01-0097s52.tif]

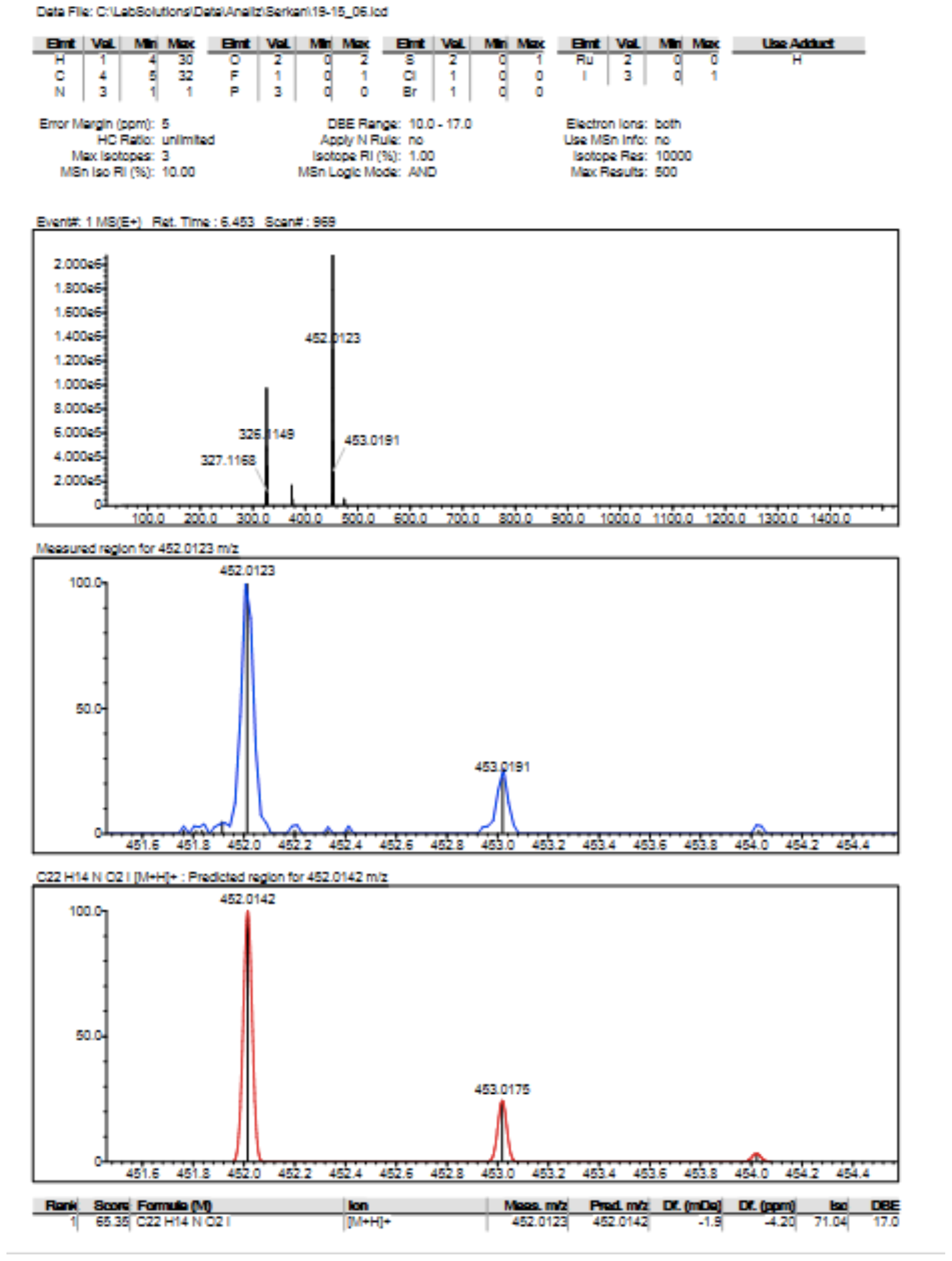

Supplement: Figure S53 — HRMS Spectrum of (4-Hydroxy-6-iodo-2-phenylquinolin-3-yl)(phenyl)methanone (3i). [file tjc-48-01-0097s53.tif]

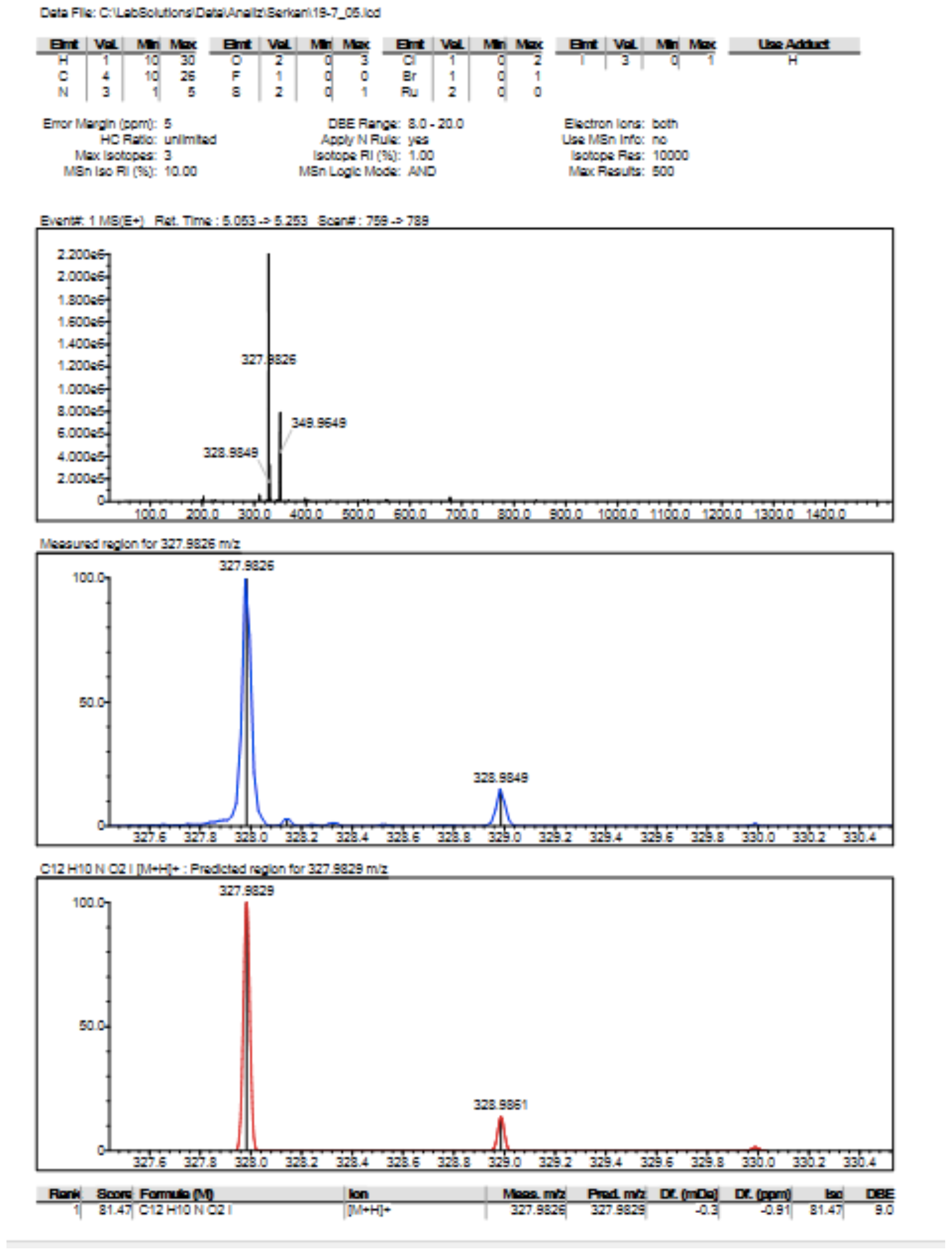

Supplement: Figure S54 — HRMS Spectrum of 1-(4-Hydroxy-6-iodo-2-methylquinolin-3-yl)ethanone (3j). [file tjc-48-01-0097s54.tif]

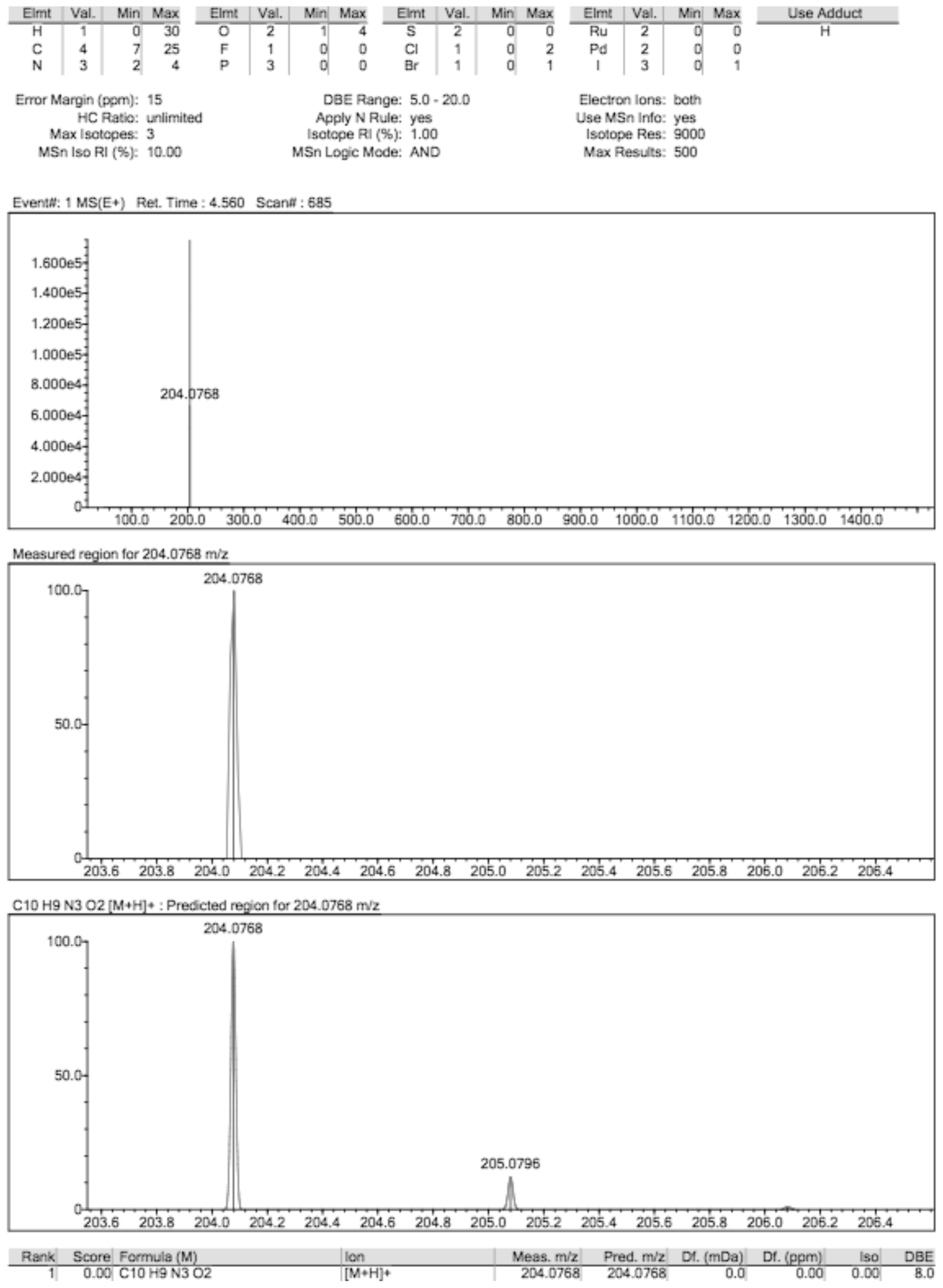

Supplement: Figure S55 — HRMS Spectrum of N-(4-Oxoquinazolin-3(4H)-yl)acetamide (6a). [file tjc-48-01-0097s55.tif]

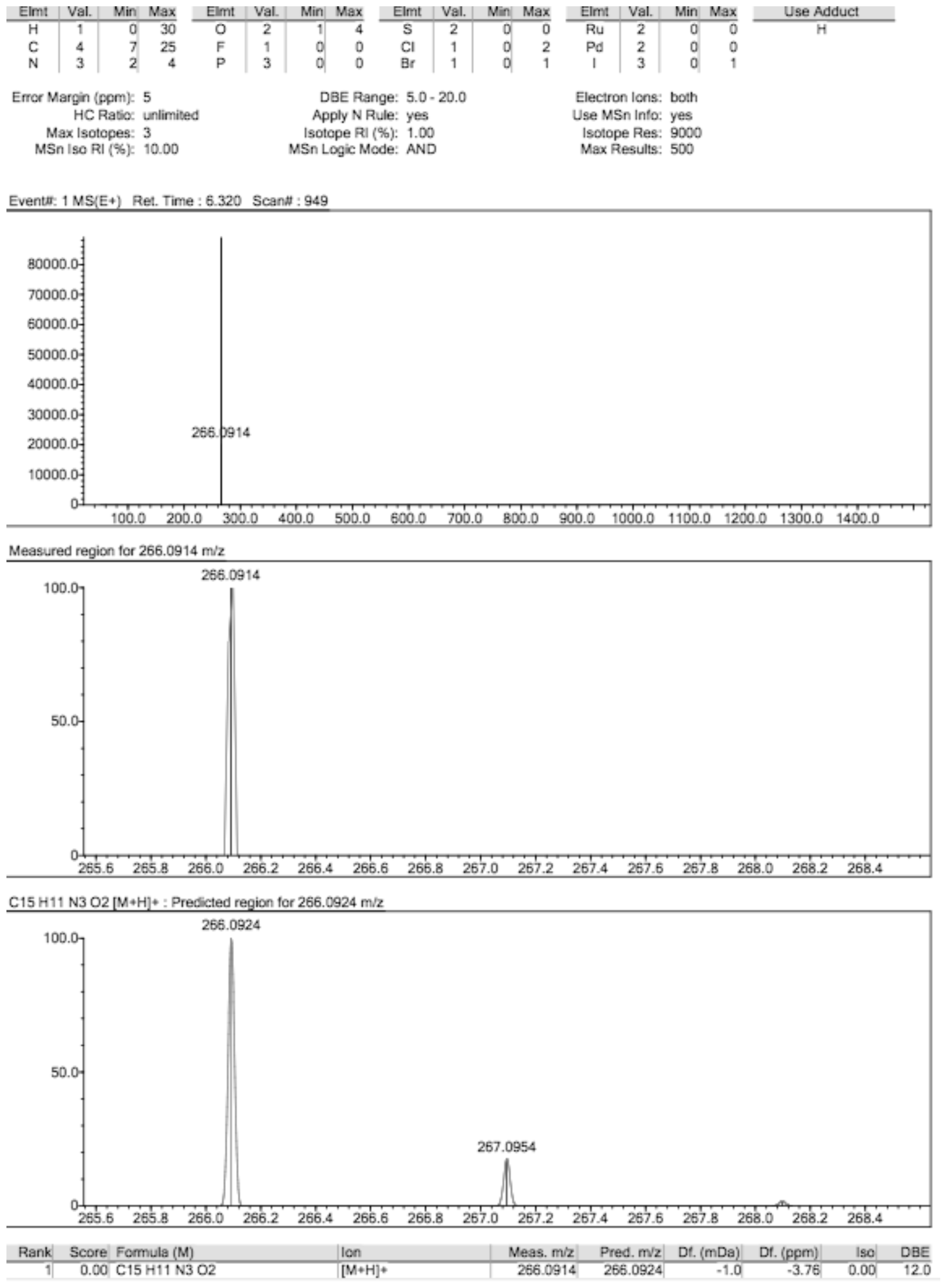

Supplement: Figure S56 — HRMS Spectrum of N-(4-Oxoquinazolin-3(4H)-yl)benzamide (6b). [file tjc-48-01-0097s56.tif]

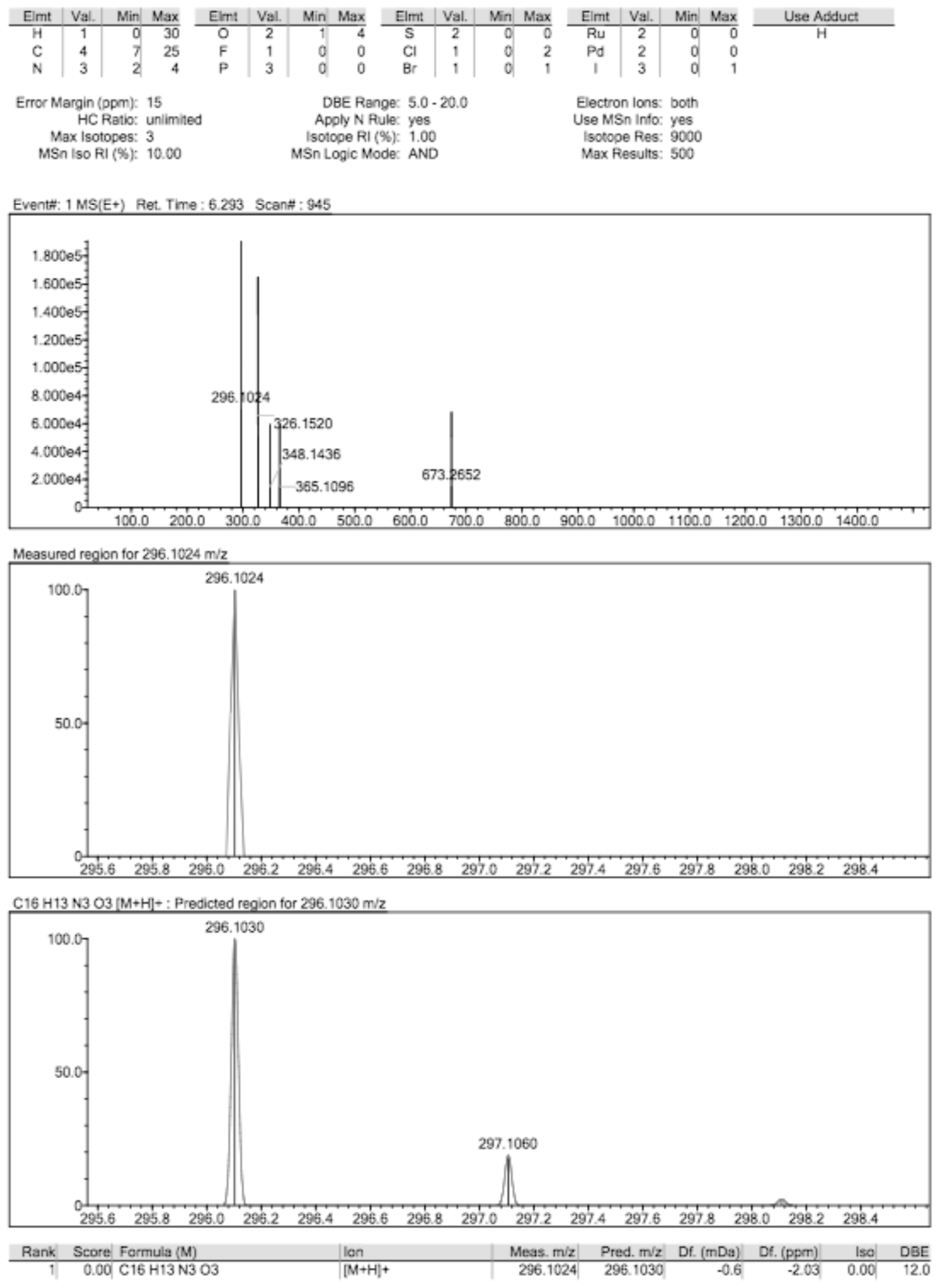

Supplement: Figure S57 — HRMS Spectrum of 4-Methoxy-N-(4-oxoquinazolin-3(4H)-yl)benzamide (6c). [file tjc-48-01-0097s57.tif]

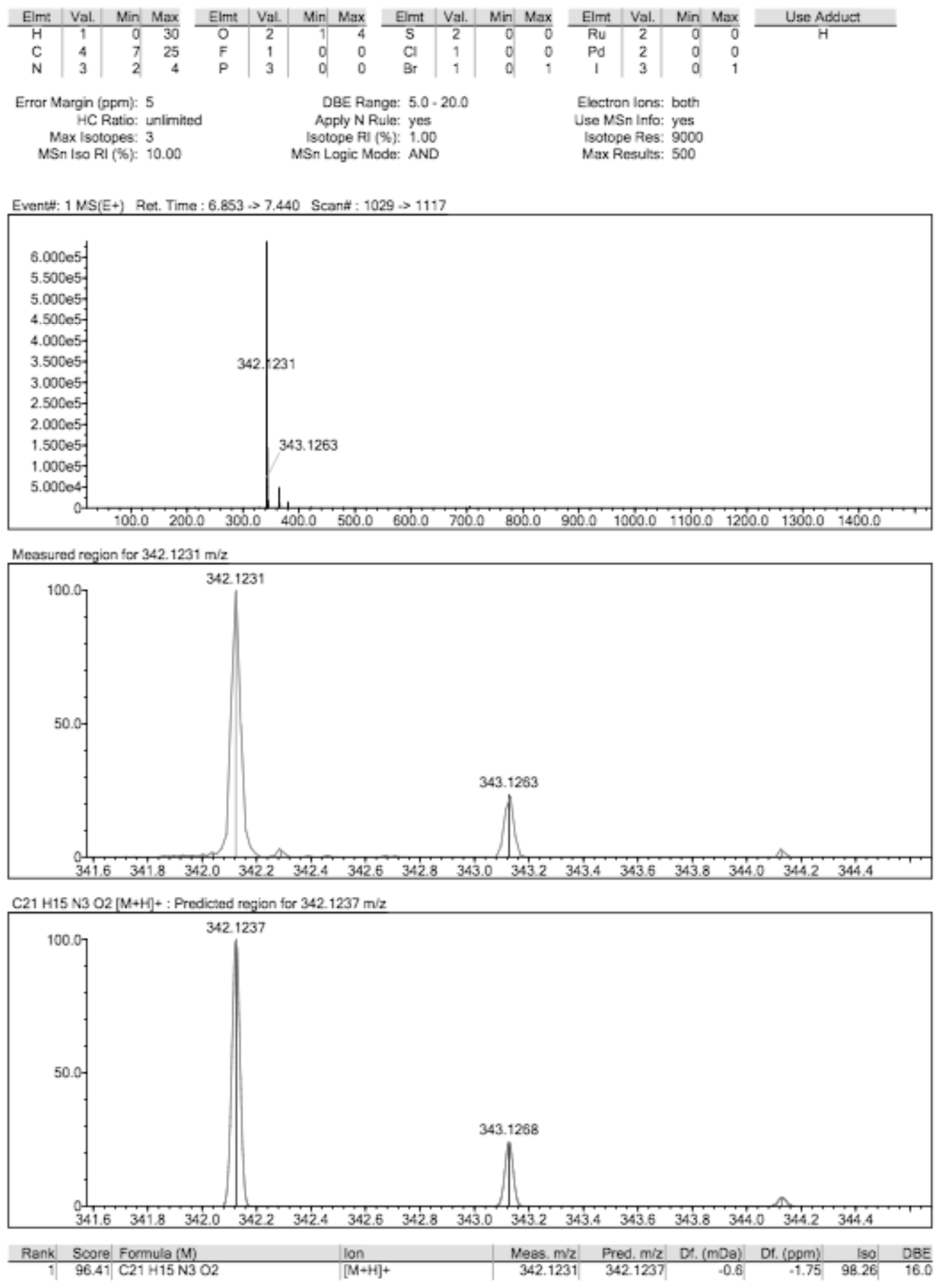

Supplement: Figure S58 — HRMS Spectrum of N-(4-Oxo-2-phenylquinazolin-3(4H)-yl)benzamide (6d). [file tjc-48-01-0097s58.tif]

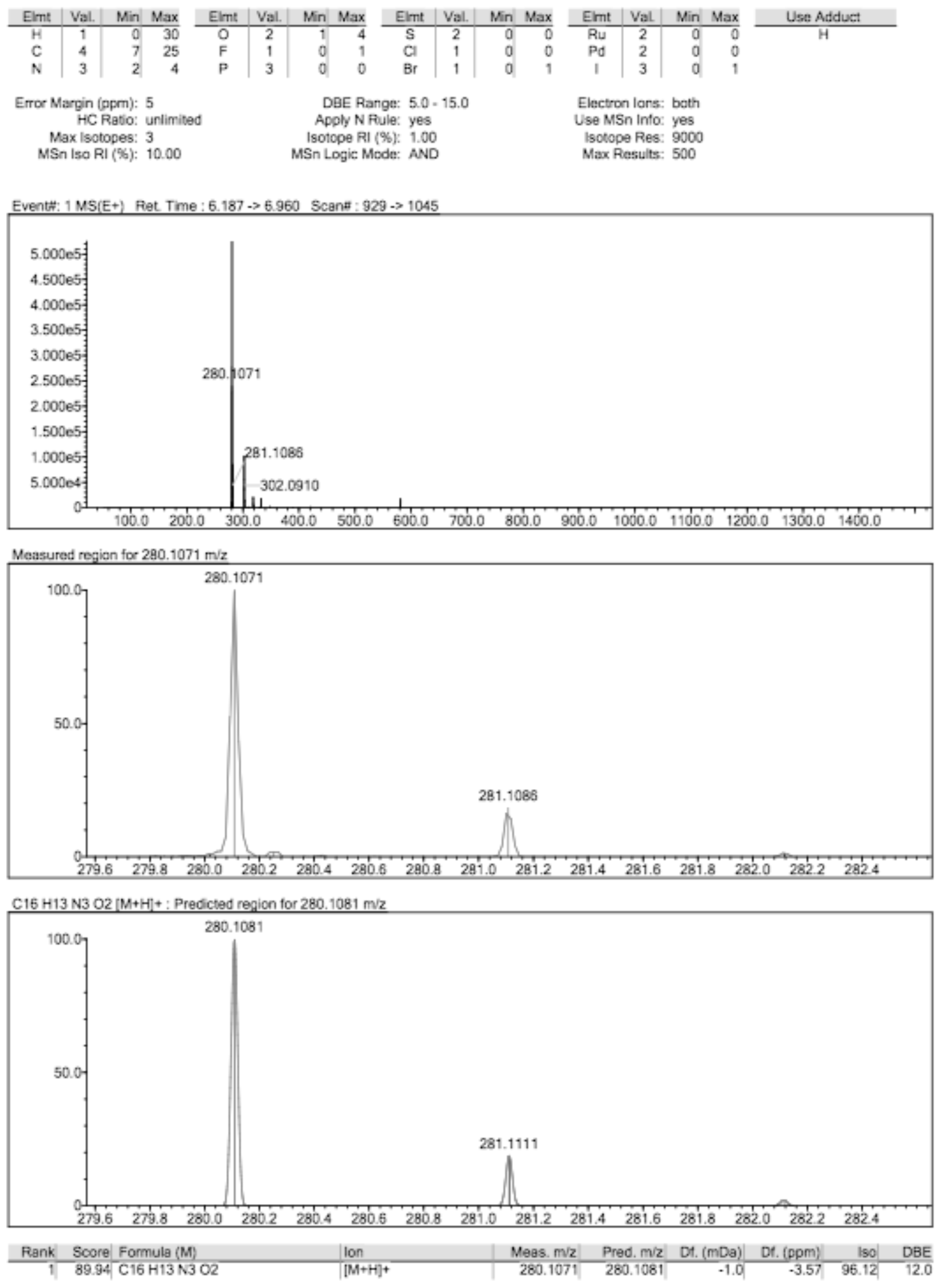

Supplement: Figure S59 — HRMS Spectrum of 4-Methyl-N-(4-oxoquinazolin-3(4H)-yl)benzamide (6e). [file tjc-48-01-0097s59.tif]

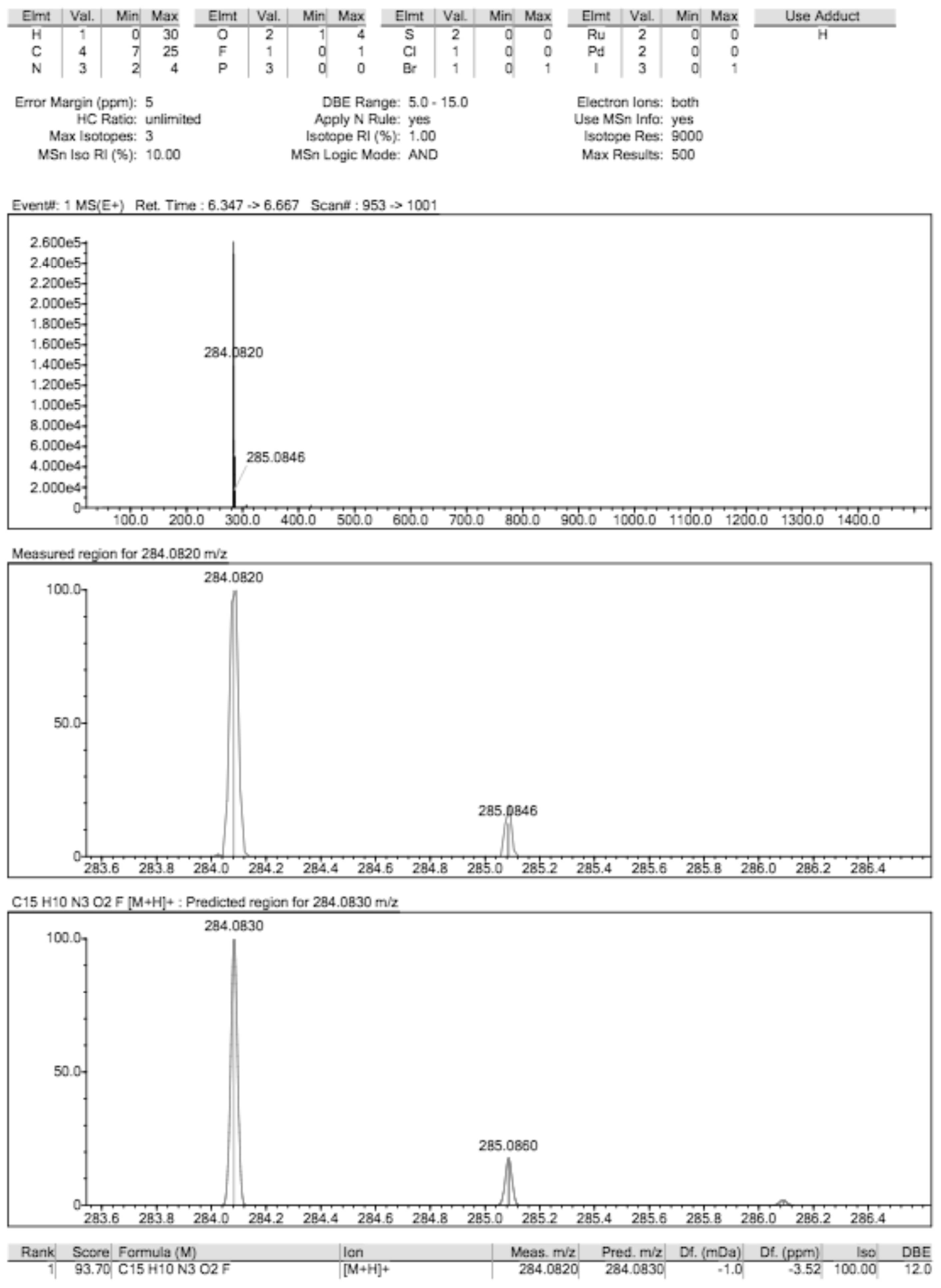

Supplement: Figure S60 — HRMS Spectrum of N-(7-Fluoro-4-oxoquinazolin-3(4H)-yl)benzamide (6f). [file tjc-48-01-0097s60.tif]

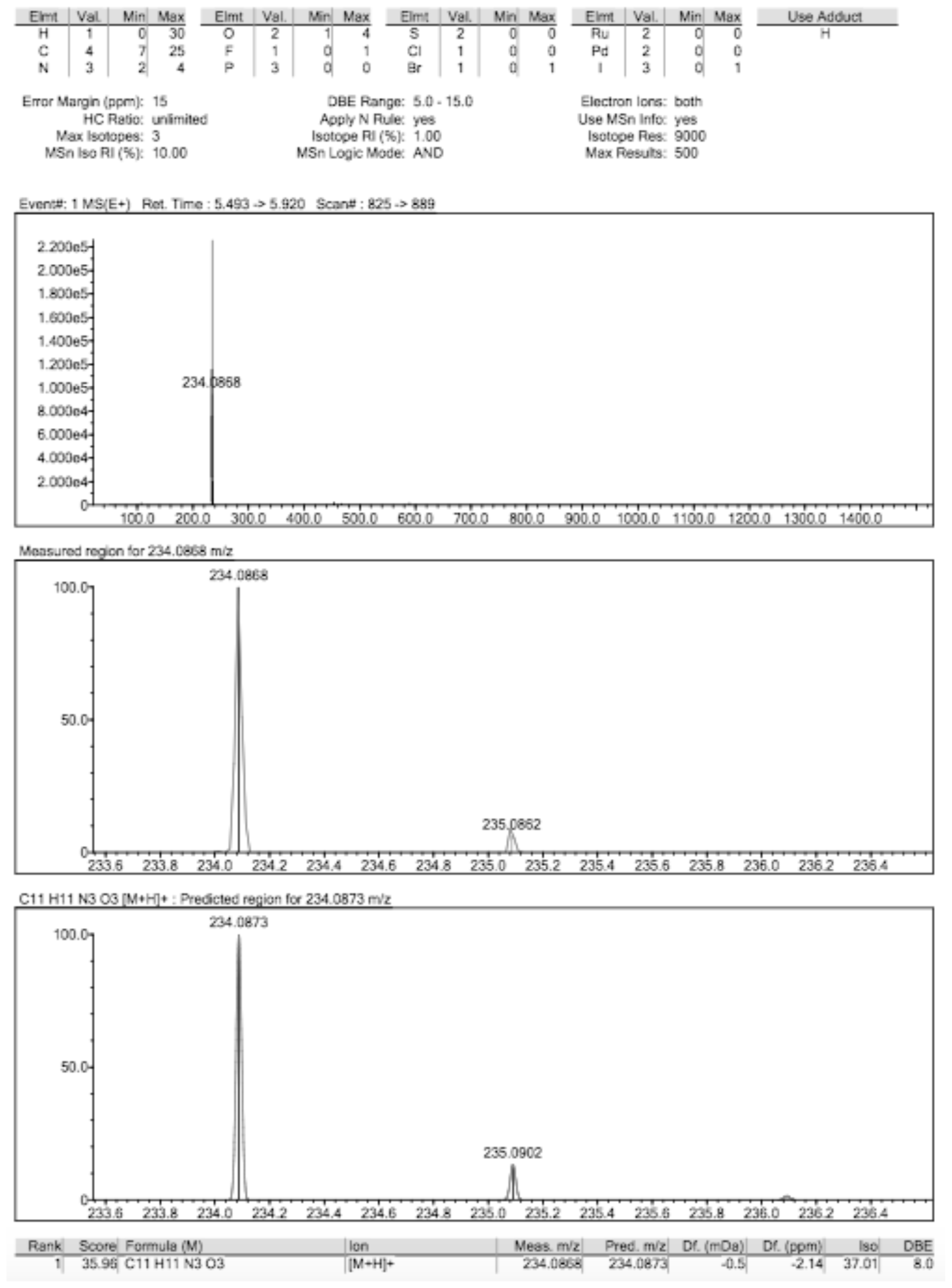

Supplement: Figure S61 — HRMS Spectrum of Ethyl (4-oxoquinazolin-3(4H)-yl)carbamate (6g). [file tjc-48-01-0097s61.tif]

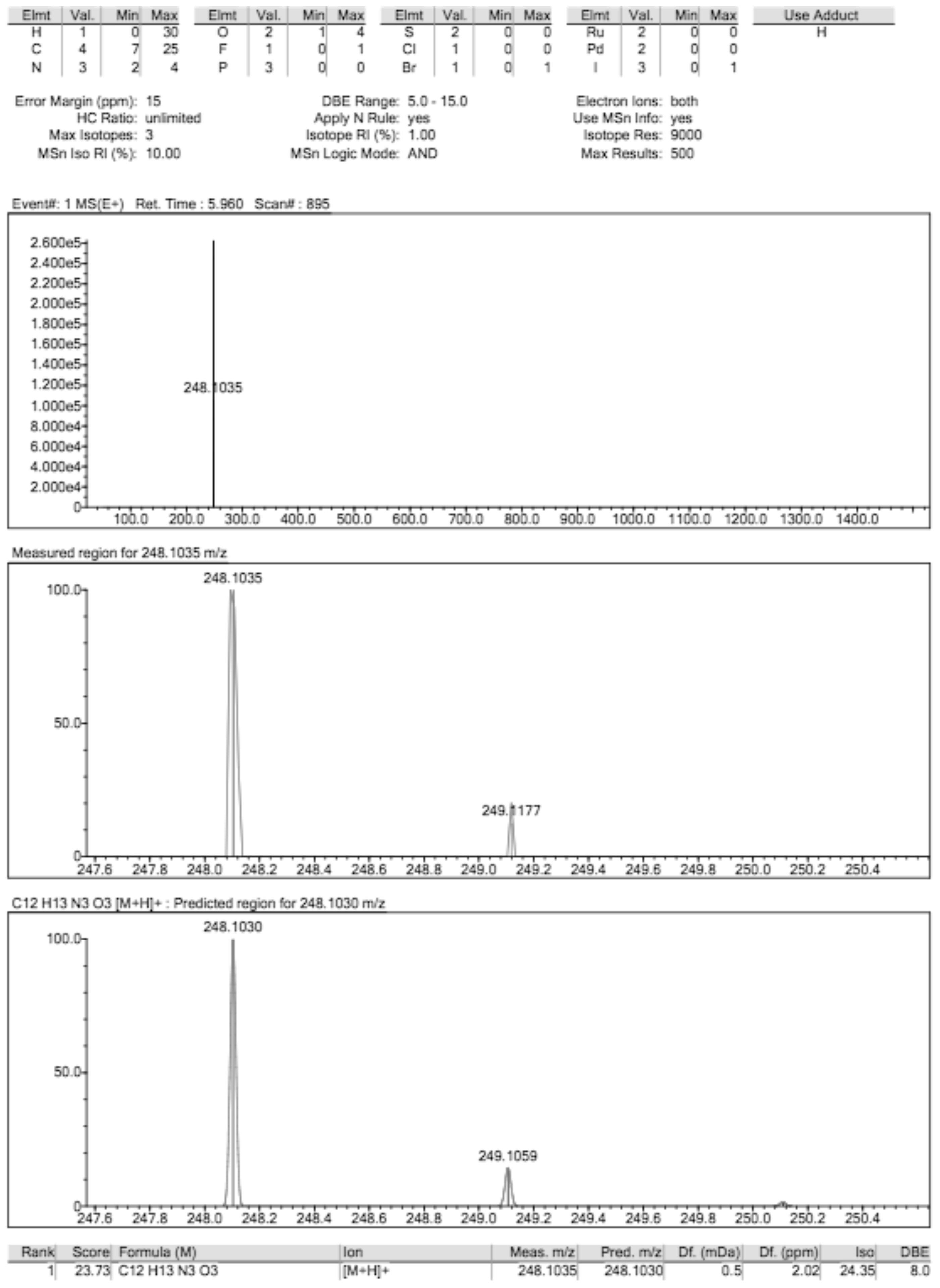

Supplement: Figure S62 — HRMS Spectrum of Ethyl (2-methyl-4-oxoquinazolin-3(4H)-yl)carbamate (6h). [file tjc-48-01-0097s62.tif]

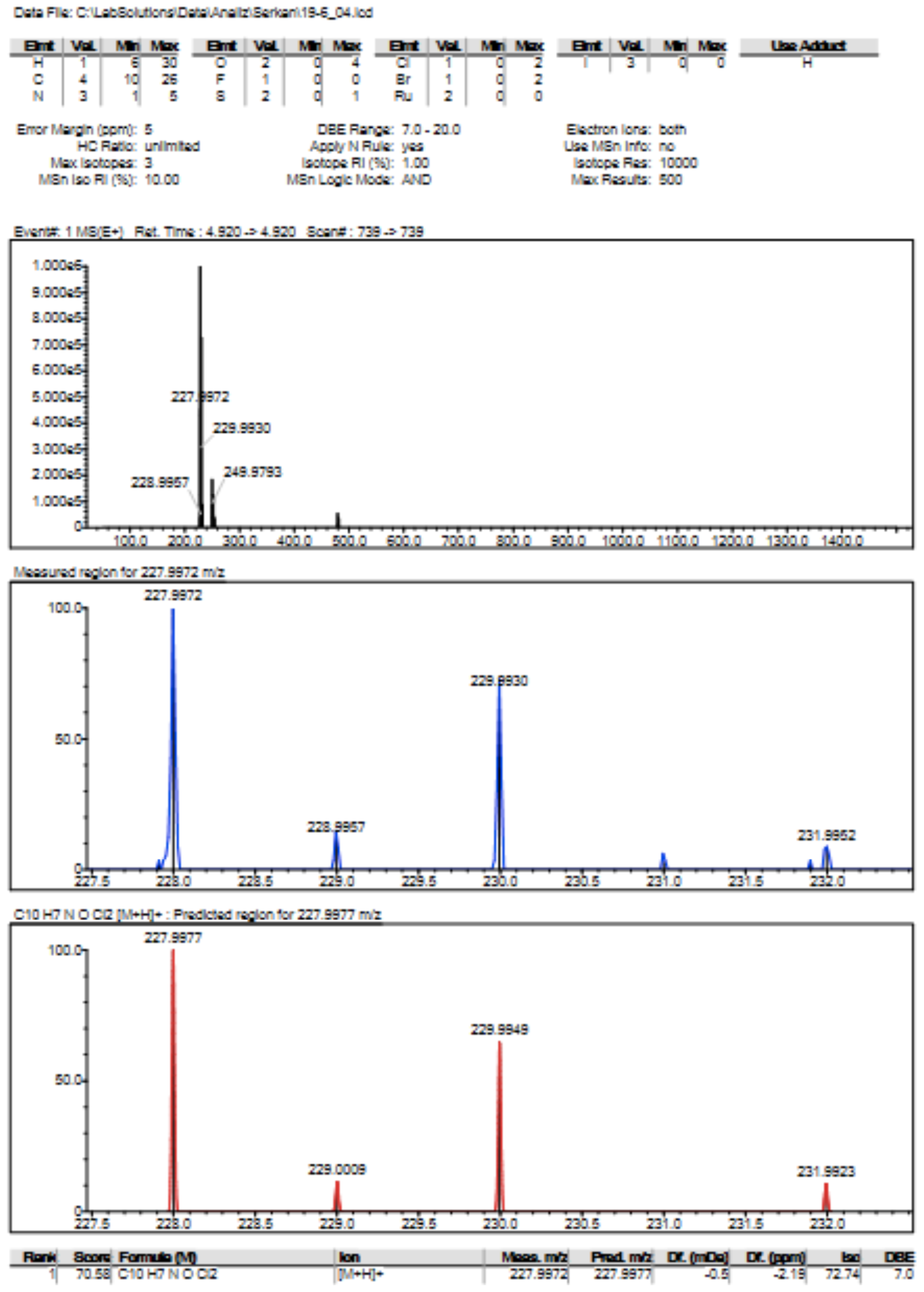

Supplement: Figure S63 — HRMS Spectrum of 6,8-dichloro-2-methylquinolin-4-ol (3kb). [file tjc-48-01-0097s63.tif]

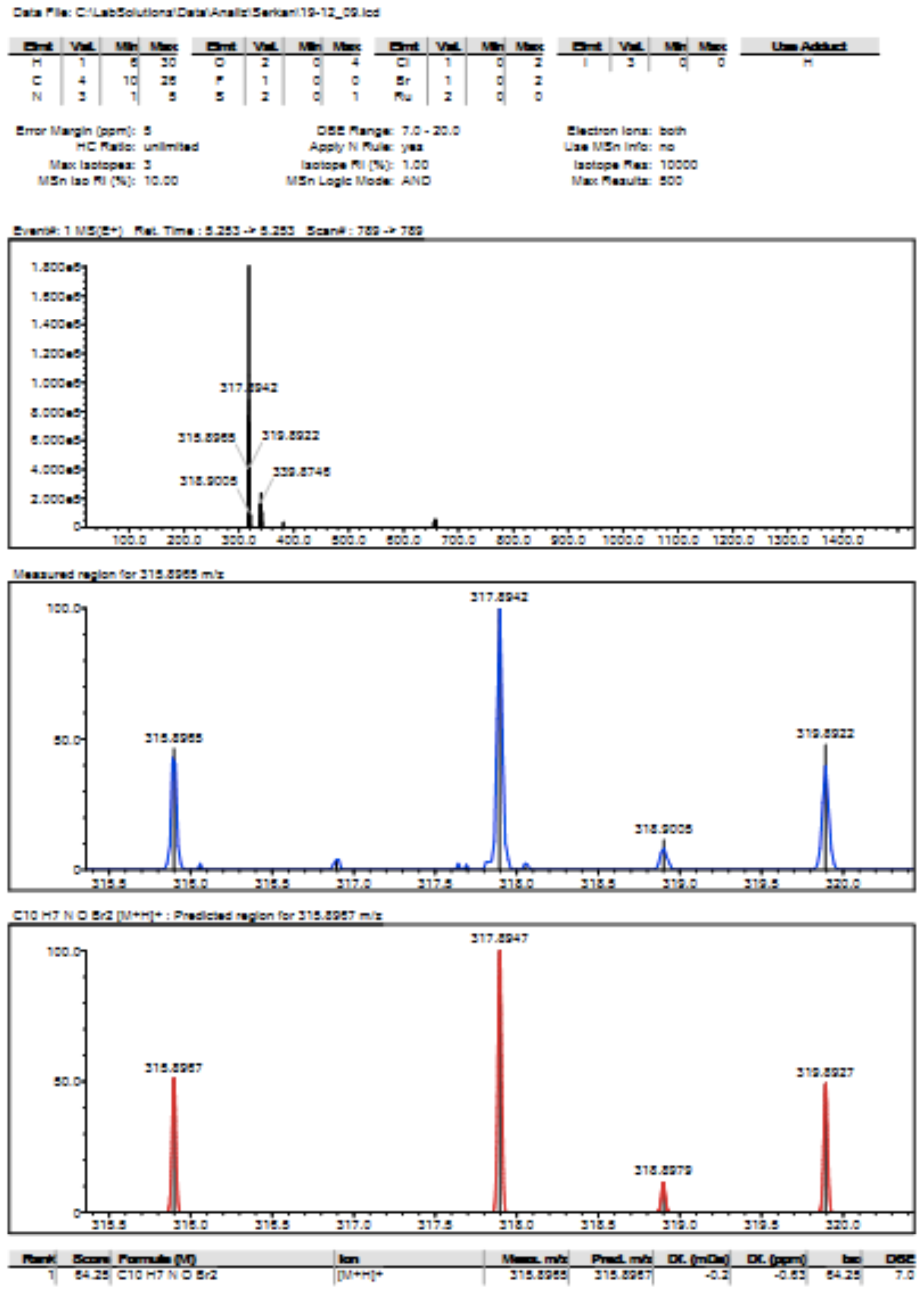

Supplement: Figure S64 — HRMS Spectrum of 6,8-dibromo-2-methylquinolin-4-ol (3lb). [file tjc-48-01-0097s64.tif]

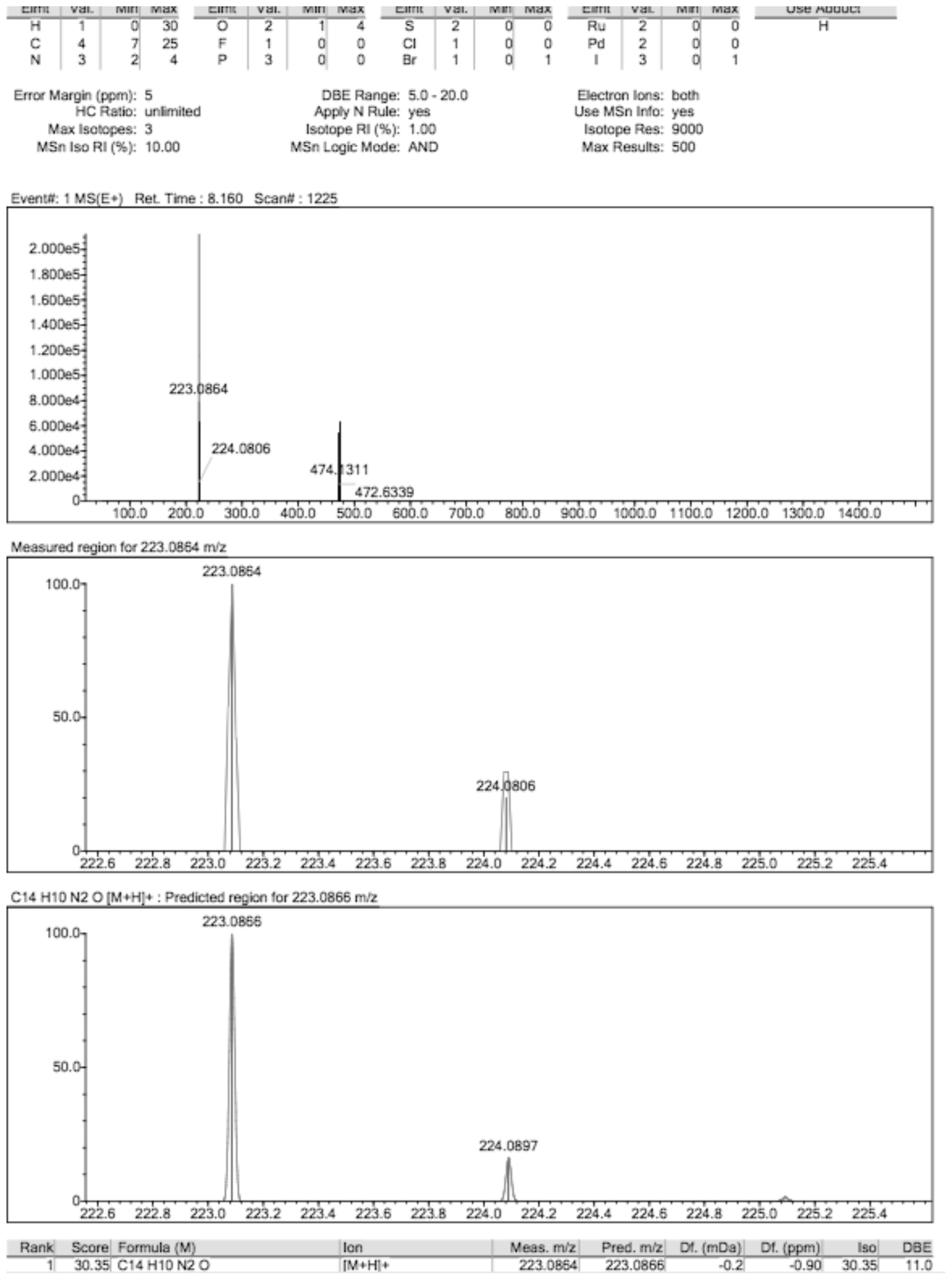

Supplement: Figure S65 — HRMS Spectrum of 2,5-diphenyl-1,3,4-oxadiazole (6d′). [file tjc-48-01-0097s65.tif]

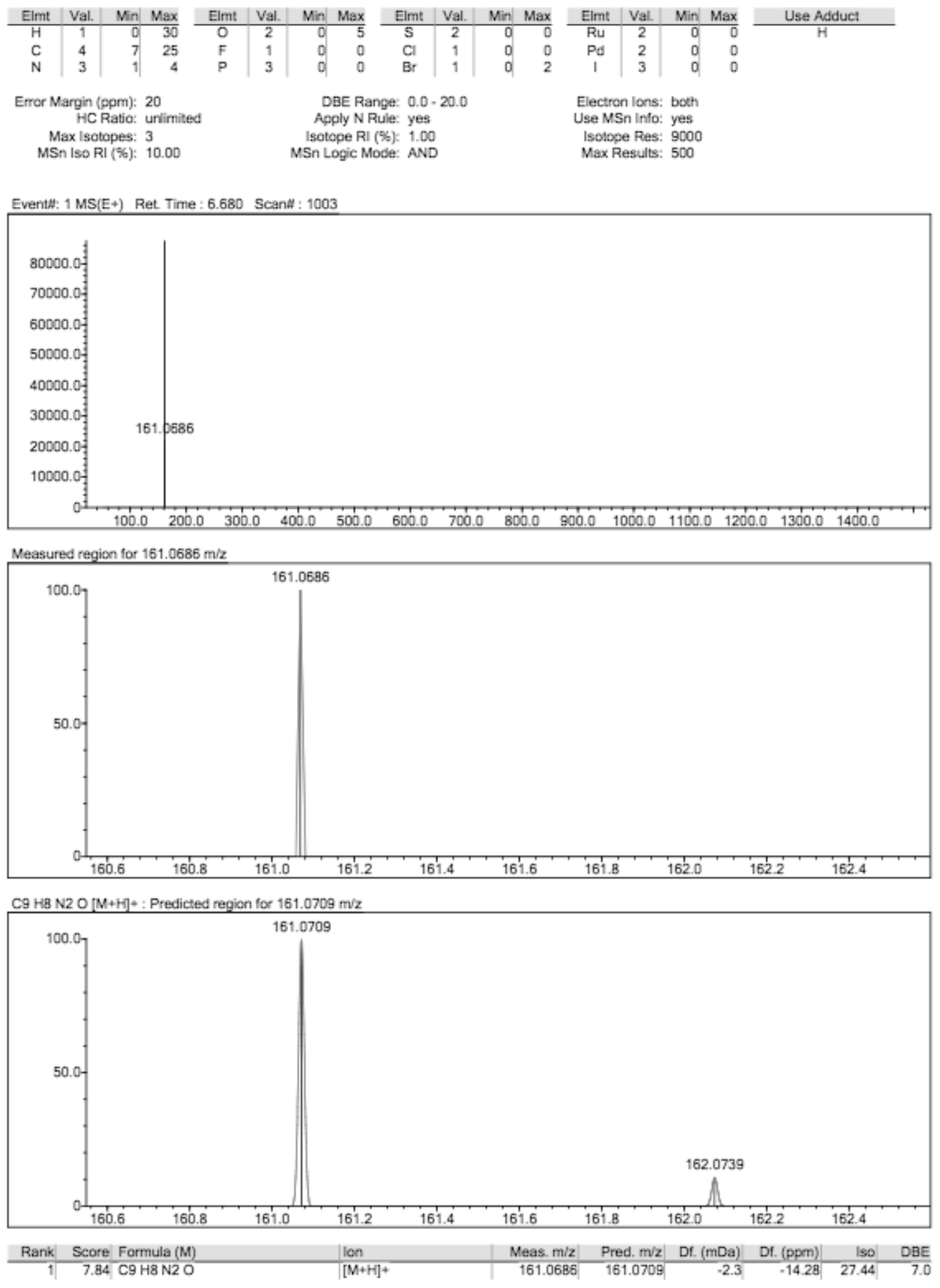

Supplement: Figure S66 — HRMS Spectrum of 2-(p-Tolyl)-1,3,4-oxadiazole (6e′). [file tjc-48-01-0097s66.tif]
